# Minimizing DNA trapping while maintaining activity inhibition via selective PARP1 degrader

**DOI:** 10.1038/s41419-024-07277-2

**Published:** 2024-12-18

**Authors:** Li Chen, Yahui Zou, Renhong Sun, Mei Huang, Xiaotong Zhu, Xiao Tang, Xiaobao Yang, Dake Li, Gaofeng Fan, Yu Wang

**Affiliations:** 1 School of Life Science and Technology, ShanghaiTech University, Shanghai, China; 2 Gluetacs Therapeutics (Shanghai) Co, Ltd, Pudong District, Shanghai China; 3 Department of Gynecology, Nanjing Women and Children’s Healthcare Hospital, Nanjing, China; 4 Shanghai Clinical Research and Trial Center, Shanghai, China; 5 Department of Gynecology, Shanghai First Maternity and Infant Hospital, School of Medicine, Tongji University, Shanghai, China; 6 Shanghai Key Laboratory of Maternal Fetal Medicine, Shanghai Institute of Maternal-Fetal Medicine and Gynecologic Oncology, Clinical and Translational Research Center, Shanghai First Maternity and Infant Hospital, School of Medicine, Tongji University, Shanghai, China

**Keywords:** Drug development, Drug safety

## Abstract

Poly (ADP-ribose) polymerase 1 (PARP1) catalyzes poly (ADP) ribosylation reaction, one of the essential post-translational modifications of proteins in eukaryotic cells. Given that PARP1 inhibition can lead to synthetic lethality in cells with compromised homologous recombination, this enzyme has been identified as a potent target for anti-cancer therapeutics. However, the clinical application of existing PARP1 inhibitors is restrained by side effects associated with DNA trapping and off-target effects, highlighting the need for improved therapeutic strategies. By integrating protein degradation technology, we synthesized a PROTAC molecule 180055 based on the Rucaparib junction and VHL ligand, which efficiently and selectively degraded PARP1 and inhibited PARP1 enzyme activity without a noticeable DNA trapping effect. Furthermore, 180055 kills tumor cells carrying *BRCA* mutations with a minor impact on the growth of normal cells both in vitro and in vivo. This suggests that 180055 is a PARP1-degrading compound with excellent pharmacological efficacy and extremely high biological safety that deserves further exploration and validation in clinical trials.

## Introduction

Poly ADP-ribose polymerase 1 (PARP1) plays a pivotal role in DNA repair [1–3]. Upon detection of DNA damage, PARP1 binds to the site of DNA breakage and catalyzes the decomposition of nicotinamide adenine dinucleotide (NAD^+^) into nicotinamide and ADP-ribose. Subsequently, it self-modifies by attaching poly ADP-ribose (PAR) chains, which recruit and post-translationally modify other DNA repair proteins to the site of damage, facilitating the repair process [4, 5]. Once the repair is complete, PARP1 disengages from the DNA lesion [5]. PARP inhibitors have been developed extensively since the discovery that they can selectively kill *BRCA*-deficient cancer cells, which are defective in homologous recombination repair, a phenomenon called synthetic lethality [6, 7]. To date, several PARP inhibitors have demonstrated efficacy in clinical trials, leading to the regulatory approval of drugs such as Olaparib, Rucaparib, Niraparib, Talazoparib, Fluzoparib, and Pamiparib [8–11].

The mechanisms by which PARP inhibitors exert their effects are twofold. Firstly, they can bind to the NAD^+^ binding pocket of PARP1, thereby inhibiting its enzymatic activity and preventing the repair of DNA single-strand breaks by PARP1 [12]. Secondly, PARP inhibitors can stabilize the DNA-PARP1 complex, impeding its disassembly. This phenomenon, known as DNA trapping, results in the persistent association of the DNA-PARP1 complex, inhibiting the repair of DNA single-strand breaks by other enzymes [13–15]. The accumulation of unrepaired single-strand breaks can lead to the formation of double-strand breaks, jeopardizing genomic integrity and cellular viability [16, 17]. Impeding DNA damage repair in highly mutated cancer cells through enzymatic activity inhibition and DNA trapping is an effective strategy for PARP inhibitors to exert anti-cancer effects. While this is particularly lethal to cancer cells with deficiencies in *BRCA* function, it can also exert toxicity to normal cells, as Olaparib and Niraparib demonstrated a dose-dependent cytotoxic effect on wild-type DT40 cells [13, 18].

PARP inhibitors are meticulously engineered to impede PARP activity by competitively binding to the enzyme’s active site with NAD^+^, thereby interfering with the DNA repair process. Due to the conserved nature of the catalytic domains across various PARP family members, PARP inhibitors usually exhibit a broader spectrum of activity that extends beyond PARP1 to include other members such as PARP2 and tankyrases (PARP5A and PARP5B) [8–11, 19]. The therapeutic efficacy of PARP inhibitors is primarily attributed to the inhibition of PARP1, which is essential for their anti-cancer properties. Concurrently, the inhibition of PARP2 has been implicated to be responsible for certain hematological side effects observed with current clinical PARP inhibitors, such as anemia [20–22]. Additionally, the inhibition of tankyrases has been linked to non-hematological toxicities, manifesting as symptoms like nausea, vomiting, and indigestion, which can impact the patient’s overall well-being and treatment experience [23, 24]. In unison, it is imperative to devise novel strategies to mitigate the activity of the PARP1 enzyme and circumvent the detrimental side effects induced by DNA trapping and multi-target engagement.

PROTAC (Proteolysis-Targeting-Chimera) represents an innovative drug development approach that leverages the Ubiquitin-Proteasome System (UPS) to degrade specific target proteins [25, 26]. This technology marks a paradigm shift in drug design, addressing the development challenges associated with traditional “non-druggable” targets. According to the latest data provided by Pharmadu - Global Pharmaceutical Big Data CIO Service Platform (https://data.pharmacodia.com/), many PROTAC molecules are advancing through clinical trials, with 2 in phase III, 4 in phase II and 33 in phase I [27]. The potential of PROTAC technology to address the limitations of PARP inhibitors, particularly in DNA trapping and multi-targeting, suggests a promising avenue for future drug development.

In this work, we developed a PARP1-specific degradation PROTAC compound -180055, which connected to a Rucaparib junction at one end and a VHL ligand at the other. Absent the DNA trapping effect, 180055 demonstrated substantial PARP1 degradation in breast, ovarian, and other cancer cell lines. Moreover, 180055 demonstrates tumor-killing effects comparable to Rucaparib but with fewer side effects.

## Results

### Design and optimization of the structure of PARP1 degraders

**Fig. 1:**
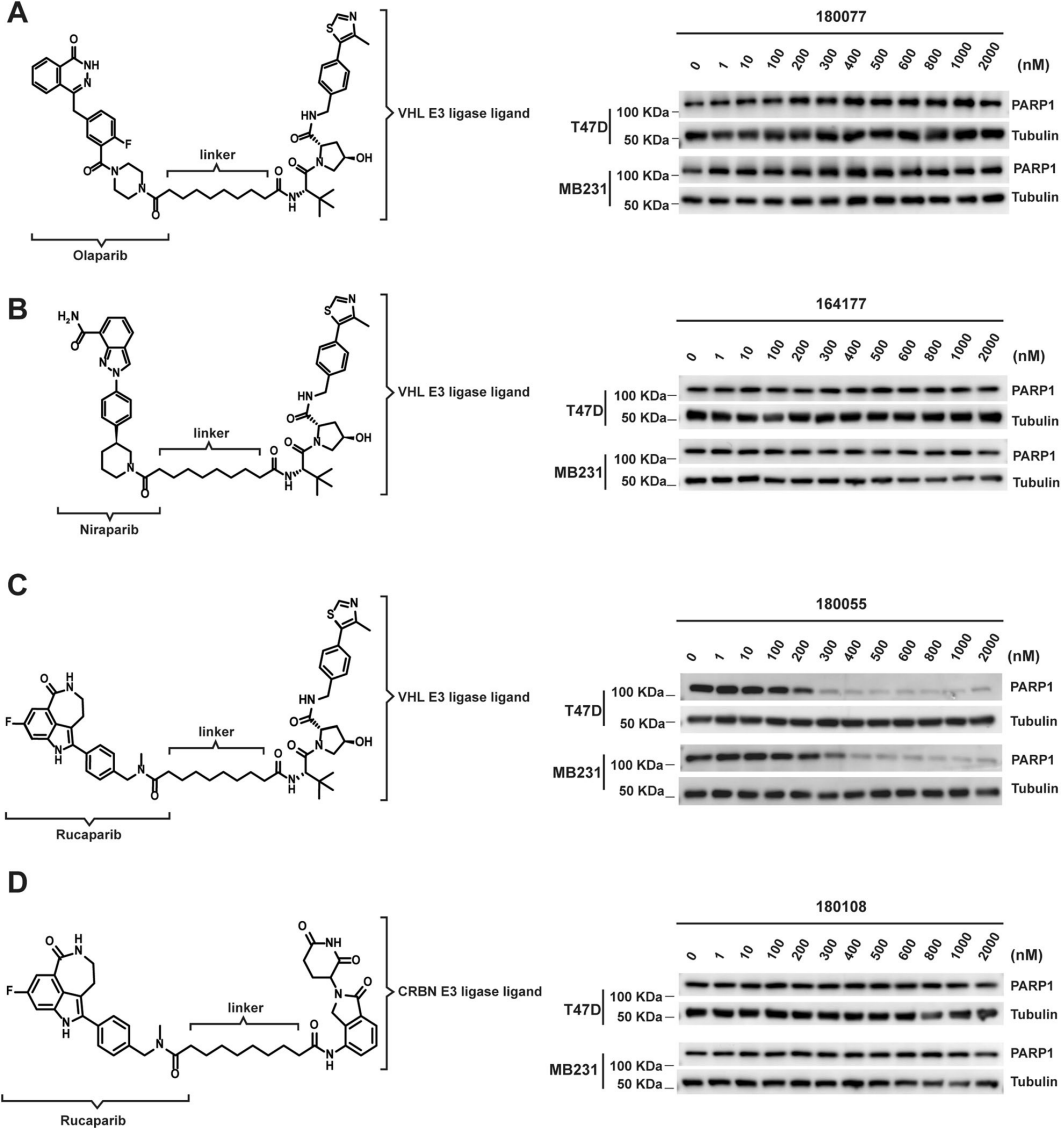
Significantly improved degradation effect due to the conjugation of VHL ligand to Rucaparib. **A**–**D** the structure of 180077 (**A**), 164177 (**B**), 180055 (**C**), and 180108 (**D**), and the expression of PARP1 was evaluated through western blot analysis in T47D and MDA-MB-231 cells after 24 h of treatment.

We endeavored to select three small molecule inhibitors, Olaparib, Niraparib, and Rucaparib, as the binders for PARP1 to synthesize its PROTAC degraders. The efficacy of these inhibitors in degrading PARP1 was assessed through western blotting analysis. Following a 24-h treatment with compounds 180077 and 164177, utilizing Olaparib and Niraparib as the PARP1 binder, respectively, minimal alteration was observed in the expression level of PARP1 in the cells. In contrast, treatment with compound 180055, which employs Rucaparib as the PARP1 binder, significantly reduced PARP1 expression within the cells (Fig. 1A–C). Compound 180108 was synthesized based on the structure of compound 180055, with the modification that the VHL E3 ligase ligand was replaced with the CRBN E3 ligase ligand. However, the results of western blotting analysis revealed that compound 180108 exhibited limited capability in causing the degradation of PARP1 protein (Fig. 1D).

**Fig. 2:**
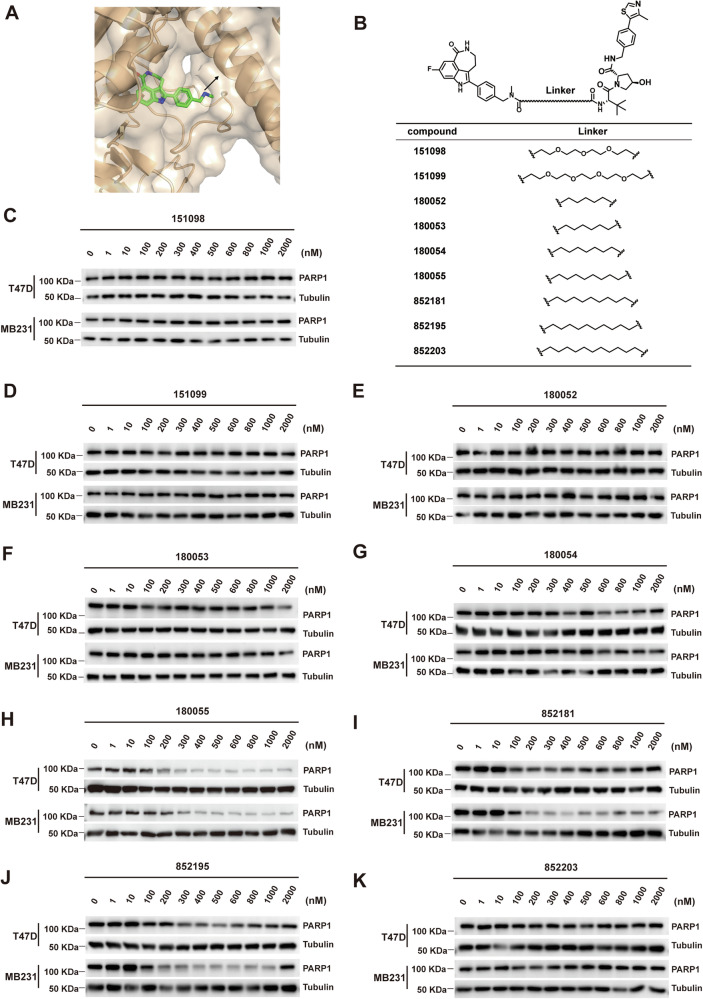
PARP1 degradation is affected by the lengths and types of the linker in the degrader. **A** Co-crystal structure of Rucaparib and PARP1 (PDB code: 4RV6). Arrows highlight the exit vector for linking. **B** Optimize the linker of the PROTAC compounds. **C**–**K** Immunoblot assays of PARP1 in T47D and MDA-MB-231 cells treated with PARP1 degraders bearing a (PEG)_3_ linker (**C**), (PEG)_4_ linker (**D**), five carbon atoms straight-chain alkyl linker (**E**), six carbon atoms straight-chain alkyl linker (**F**), seven carbon atoms straight-chain alkyl linker (**G**), eight carbon atoms straight-chain alkyl linker (**H**), nine carbon atoms straight-chain alkyl linker (**I**), ten carbon atoms straight-chain alkyl linker (**J**), 11 carbon atoms straight-chain alkyl linker (**K**) for 24 h.

The co-crystal structure of Rucaparib and PARP1 reveals that the secondary amine moiety of Rucaparib resides in a solvent-exposed region, indicating that derivatization at this site would not affect the binding of Rucaparib to PARP1 (Fig. 2A). Furthermore, this secondary amine can be directly tethered to a linker without the need for further structural modifications. Therefore, we connected linkers of varying lengths and types and VHL E3 ligase ligand to the secondary amine of Rucaparib and obtained a series of PARP PROTACs [28]. We designed a series of compounds with various lengths of straight-chain alkyl or PEG chains as linkers to bridge Rucaparib and VHL ligands (Fig. 2B). The degradation efficacy of each compound on PARP1 was evaluated in both T47D and MDA-MB-231 cell lines. Notably, compounds containing PEG linkers exhibited negligible levels of degradation (Fig. 2C, D). Among seven compounds with alkyl linkers of varying lengths, compound 180055, featuring a straight-chain alkyl linker comprising eight carbon atoms, demonstrated superior degradation of the PARP1 protein. Even a one-carbon difference in the linker length, as observed in compounds 180054 and 852181 compared to compound 180055, significantly attenuated their ability to induce PARP1 degradation (Fig. 2E–K).

### PROTAC 180055 facilitates the degradation of PARP1

**Fig. 3:**
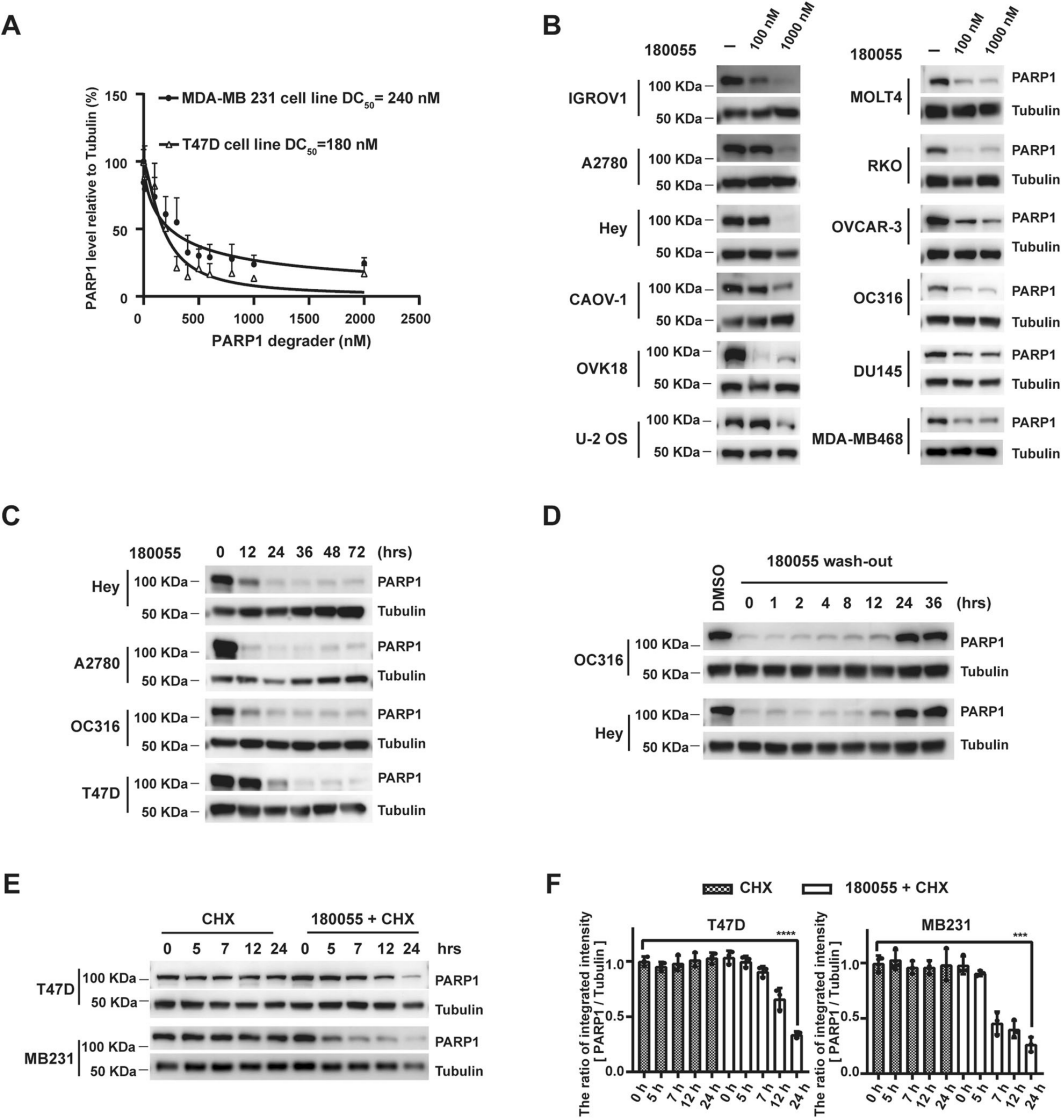
180055 induces PARP1 degradation. **A** Degradation curves of PARP1 treated with 180055 for 24 h in T47D and MDA-MB-231 cells. Data are mean ± s.e.m. from three biologically independent samples. **B** Cells were treated with increasing concentrations of 180055 for 24 h. **C** Cells were treated with 180055 (1 μM) for indicated times. **D** Cells were pre-treated with 180055 (1 μM) for 24 h. 180055 was then washed out for indicated times. **E** Cells were treated with cycloheximide (10 μg/ml; CHX) for the indicated times, together with or without 180055 treatment. Cell extracts were collected and immunoblotted with a PARP1 antibody, using Tubulin as a control. The result is representative of two biologically independent experiments. **F** Quantification of PARP1 bands was performed using ImageJ. Results are presented as mean ± SD from three replicates. ***p < 0.001, ****p < 0.0001.

The half-maximal degradation concentration (DC_50_) of 180055 for PARP1 protein in T47D and MDA-MB-231 cell lines was determined to be 180 nM and 240 nM, respectively (Fig. 3A). In addition, 180055 exhibited significant degradation potency towards PARP1 protein in 12 more cell lines, encompassing breast cancer cell lines (MDA-MB-468), ovarian cancer cell lines (IGROV1, A2780, Hey, CAOV1, OVK18, OC316, OVCAR3), colorectal cancer cell line (RKO), human acute T-lymphoblastic leukemia cell line (MOLT4), prostate cancer cell line (DU 145), as well as human osteosarcoma cell line (U-2 OS) (Fig. 3B).Subsequent treatment of cells with 180055 for varying durations revealed that PARP1 degradation commenced following a 12-h treatment with 180055, and this degradation effect persisted for up to 72 h (Fig. 3C). Moreover, when cells pre-treated with 180055 were subjected to washout, the expression of PARP1 protein was restored within a 24-h timeframe, indicating the reversible nature of PARP1 degradation mediated by 180055 (Fig. 3D). Simultaneous treatment of cells with 180055 and CHX elucidated that 180055 modulates the expression of PARP1 post-translationally, thereby reducing the half-life of PARP1 (Fig. 3E, F).

### The degradation of PARP1 induced by 180055 is dependent on the ubiquitin-proteasome system

**Fig. 4:**
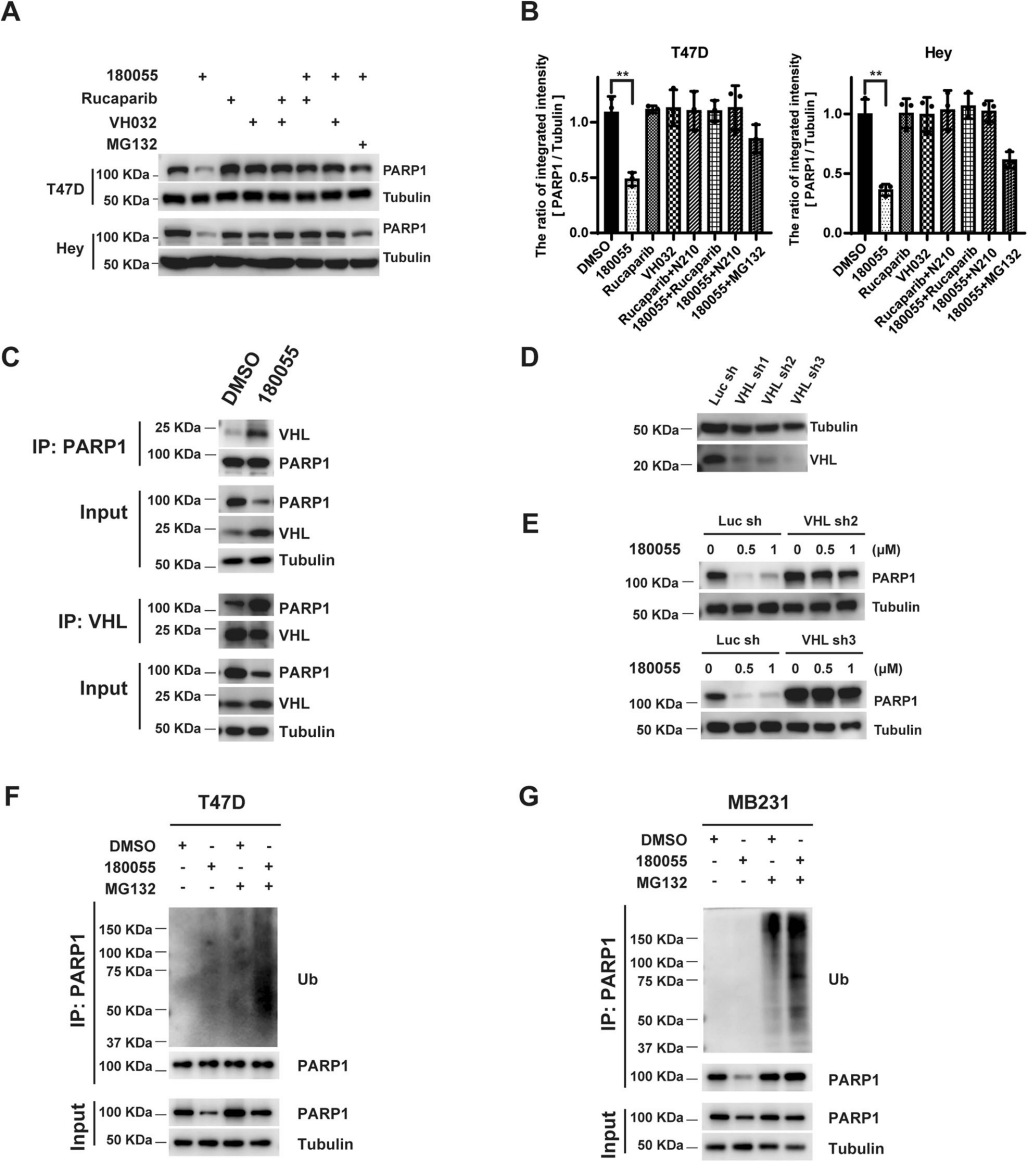
180055-induced PARP1 degradation is dependent on the ubiquitin-proteasome system. **A** Cells were treated with 180055 (1 μM), Rucaparib (1 μM), VHL ligand – VH032 (1 μM), Rucaparib (1 μM) plus VHL ligand – VH032 (1 μM), 180055 (1 μM) plus Rucaparib (1 μM), VHL ligand – VH032 (10 μM) or MG132 (1 μM) for 24 h. Cell extracts were harvested and subjected to immunoblotting analysis using an antibody specific to PARP1, with Tubulin as a control. **B** The intensities of PARP1 bands were quantified using ImageJ software. Data are represented as mean ± SD from three independent replicates. **p < 0.01. **C** T47D cells were treated with 180055 (1 μM) for 24 h. Co-immunoprecipitation (Co-IP) was carried out using a PARP1 or VHL antibody, followed by immunoblot analysis to detect PARP1 and VHL proteins. **D**, **E** PARP1 degradation depends on the presence of the VHL E3 ligase. **D** Western blot verification of VHL protein expression in MDA-MB-231 cells with different VHL shRNA infection or Luciferase shRNA as control. **E** MDA-MB-231 cells were treated with increasing concentrations of 180055 for 24 h. **F**, **G** 180055 induces ubiquitination of PARP1 in T47D (**F**) and MDA-MB-231 cells (**G**). Cells were treated with DMSO, 180055 (1 μM), DMSO plus MG132 (1 μM), or 180055 (1 μM) plus MG132 (1 μM) for 24 h. Immunoprecipitation was conducted using a PARP1 antibody and immunoblot analysis to detect ubiquitination.

Treatment of cells with either Rucaparib or VH032 (a VHL ligand [29, 30]) alone failed to induce the degradation of PARP1. Conversely, combined treatment with Rucaparib, VH032, and MG132 effectively inhibited the degradation of PARP1 induced by 180055 (Fig. 4A, B). These results strongly suggest that the degradation of PARP1 is primarily attributed to the action of 180055 itself rather than its individual components. Reciprocal co-immunoprecipitation assays further revealed that 180055 enhances the interaction between PARP1 and VHL at the endogenous level (Fig. 4C). Notably, the degradation of PARP1 induced by 180055 was significantly hindered in VHL-deficient cells with shRNA knockdown (Fig. 4D, E). Furthermore, the presence of 180055 promotes the ubiquitination of PARP1 in both T47D and MDA-MB-231 cells (Fig. 4F, G, Supplementary Fig. S1A, B). These findings suggest that 180055 facilitates the recruitment of PARP1 and VHL, subsequently triggering the ubiquitination and degradation of PARP1 via the ubiquitin-proteasome system.

### 180055 specifically induces the degradation of PARP1

**Fig. 5:**
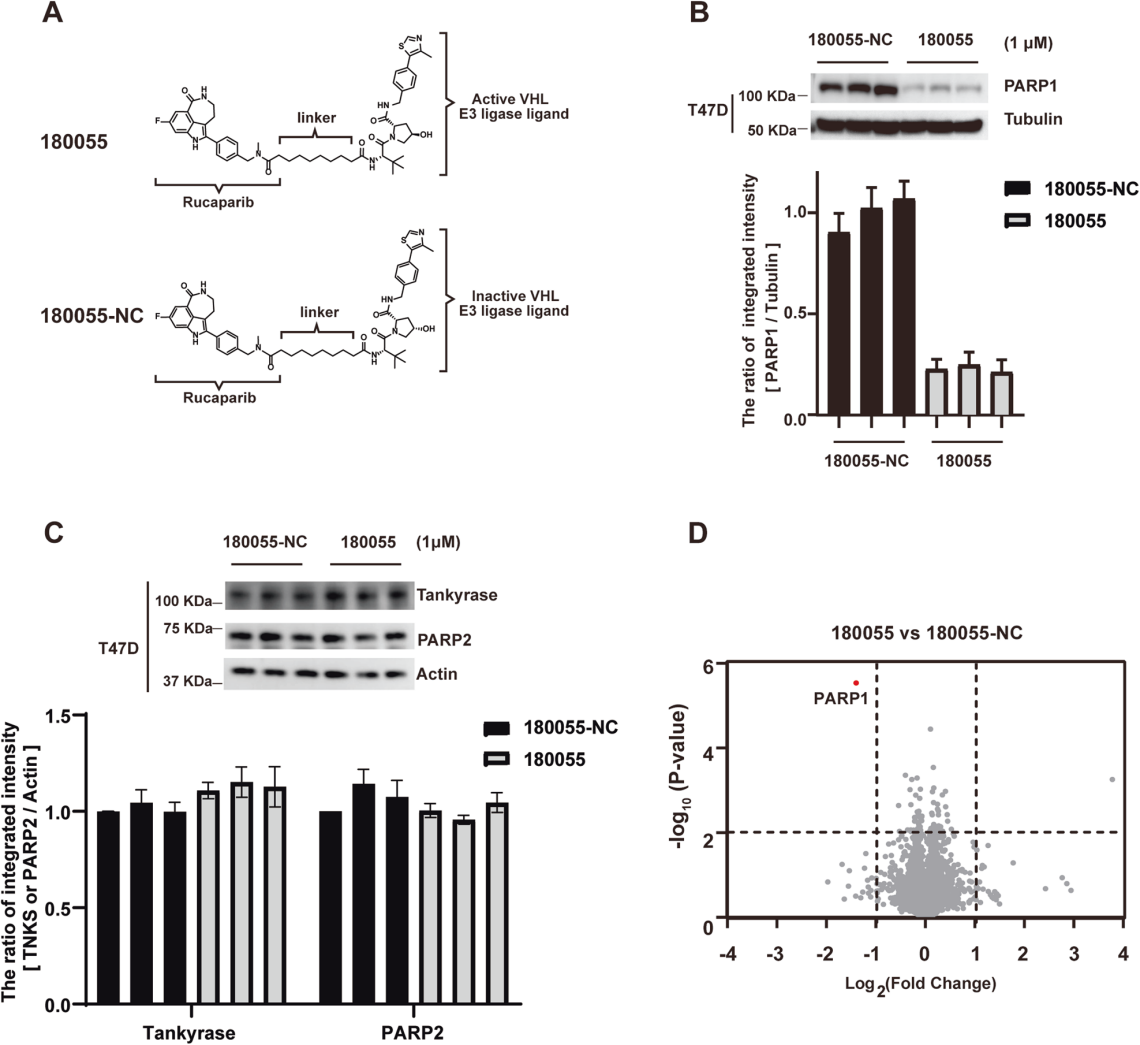
180055 selectively targets PARP1 for degradation. **A** Chemical structure of 180055 and 180055-NC. **B**, **C** Cells were treated with 180055 (1 μM) or 180055-NC (1 μM) for 24 h—western blot verification of PARP1 (**B**), PARP2 (**C**), and Tankyrase (**C**) protein expression. The result is representative of three biologically independent experiments. **D** MS-based quantitative proteomics analysis shows a specificity of 180055 for PARP1 degradation compared to 180055-NC in T47D cells. Each experiment had three biological replicates.

To evaluate the specificity of 180055 in degrading PARP1, we conducted a mass spectrometry-based quantitative proteomic analysis in the T47D cell line. To discern any potential off-target effects, we designed compound 180055-NC, which differs from 180055 solely in the configuration of the hydroxyl group within the VHL ligand moiety (Fig. 5A). Through a comparative analysis of proteomic profiles between cells treated with 180055 and 180055-NC, we observed a significant reduction in the expression of PARP1, without any detectable off-target effect elaborated by the results that there were no hits within the *p* < 0.01 and log_2_FC < −1 areas (Fig. 5B, D). Other members of the PARP family, Tankyrase and PARP2, were also not degraded following treatment with 180055 (Fig. 5C). These findings provide compelling evidence supporting the specificity of 180055 in targeting PARP1 for degradation.

### 180055 inhibits the catalytic activity of PARP1

**Fig. 6:**
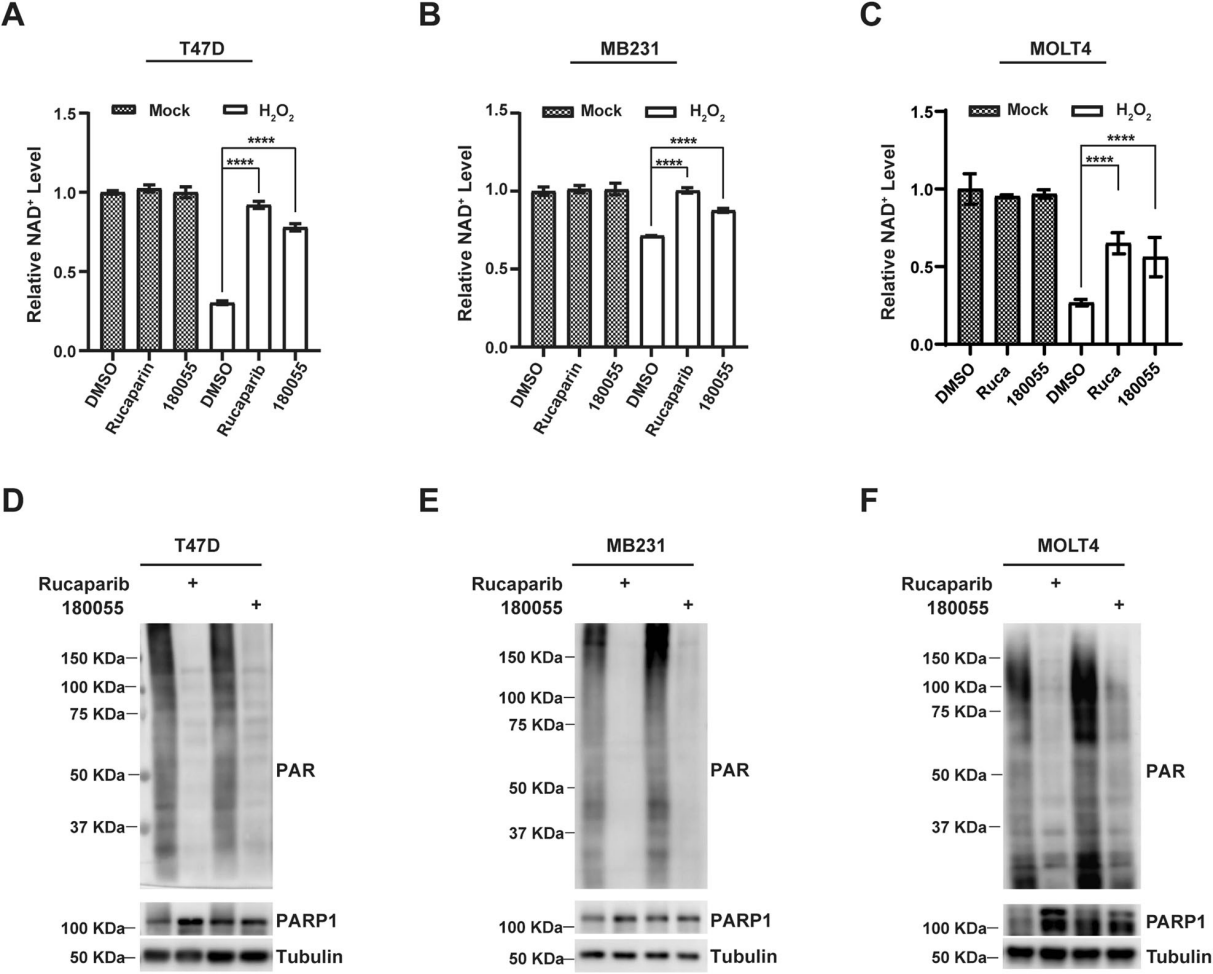
180055 blocks the catalytic activity of PARP1. **A**–**C** T47D (**A**), MDA-MB-231 (**B**), and MOLT4 cells (**C**) were pre-treated with Rucaparib or 180055 (1 μM) for 24 h and then challenged with H_2_O_2_ (2 mM) for 1 h. NAD^+^ levels were then determined; values represent mean ± SEM (*n* = 3 biological independent samples). Statistical significance was calculated with one-way ANOVA comparing the H_2_O_2_-treated DMSO group to the Rucaparib and 180055 groups. ***p < 0.001, ****p < 0.0001. **D**–**F** immunoblot analysis of PARylation levels in T47D (**D**), MDA-MB-231 (**E**), and MOLT4 cells (**F**) treated with either Rucaparib or 180055. Cells were pre-treated with a PARG inhibitor (PDD 00017273, 2 μM) for 1 h and then treated with Rucaparib or 180055 (1 μM) for 1 h. Cells were then challenged with H_2_O_2_ (2 mM) for 5 min, and whole cell lysates were analyzed using immunoblot assays with the indicated antibodies.

PARylation is a crucial enzymatic function of PARP1, closely associated with DNA damage repair processes [31]. Activation of PARP1 triggered by DNA damage leads to the consumption of NAD^+^ [32]. To investigate the impact of 180055 on the concentration of intracellular NAD^+^, cells were pre-treated with Rucaparib and 180055 and then exposed to H_2_O_2_ to induce DNA damage. Measurements revealed that both Rucaparib and 180055 effectively prevent NAD^+^ depletion (Fig. 6A–C). To further assess the impact of 180055 on PARP1 enzymatic activity, cells were pre-treated with PARG inhibitor (PDD 00017273) for one hour, followed by treatment with either Rucaparib or 180055 for an additional hour before exposure to 2 mM H_2_O_2_ for five minutes. Western blotting analysis demonstrated that both Rucaparib and 180055 displayed inhibitory effects on the synthesis of PARylated proteins (Fig. 6D–F). Consistent outcomes were obtained following a 24-h pretreatment with Rucaparib or 180055, followed by a one-hour treatment with PARG inhibitor (Supplementary Fig. S1C, D).

### 180055 protects cells from gene toxicity-induced cell death

**Fig. 7:**
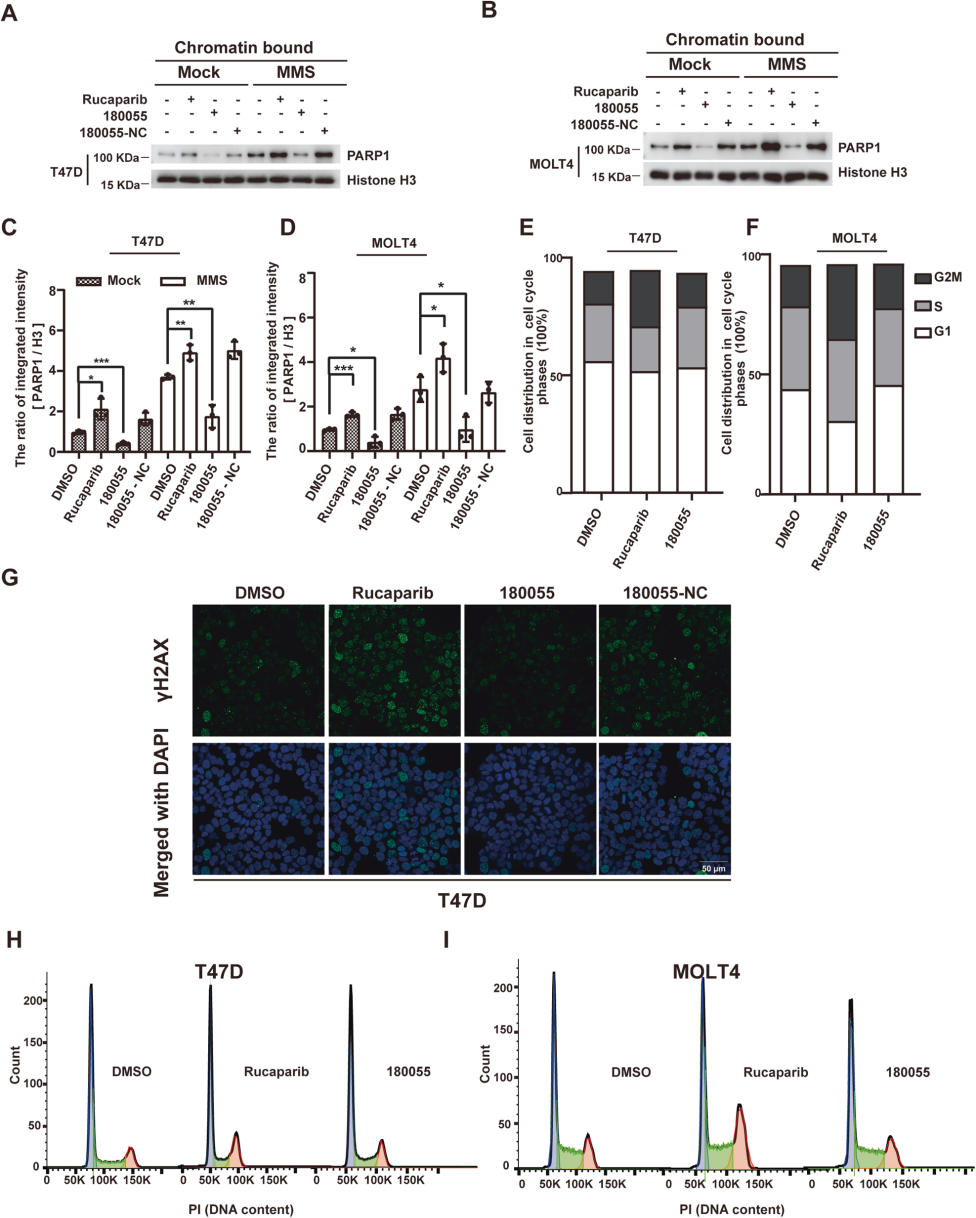
180055 protects cells from gene toxicity-induced cell death. **A**, **B**, DNA trapping of PARP1 in T47D (**A**) and MOLT4 cells (**B**) treated with Rucaparib, 180055 or 180055-NC. Cells were pre-treated with Rucaparib, 180055 or 180055-NC (1 μM) for 24 h followed by a 2-h treatment with DMSO (Mock) or MMS (0.01%). Chromatin-bound proteins were extracted and analyzed using the indicated antibodies. **C**, **D** PARP1 bands in T47D (**C**) and MOLT4 cells (**D**) were quantified with ImageJ. The result is representative of three biologically independent experiments. *p < 0.05, **p < 0.01, ***p < 0.001. **G** DNA damage induced by Rucaparib, 180055 or 180055-NC in T47D cells. Cells were treated with Rucaparib, 180055 or 180055-NC (10 μM) for 48 h, and DNA damage was detected by immunofluorescence staining of γH2AX. The result is representative of two biologically independent experiments. Scale bar, 50 μm. **H**, **I** Cell cycle analysis of T47D cells (**H**) and MOLT4 cells (**I**) after Rucaparib or 180055 treatment. T47D cells and MOLT4 cells were treated with Rucaparib or 180055 (10 μM) for 48 h and then were analyzed by flow cytometry. The left and right peaks indicate G1 and G2/M populations. **E**, **F** Quantification of data from (**H** and **I**).

To assess whether 180055 diminishes the chromatin trapping of PARP1 and subsequent DNA damage compared to Rucaparib, we initially treated T47D cells with Rucaparib, 180055, or 180055-NC. Subsequently, the cells were exposed to DMSO or methyl methanesulfonate (MMS), a reagent inducing alkylating DNA damage, followed by chromatin fractionation. Our findings revealed that 180055 significantly reduced PARP1 trapping on chromatin even upon MMS treatment, whereas 180055-NC displayed PARP1 trapping levels similar to Rucaparib due to its incapacity to degrade PARP1 (Fig. 7A, C). Similar results were observed in MOLT4 cells (Fig. 7B, D). Moreover, we examined DNA damage via immunofluorescence staining of γH2AX. As expected, 180055 induced less DNA damage than Rucaparib in T47D cells (Fig. 7G).

The cell cycle is a process of cell development whereby cells undergo continuous division to generate daughter cells. The G2 phase follows the DNA synthesis phase, where the cell readies itself for mitosis while its DNA content doubles. Cells in the G2 phase respond to DNA damage or incomplete replication by delaying the G2/M transition, preventing the segregation of impaired chromosomes. To discern potential discrepancies in the impact of Rucaparib and 180055 on the cell cycle, cells were treated with DMSO, Rucaparib, and 180055 compounds for 48 h, followed by cell staining and flow cytometry analysis to assess the cell cycle distribution. Our results revealed that Rucaparib induces more pronounced cell cycle arrest in the G2 phase than 180055 (Fig. 7E, F, H, I, Supplementary Fig. S1E–H).

### The anti-tumor effect of 180055 on the *BRCA1*-mutated cells in vitro and in vivo

**Fig. 8:**
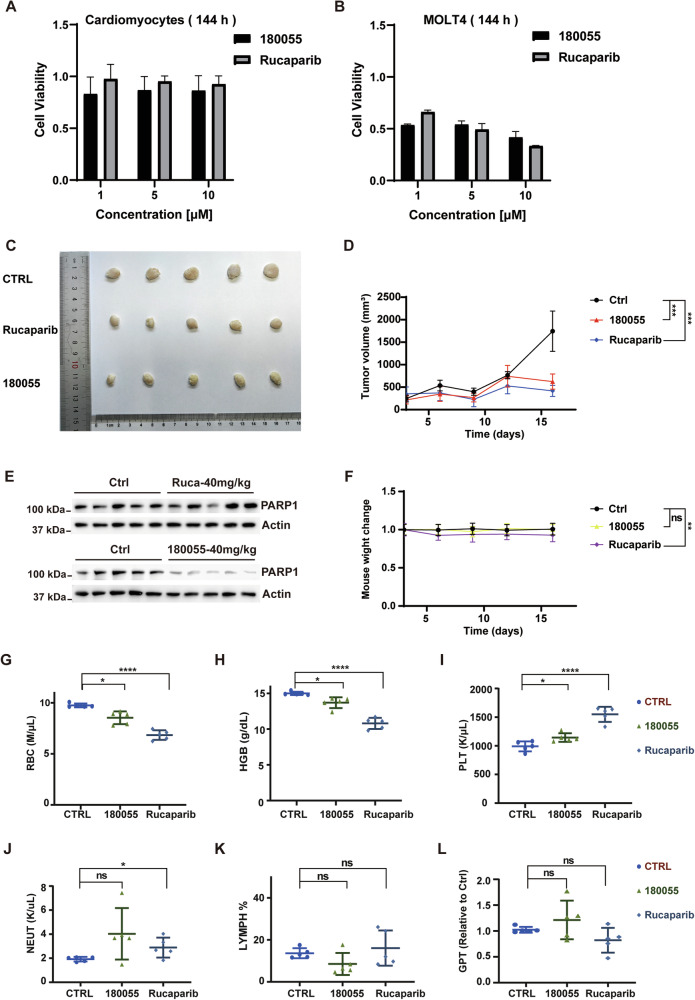
In vitro and in vivo evaluation of the anti-tumor efficacy of 180055 in *BRCA1*-mutated MOLT4 xenografts. **A**, **B** Cardiomyocytes (**A**), and MOLT4 cells (**B**) were subjected to treatment with Rucaparib or 180055 for 144 h. Cell viability was evaluated using the CTG luminescent cell viability assay. **C** Representative image of tumors from DMSO, 180055 or Rucaparib-treated group of mice (*n* = 5 per group). **D** Tumor growth curves of three groups of mice (*n* = 5 per group, mean ± SD). **E** Lysis of MOLT4 xenograft tumor cells was performed, followed by Western blot analysis to assess the expression levels of PARP1. **F** Average body weight changes the percentage of the mice in each group during 16 days of treatment (*n* = 5 per group, mean ± SD). The average weight of mice was normalized as the average weight of mice in the Ctrl group on the first day of administration as the standard. **G**–**L** Detection of Red Blood Cell Count (RBC), Hemoglobin (HGB), Platelet Count (PLT), Neutrophil (NEUT), Lymphocyte (LYMPH) percentage, and glutamic-pyruvic transaminase (GPT) content (*n* = 5 per group, mean ± SD). The data of 180055 or Rucaparib group and the Ctrl group were analyzed by *t*-test. *p < 0.05, **p < 0. 01, ***p < 0.001, ****p < 0.0001.

Synthetic lethality is a phenomenon where simultaneous alterations in two distinct genes result in cellular death [6, 7]. Certain cancers, including ovarian and breast cancers, among others, are particularly vulnerable to the effects of PARP inhibitors. This susceptibility stems from specific mutations in critical genes, such as those in the *BRCA* family, compromising alternative DNA repair pathways [33–35]. Considering the significant role of PARP1 in homologous recombination repair (HRR)-deficient cell lines [36], we exposed the cells to 180055 or Rucaparib for 3 and 6 days, then evaluated cell viability using the CTG assay. Minimal cytotoxicity of 180055 and Rucaparib was observed in cardiomyocyte cell lines lacking BRCA mutations (Fig. 8A, Supplementary Fig. S2A). In contrast, treatment with 10 μM of 180055 for 3 or 6 days induced substantial cytotoxic effects in MOLT4 cells with *BRCA1* mutation (Fig. 8B, Supplementary Fig. S2B).

Building upon the encouraging results from the CTG experiments, we evaluated the PROTAC compound’s anti-cancer efficacy in vivo. Immunodeficiency NSG mice harboring established subcutaneous MOLT4 tumor xenografts were treated with either Rucaparib (40 mg/kg) or 180055 (40 mg/kg) for 16 days. As depicted in Fig. 8C, D, both the Rucaparib and 180055 groups exhibited a noticeable reduction in tumor size compared to the control group. Notably, no significant weight loss was observed during the 16-day treatment with 180055, contrasting with the Rucaparib group, where weight loss was substantial (Fig. 8F). Western blot analyses demonstrated that 180055 effectively degraded PARP1 in tumors derived from MOLT4-xenografted mice (Fig. 8E). Furthermore, similar results were observed in another mouse model of A2780 tumor xenografts with BRCA mutations (Supplementary Fig. S2C–E).

Following euthanasia, we analyzed the blood profiles among all experimental mice. In mice treated with 40 mg/kg Rucaparib, we observed a significant decrease in erythrocyte count and hemoglobin levels and an increase in platelet counts (Fig. 8G–I). These changes were consistent with the reported effects for another PARP inhibitor, niraparib (30 mg/kg) [37]. Notably, Rucaparib treatment led to a slight elevation in neutrophil count, while no significant differences were noted in the 180055-treated group (Fig. 8J). No significant alterations in lymphocyte count or glutamic-pyruvic transaminase levels were noticed in the Rucaparib or 180055 treatment groups at the 40 mg/kg dosage (Fig. 8K, L). Combining together, our in vivo analysis revealed that 180055 exhibited minimal adverse effects, underscoring its favorable biological safety profile.

## Discussion

In 2018, the Rao group pioneered the development of a PROTAC molecule, designated as compound 3, which was shown to induce PARP1 cleavage and cell apoptosis in the MDA-MB-231 breast cancer cell line [38]. Concurrently, Yu’s research team identified iRucaparib-AP6, a PARP1 degrader demonstrating a DC_50_ of 82 nM in primary rat neonatal cardiomyocytes [39]. AP6 was found to protect muscle cells and primary cardiomyocytes from the energy crisis and cell death triggered by DNA damage, during which PARP1 is hyperactivated. However, it was noted that AP6 also degrades the PARP3 protein, which plays a crucial role in recruiting repair proteins and facilitating the end-linking complexes for DSB repair [40]. Furthermore, Chen’s group engineered a potent PARP1 degrader named SK-575, capable of effectively degrading PARP1 in various tumor cells at concentrations as low as picomolar levels. SK-575 was observed to induce a higher frequency of muscular double-strand breaks compared to Olaparib, a well-known PARP inhibitor, which may account for its enhanced in vivo tumoricidal efficacy [41]. Building upon these advancements, our study introduces a highly specific PROTAC compound, 180055, which is purposefully designed to inhibit PARP1. This compound effectively suppresses PARP1 enzymatic activity without the undesirable side effect of DNA trapping. Strikingly, 180055 exhibits tumoricidal capabilities that rival those of Rucaparib, yet with a significantly reduced side effect profile, marking a promising step forward in developing targeted cancer therapeutics.

In eukaryotic cells, proteasomes can be translocated through the nuclear membrane, shuttling rapidly between the nucleus and cytoplasm during cell division [42, 43]. Given the widespread presence of proteasomes in the nucleus, utilizing PROTACs for efficient degradation of nuclear proteins such as PARP1 holds significant development potential. The architecture of a PROTAC molecule is typically composed of three essential elements: ligands that bind to the target protein, ligands that interact with the E3 ubiquitin ligase, and a connecting linker. The design of these three components must be meticulously orchestrated to ensure the successful degradation of the protein of interest (POI). Among the various combinations of target protein ligands and E3 ligase ligands explored, the Rucaparib-based warhead in conjunction with the VHL ligase has emerged as the most effective duo for promoting degradation (Fig. 1). Notably, even a minor modification, such as the alteration of a single carbon atom within the linker, can dramatically impair the degradation efficiency of the PROTAC compounds (Fig. 2). This finding underscores the precision required in the spatial arrangement between the E3 ligase and the POI to facilitate the ubiquitination process effectively.

The mechanisms of action of PARP inhibitors are underpinned by two principal insights: the direct inhibition of PARP1’s enzymatic function and the subsequent trapping of PARP1 on chromosomes, the latter of which raises significant concerns regarding the side effects associated with clinically utilized PARP inhibitors. Our investigation introduces an approach with the PARP1 degrader 180055, which notably lacks an overt DNA trapping effect and cell cycle arrest (Fig. 7), beyond its role in inhibiting PARP1 activity (Fig. 6). The absence of DNA trapping with 180055 can be attributed to the Rucaparib moiety within its structure, which engages with the active site of PARP1, thereby curbing its catalytic activity. Following this initial interaction, 180055 facilitates the transfer of PARP1 to the ubiquitin-proteasome system (UPS) for subsequent degradation. This process precludes prolonged occupation of DNA damage sites by PARP1, effectively nullifying the DNA trapping effect. Beyond its synthetic lethality in BRCA-mutated tumors, 180055 also shows potential in treating acute and chronic non-neoplastic diseases (e.g., myocardial infarction, stroke, chronic inflammatory, and autoimmune diseases) [44]. The therapeutic effect stems from its ability to inhibit excessive activation of PARP1 triggered by genotoxic stress, which otherwise depletes NAD^+^ and reduces ATP, causing an energy crisis and cell death [45]. By blocking both enzymatic and non-enzymatic functions of PARP1, 180055 prevents NAD^+^ depletion, thus avoiding associated cell death.

A deficiency in PARP2 significantly disrupts the differentiation of erythroid lineage cells in mice, resulting in heightened hemolytic reactions and subsequent anemia. This perturbation triggers a cascade of complications, including ischemic and hypoxic changes in multiple organs, compromised immune function, and accelerated disease progression. Ultimately, this negatively influences patient prognosis and diminishes the quality of survival [20–22]. Given that both PARP1 and PARP2 possess highly conserved catalytic domains with NAD^+^ binding sites, designing small molecules specifically targeting PARP1 becomes more challenging. The current repertoire of PARP inhibitors, which includes Olaparib, Talazoparib, Rucaparib, Niraparib, Fluzoparib, Pamiparib, and Veliparib, is not entirely specific and tends to target both PARP1 and PARP2 [8–11, 19]. In contrast, our compound 180055 stands out for its lack of off-target effects. The mass spectrometry data, represented in a volcano plot with 5350 protein data points, reveals a striking specificity for PARP1, with only one target identified in the area where *P* < 0.01 and Log2 (Fold change) <−1 (Fig. 5). This pinpoints 180055 as a degrader with both high efficiency—achieving approximately 77% degradation—and unparalleled specificity, with PARP1 being the sole target of its action. Interestingly, analyzing from the perspective of atomic, although Rucaparib strongly binds to both PARP1 and PARP2 at similar binding sites, Rucaparib’s terminal amine forms two hydrogen bonds with PARP2’s S328 and G454 in both chains. However, in PARP1, no similar interaction occurs at G888 and G759 due to the larger distance (Supplementary Fig. S3) [46, 47]. This terminal amine group was not exposed any longer in the design of PROTAC molecules. Thus, although Rucaparib strongly binds to both PARP1 and PARP2, the modification of our compound 180055 at the terminal amine of the warhead Rucaparib prevents its binding to PARP2 while preserving the interaction with PARP1.

PARP inhibitors are known to induce synthetic lethality in cells with deficiencies in homologous recombination repair mechanisms, such as those harboring BRCA mutations [6, 7]. Leveraging the BRCA1-mutated MOLT4 cell line, our data confirmed that the compound 180055 is capable of significantly inhibiting tumor cell proliferation in both in vitro and in vivo models, corroborating the synthetic lethality principle. Concurrently, our xenograft studies detected a notable reduction in erythrocyte and hemoglobin levels in mice treated with 40 mg/kg Rucaparib, an effect potentially linked to PARP2 inhibition. The observed alterations in hemoglobin and platelet counts paralleled the effects of Niraparib (30 mg/kg), another PARP inhibitor, as documented in a 2021 study on mice [37]. Additionally, mice from the Rucaparib-treated cohort experienced a more pronounced body weight loss than the control group. Moreover, 180055 elicited a less pronounced effect on red blood cells, hemoglobin counts as well as platelet levels than Rucaparib. Significantly, the PARP1 PROTAC-treated group did not exhibit adverse effects on body weight, indicating superior tolerability to 180055 over Rucaparib at this dosage. While 180055 demonstrated a robust inhibitory impact on tumor growth, akin to Rucaparib, it did so with a markedly lower incidence of toxic side effects (Fig. 8).

Compound 180055, as a PARP1 degrader, may address acquired resistance in BRCA-mutated cancers, which is often linked to restored BRCA function or activation of alternative DNA repair pathways [9]. By inhibiting both enzymatic and non-enzymatic functions of PARP1, 180055 may help prevent these resistance mechanisms. Further research is needed to validate this hypothesis and assess its effects on resistance in BRCA-mutated cancers. Additionally, our study focused on evaluating the initial efficacy and toxicity of compound 180055, while highlighting the necessity for further research to better understand its long-term effects on both normal and cancerous tissues. Follow-up studies will explore potential resistance mechanisms that may arise with extended treatment.

Overall, this work presents a new design of the PARP1 PROTAC molecule, engineered based on the Rucaparib moiety and VHL ligand. Our comprehensive in vitro and in vivo studies have established that 180055 synergistically induces lethality in tumor cells harboring BRCA mutations while sparing normal cells from toxic effects. In conclusion, 180055 emerges as a promising clinical candidate with its remarkable ability to degrade PARP1 effectively and selectively, offering a potential new horizon for cancer therapeutics.

## Materials and methods

### Cell culture

MOLT4 (CRL-1582, ATCC), RKO (HTB-96, ATCC), DU 145 (HTB-81, ATCC), and HEK293T (CRL-3249, ATCC) were used for cell experiments. IGROV1, A2780, CAOV1, and OVK18 utilized in this research were procured from the study conducted by Gaofeng Fan et al. [48]. T47D, MDA-MB-231, and MDA-MB-468 were kindly provided by Xiaobao Yang’s team at Gluetacs Therapeutics (Shanghai). Hey, the cell was kindly provided by Prof Robert C Bast’s laboratory at MD Anderson Cancer Centre. U-2 OS was kindly provided by Prof Lei Li’s laboratory at ShanghaiTech University. OVCAR3 was kindly provided by Prof Robert Lucito’s lab in Cold Spring Harbor Laboratory. OC316 was kindly provided by Prof Ahmed Ashour Ahmed’s lab in the Ovarian Cancer Cell Laboratory, University of Oxford.

MDA-MB-231, IGROV1, A2780, Hey, CAOV1, OVK18, U-2 OS, RKO, OVCAR3, OC316, DU 145, MDA-MB-468, and HEK293T cells were cultured in DMEM medium supplemented with 10%FBS and 1% Pen/Strep at 37 °C with 5% CO_2_. T47D and MOLT4 were grown in RPMI 1640 with 10%FBS and 1% Pen/Strep at 37 °C with 5% CO_2_.

### Antibodies and reagents

The primary antibodies listed below were employed for western blot analysis and Co-immunoprecipitation: anti-GAPDH (Abclonal, cat. no. A19056); anti-PARP1 (Cell signaling technology, cat. no. 9532); anti-β-Tubulin (Cell signaling technology, cat. no. 2146); anti-VHL (Abmart, cat. no. MG496222S); anti-Ubiquitin (Cell signaling technology, cat. no. 3936); anti-PAR (R&D Systems, cat. no. 4335-MC-100); anti-Tankyrase (Proteintech, cat. no.18030-1-AP); anti-PARP2 (Proteintech, cat. no. 55149-1-AP); anti-γH2AX (Millipore, cat. no. 05-636); Alexa Fluor 647-conjugated anti-mouse secondary antibody (Thermo Fisher Scientific, cat. no. A21235).

Rucaparib (cat. no. S4948), MMS (cat. no. E0609), Cycloheximide (cat. no. S7418), MG132 (cat. no. S2619), and PDD 00017273 (cat. no. S8862) were purchased from Selleck. H_2_O_2_ (cat. no. H112517) was purchased from Aladdin.

### Immunoblotting

The cell extracts were prepared in a lysis buffer containing 1% NP40 (20 mM HEPES, 50 mM NaF, 150 mM NaCl, 10% glycerol, 1 mM Na3VO4, pH 7.5, and a mixture of Roche protease inhibitors) at 4 °C for 15 min. The BCA assay kit determined the total protein concentration (Beyotime, cat. no. P0010). Equal amounts of protein were subjected to SDS-PAGE gel electrophoresis, then transferred onto nitrocellulose membranes at 95 V and 4 °C for 90 min. The samples were blocked with a protein-free blocking solution (EpiZyme cat. no. PS108P) at room temperature for 15 min, followed by overnight incubation with the primary antibody at 4 °C. Subsequently, the samples were washed three times with TBST at room temperature and incubated with the secondary antibody conjugated with horseradish peroxidase (Cell Signaling Technology) at room temperature for 50 min. After washing three times with TBST, the membrane was developed using Super Signal West Pico PLUS chemiluminescent substrate (Thermo) and imaged on a GE developer (AI680UV). Full and uncropped western blots can be seen in Supplementary Materials

### Calculation of DC_50_

Cells were treated with an increasing concentration of PARP1 PROTAC for 24 h. Immunoblotting was performed to detect the expression of PARP1 and Tubulin, and ImageJ software was used for quantification. The expression of PARP was normalized by Tubulin expression. The baseline expression level of PARP1 in untreated cells (DMSO) was set at 100%. The expression level of PARP1 in treated cells was determined as a percentage relative to the baseline expression level in untreated cells. Values represent mean ± s.e.m. (*n* = 3).

### Cycloheximide treatment

T47D and MDA-MB-231 cells were evenly distributed into 12-well plates and cultured overnight to allow for adherence. Cells were treated separately with CHX (10 μg/ml) or co-treated with 180055 (1 μM) and CHX (10 μg/ml). At designated time points, the expression level of PARP1 was detected using immunoblotting.

### Immunoprecipitation and ubiquitination assays

T47D and MDA-MB-231 cells were untreated (DMSO), treated with 180055 (1 μM), DMSO plus MG132 (1 μM), or 180055 (1 μM) plus MG132 (1 μM) for 24 h. Cells were treated in a lysis buffer containing 1% NP40 (20 mM HEPES, 50 mM NaF, 150 mM NaCl, 10% glycerol, 1 mM Na3VO4, pH 7.5, and a mixture of Roche protease inhibitors) at 4 °C for 15 min. Centrifuge the cell lysate at 12,000 rpm for 15 min at 4 °C. The supernatant (1 mg) was incubated overnight at 4 °C with 1 μg of anti-PARP1 or anti-VHL antibodies. Protein A (GE/Amersham/Whatman, cat. no. 17127901) and G (GE/Amersham/Whatman, cat. no. 17061801) agarose beads were added and incubated for 3 h at 4 °C. The beads were washed three times with cell lysis buffer (20 mM HEPES pH 7.4, 150 mM NaCl, 1% NP40, 0.2% Triton X-100, and 5% Glycerol). The immunocomplexes were eluted from the beads by boiling at 95 °C for 10 min. The interaction between VHL and PARP1 proteins and the ubiquitination status of PARP1 was examined using Western blot analysis.

### NAD^+^ measurement

Cells were pre-treated with Rucaparib or 180055 for 24 h, followed by 2 mM H_2_O_2_ for 1 h. According to the manufacturer’s instructions, the intracellular NAD^+^ levels were determined using the NAD^+^/NADH assay kit (Beyotime, cat. no. S0175).

### CTG assay

The cytotoxic effects of 180055 on cells were assessed using the CellTiter-Glo (CTG) luminescent cell viability assay (Promega). Cells (1500 per well) were plated in a 96-well plate overnight and treated with increasing concentrations of 180055 for 3 or 6 days. Subsequently, CTG reagent was added to each well, mixing on a shaker for 5 min, and luminescence was measured using the SpectraMax i3 (MD).

### Knockdown assay

The shRNA sequences used in this work were provided in Supplementary Table 1, referred to https://www.sigmaaldrich.cn/CN/zh/semi-configurators/shrna?activeLink=productSearch. Three different shRNA fragments were packaged into the PLKO.1 vector (Addgene, # 10878). Lentivirus production was performed in HEK293T cells. Cells were infected with collected virus-containing supernatant, followed by puromycin selection, and survived cells were considered successfully infected with the shRNA virus after the selection. The knockdown efficiency of shRNA was verified using western blotting analysis.

### Quantitative mass spectrometry

We followed the procedure listed in Xiaotong Zhu et al. [49]. T47D cells were seeded in a 6-cm dish and treated with 1 μM 180055-NC or 1 μM 180055 for 24 h. After digestion, the cells were washed three times with PBS, and the protein lysate (50 mM NH_4_HCO_3_, 8 M urea (Thermo, cat. no. #75826) and protease inhibitor (Roche, cat. no. #05892791001)) were added to lysate the cells. Using an ultrasonic cell disruptor, cells were sonicated for 30 s with 5-s pulses and 2-s intervals at 32% energy until the tube bottom was free of visible pellets. Protein quantification of cell lysate was performed using a BCA protein assay kit (Beyotime, cat. no. #P0010). 100 μg of protein was taken, and 5 mM DTT (Sigma, cat. no. #D9163) was added to the protein solution. The mixture was then incubated at 37 °C for 1 h. Add 2-Iodoacetamide (IAM, Sigma, cat. no. #I1149) with a final concentration of 10 mM into the protein solution, place the EP tube in the dark for 45 min, add 50 mM ammonium bicarbonate (Sigma, cat. no. #A6141) into the protein solution, and dilute the urea concentration to 1 M. Add trypsin (Promega, cat. no. V5113) to the protein solution at a ratio of 1:50. Digest overnight at 37 °C. The next day, add 10% Trifluoroacetic acid (TFA, Fluka, cat. no. #14264) to the protein digest to achieve a final TFA concentration of 0.4% and test the pH to ensure it is between 2 and 4. Next, centrifuge the protein digest at 10,000 × *g* for 10 min and desalt the resulting peptide mixture using a desalting column (Sep-pak C18 kit). The peptide desalting product is finally washed into an EP tube and dried in a Speedvac. Finally, dissolve the peptide in 0.1% FA and load it onto an LC–MS/MS spectrometry instrument (Thermo Fisher, Q Exactive HF-X).

### Cell cycle analysis

Seed logarithmic phase cells into a 6-well plate and treated with DMSO, Rucaparib, and 180055 for 48 h. After drug treatment, wash the cells with pre-cooled PBS 2–3 times. Then, resuspend the cells in 1 ml of pre-cooled 75% ice-cold ethanol, fix them, and store them at 4 °C overnight. Next, resuspend the cells in PBS and add 100 μg/ml RNase A (Omega, cat. no. AC117-1) and 50 μg/ml Propidium iodide (Yeasen, cat. no. 40711ES10) to the cell suspension. Incubate in a light-protected oven for 30 min. After staining, wash the cells three times with PBS and analyze the cell cycle using a flow cytometer (BD LSRFortessa).

### Chromatin fractionation

Chromatin fractionation was carried out according to the procedure of Blessing et al., 2020 with some modifications [50]. In brief, 1.2 × 10^6^ cells were seeded in 6-well plates for the T47D cell line, and 2 × 10^6^ cells were seeded in the T25 flask for the MOLT4 cell line. 24 h after seeding, cells were treated with indicated drugs for 24 h and followed by 0.01% MMS treatment for 2 h. T47D cells were digested with a 0.25% trypsin-EDTA solution. Cells were collected by centrifugation at 500 × *g* for 3 min and washed once with cold PBS. Cells were subsequently pelleted and resuspended in 4× the pellet volume lysis buffer (30 mM Tris pH 7.5, 150 mM NaCl, 0.5% Triton X-100, 2 mM MgCl2 and Protease Inhibitor Cocktail (EDTA-Free)). Samples were incubated on ice for 15 min and centrifuged for 15 min at 15,000 × *g* at 4 °C to pellet the chromatin. The pellet was washed 3 times with lysis buffer. For each washing step, the sample was shortly vortexed and chromatin was pelleted for 5 min at 15,000 × *g* at 4 °C. The chromatin pellet was dissolved in a solution containing 8 mM NaOH and 1% SDS and then boiled at 95 °C for 15 min after the addition of 5× SDS-PAGE loading buffer. Samples were centrifuged for 15 min at 15,000 × *g* to collect the supernatant and subjected to the western blotting analysis.

### Immunofluorescence staining and imaging

For the T47D cell line, cells were grown on coverslips in 24-well plates and treated with indicated drugs for 48 h. Cells were washed once with PBS and fixed with 10% Neutral Formalin Fix Solution for 15 min at room temperature and washed three times with PBS. The cells were then permeabilized with 0.5% Triton X-100 in PBS for 10 min and blocked with 2% BSA in Cell Staining Buffer for 60 min. Fixed cells were incubated with a γH2AX (1:500) antibody at 4 °C overnight, washed three times with PBS, and incubated with an Alexa Fluor 647-conjugated anti-mouse secondary antibody (1:1000) at dark for 1 h at room temperature. Cells were then stained with 1 μg/ml DAPI for 10 min and washed three times with PBS for 5 min. Coverslips were mounted onto slides with a mounting medium. Images were collected on a Nikon CSU-W1 Sora Spinning Disk confocal microscope using a 60×/1.4 oil objective.

### Animals

Mice were housed in the SPF-level animal facility of the National Facility for Protein Science in Shanghai. All study protocols involving mice were approved by the Institutional Animal Care and Use Committee of ShanghaiTech University and conducted by governmental regulations of China for the care and use of animals.

### Xenograft experiment

Xenograft tumors were established by injecting 8 × 10^6^ MOLT4 cells or 4 × 10^6^ A2780 suspended in PBS (EpiZyme, CB012) subcutaneously on the dorsal side of 5-week-old NSG mice. When tumor volume reached approximately 50 mm^3^, the mice were selected based on tumor volume and randomly assigned to treatment and vehicle control groups with five animals in each group. Preparation of the treated compounds: 180055 and Rucaparib were dissolved in the vehicle containing 5% (v/v) DMSO, 10% (v/v) solution, and 85% (v/v) normal saline. The mice were categorized into three distinct groups based on the following treatments: blank control, 40 mg/kg 180055, and 40 mg/kg Rucaparib. Each group was subjected to intraperitoneal injections twice daily. Tumor size was measured using electronic calipers every two days during treatment. Body weights were measured every two days during the treatment period. Blood samples were collected from mice, and blood parameters such as RBC, HGB, PLT, NEU, and LYMPH were analyzed using the IDEXX ProCyte Dx Hematology Analyzer instrument and *P*roCyte Dx* Reagent kit (IDEXX, 98-71001-00). The blood samples were allowed to stand at room temperature for 1 h, followed by centrifugation at 3500 rpm for 10 min to obtain the supernatant. GPT was analyzed using the Glutamic-pyruvic Transaminase (GPT) Activity Assay Kit (Sangon Biotech, D799579-0050).

### Chemical synthesis

#### General methods

Reagents and solvents were purchased from commercial sources without further purification unless otherwise indicated. The progress of reactions was monitored by thin-layer chromatography (TLC) and LC–MS. The final compounds were purified by prepared HPLC. NMR spectra were obtained from a Bruker AVANCE III 500 MHZ (operating at 500 MHz for 1H) or an Ascend 400 MHz Bruker spectrometer (operating at 400 MHz for ^1^H NMR and 126 MHz for ^13^C NMR) and chemical shifts were reported in ppm relative to the residual CH_3_OD (3.31 ppm 1H) or *d*_*6*_-DMSO (2.50 ppm 1H) and coupling constants (J) are given in Hz. Multiplicities of signals are described as follows: s --- singlet, br. s --- broad singlet, d --- doublet, t --- triplet, m --- multiple. Mass spectra were recorded on a solan X 70 FT-MS spectrometer.

## Supplementary information

Supplementary Figure

Western blots

## Data Availability

The mass spectrometry proteomics data have been deposited to the ProteomeXchange Consortium (http://proteomecentral.proteomexchange.org) via the iProX partner repository [51, 52] with the dataset identifier PXD042311.
