# Supplementary material for: Minimizing DNA trapping while maintaining activity inhibition via selective PARP1 degrader
Source: Cell Death Dis. 2024 Dec 18;15(12):898. doi: 10.1038/s41419-024-07277-2 (PMC11655542; doi:10.1038/s41419-024-07277-2)
Supplement: Supplementary file 1 — Supplementary Figure [file 41419_2024_7277_MOESM1_ESM.docx]

**Supplemental information**

**Minimizing DNA trapping while maintaining activity inhibition via selective PARP1 degrader**

Li Chen^1, #^, Yahui Zou^1, #^, Renhong Sun^2, #^, Mei Huang^1^, Xiaotong Zhu^1^, Xiao Tang^1^, Xiaobao Yang^2,^ *, Dake Li^3,^ *, Gaofeng Fan^1,4,^* and Yu Wang^5,6,^*

^1^School of Life Science and Technology, ShanghaiTech University, Shanghai 201210, China; ^2^Gluetacs Therapeutics (Shanghai) Co, Ltd, Building 20, Lane 218, Haiji Road 6, Pudong District, Shanghai, 201306, China; ^3^Department of Gynecology, Nanjing Women and Children's Healthcare Hospital, Nanjing, 210000, China; ^4^Shanghai Clinical Research and Trial Center, Shanghai 201210, China; ^5^Department of Gynecology, Shanghai First Maternity and Infant Hospital, School of Medicine, Tongji University, Shanghai 200092, China; ^6^Shanghai Key Laboratory of Maternal Fetal Medicine, Shanghai Institute of Maternal-Fetal Medicine and Gynecologic Oncology, Clinical and Translational Research Center, Shanghai First Maternity and Infant Hospital, School of Medicine, Tongji University, Shanghai, 200092, China.

^#^Equal Contribution

*Correspondence:

Yu Wang, Department of Gynecology, Shanghai First Maternity and Infant Hospital, School of Medicine, Tongji University, Shanghai 200092, China; [renjiwangyu@126.com](mailto:renjiwangyu@126.com)

Gaofeng Fan, School of Life Science and Technology, ShanghaiTech University, Shanghai, 201210, China; fangf@shanghaitech.edu.cn. 86-13917147596.

Dake Li, Department of Gynecology, Nanjing Women and Children's Healthcare Hospital, Nanjing, 210000, China; lidake2002@163.com. 025-52226930

Xiaobao Yang, Gluetacs Therapeutics (Shanghai) Co., Ltd., Zhangjiang Hi-Tech Park, Shanghai, 201210, China; yang.xiaobao@gluetacs.com. 86-18964581118


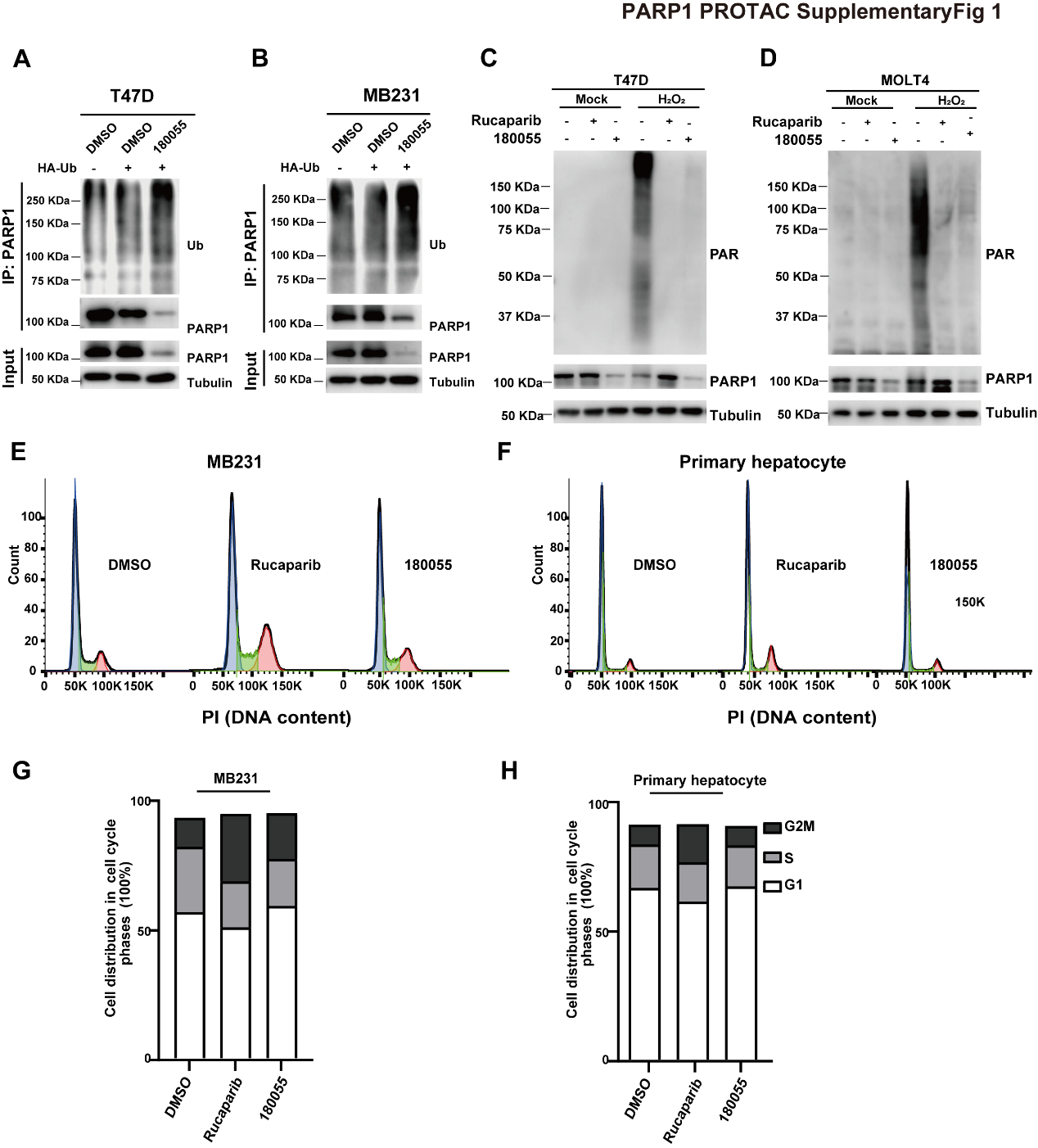


**Figure S1. Functional characterization of PARP1 PROTAC compound 180055 in normal and cancerous cells.**

**(A-B)** T47D (A) and MDA-MB-231 (B) cells were transfected with an HA-Ub plasmid for 24 hrs. Subsequently, the cells were treated with DMSO or 180055 (1 μM) for 24 hours. Co-immunoprecipitation (Co-IP) was then performed using a PARP1 antibody, followed by immunoblotting analysis to detect ubiquitination. **(C-D)** T47D (C) and MOLT4 (D) cells were pretreated with Rucaparib or 180055 (1 μM) for 24 hours, and treated with the PARG inhibitor PDD 00017273 (2 μM) for 1 hour. Then cells were treated with H_2_O_2_ (2 mM) for 5 minutes. Cell lysates were subjected by western blot analysis using the indicated antibodies. **(E-F)** Cell cycle analysis of MDA-MB-231 (E) and primary hepatocyte cells(F) after Rucaparib or 180055 treatment. MDA-MB-231 cells and primary hepatocyte cells were treated with Rucaparib or 180055 (10 μM) for 48 hours, and then were analyzed by flow cytometry. The left and right peaks indicate G1 and G2/M populations. **(G-H)** Quantification of data from E and F.

**
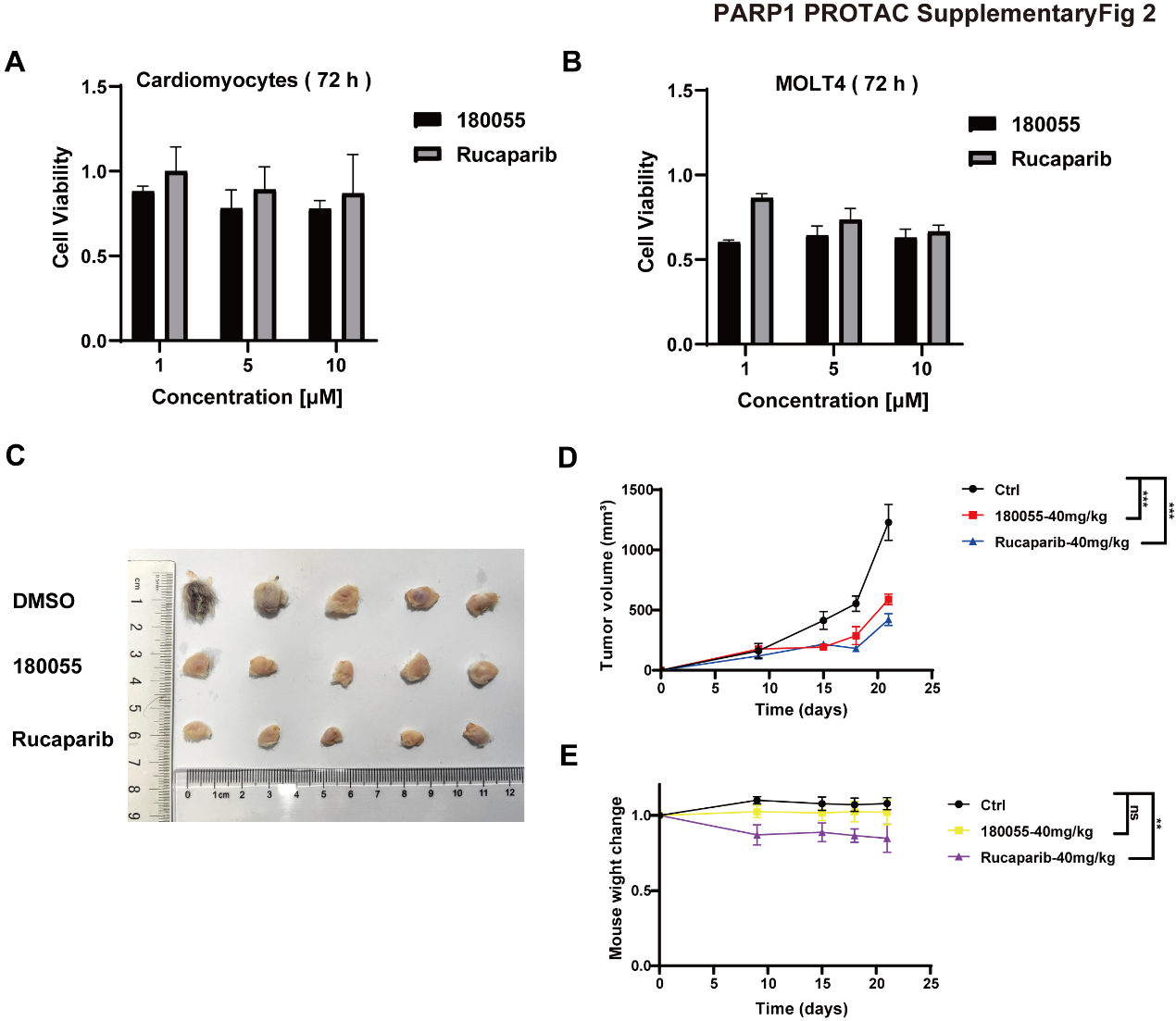
**

**Figure S2. Tumor inhibitor and blood indicators of PARP1 PROTAC compound 180055 in *vitro* and in *vivo*. (A-B)** Cardiomyocytes (A) and MOLT4 cells (B) were subjected to treatment with Rucaparib or 180055 for 72 hours. The evaluation of cell viability was conducted using the CTG luminescent cell viability assay. **C**, Representative image of tumors from DMSO, 180055 or Rucaparib-treated group of mice (n=5 per group). **D**, Tumor growth curves of mice treated with DMSO, 180055, or Rucaparib (n=5 per group, mean ± SD). **E**, Average body weight change percentage of the mice in each group during 16 days of treatment (n=5 per group, mean ± SD). The average weight of the mice was normalized to the average weight of the control group on the first day of treatment, which served as the standard reference. *p < 0.05, **p < 0.01, ***p < 0.001, ****p < 0.0001.


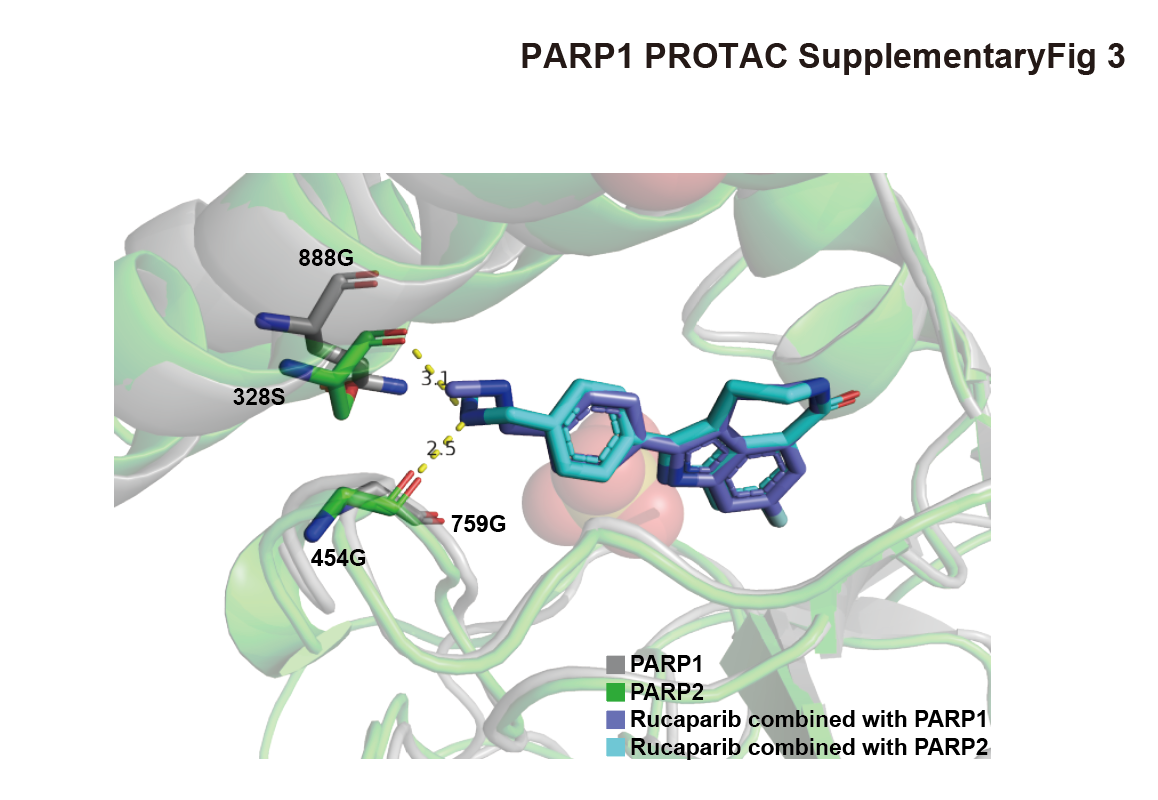


**Figure S3. A schematic representation illustrating the binding patterns of Rucaparib with both PARP1 and PARP2** **(Protein Data Bank [PDB] ID** **4RV6 and 8HKO).**

**Scheme S1. Synthesis of PARP PROTACs**

General procedure

Step 1: To a solution of VHL-1 (1.0 eq) and diacid (3 eq) in DMF (10 mL) was added EDCI (2 eq), HOBt (2 eq), and DIEA (3 eq). The reaction was stirred at room temperature for 12 h. LC-MS showed the reaction was completed. The reaction mixture was diluted with water (30 mL) and then extracted with EtOAc (30 mL) three times. The organic layers were combined, washed with water, dried over Na_2_SO_4_, and concentrated in a vacuum. The residue was purified by prep-HPLC (HCl condition) to give the desired product **Intermediate 1**; Step 2: To a solution of 8-fluoro-5-(4-((methylamino)methyl)phenyl)-2,3,4,6-tetrahydro-1H-azepino[5,4,3-cd]indol-1-one (1.5 eq) and **Intermediate 1** (1 eq) in DMF (10 mL) was added EDCI (2 eq), HOBt (2 eq) and DIEA (3 eq). The mixture was stirred at room temperature for 16 h. LC-MS showed the reaction was completed. The reaction mixture was diluted with water (50 mL) and then extracted with EtOAc (40 mL) three times. The organic layers were combined, washed with water, dried over Na_2_SO_4_, and concentrated in a vacuum. The residue was purified by prep-HPLC (HCl condition) to give the desired product **PARP PROTACs.**

(2*S*,4*R*)-1-((*S*)-17-(*tert*-butyl)-1-(4-(8-fluoro-1-oxo-2,3,4,6-tetrahydro-1*H*-azepino[5,4,3-*cd*]indol-5-yl)phenyl)-2-methyl-3,15-dioxo-6,9,12-trioxa-2,16-diazaoctadecan-18-oyl)-4-hydroxy-*N*-(4-(4-methylthiazol-5-yl)benzyl)pyrrolidine-2-carboxamide **151098**, white solid, yield: 75%). ^1^H NMR (500 MHz, CH_3_OD) *δ* 9.09 (d, *J* = 1.5 Hz, 1H), 7.64 – 7.57 (m, 2H), 7.50 (dt, *J* = 10.8, 2.3 Hz, 1H), 7.47 – 7.46 (m, 2H), 7.41 – 7.39 (m, 4H), 7.30 (dt, *J* = 9.0, 2.6 Hz, 1H), 4.74 – 4.67 (m, 2H), 4.64 – 4.63 (m, 1H), 4.58 – 4.48 (m, 3H), 4.35 – 4.32 (m, 1H), 3.88 (d, *J* = 11.0 Hz, 1H), 3.82 (t, *J* = 6.2 Hz, 1H), 3.79 – 3.77 (m, 2H), 3.71 – 3.65 (m, 2H), 3.62 – 3.52 (m, 10H), 3.13 – 3.11 (m, 2H), 3.07 (s, 2H), 2.97 (s, 1H), 2.76 – 2.71 (m, 2H), 2.56 – 2.51 (m, 1H), 2.49 – 2.41 (m, 4H), 2.23 – 2.19 (m, 1H), 2.09 – 2.04 (m, 1H), 1.01 (d, *J* = 4.2 Hz, 9H). HRMS (ESI) m/z: calcd for C_51_H_62_FN_7_NaO_9_S^+^ [M+Na]^+^, 990.4206; found, 990.4206.

*N*^1^-(4-(8-fluoro-1-oxo-2,3,4,6-tetrahydro-1*H*-azepino[5,4,3-*cd*]indol-5-yl)benzyl)-*N*^16^-((*S*)-1-((2*S*,4*R*)-4-hydroxy-2-((4-(4-methylthiazol-5-yl)benzyl)carbamoyl)pyrrolidin-1-yl)-3,3-dimethyl-1-oxobutan-2-yl)-*N*^1^-methyl-4,7,10,13-tetraoxahexadecanediamide **151099**, white solid, yield: 64%). ^1^H NMR (500 MHz, CH_3_OD) *δ* 9.09 (d, *J* = 2.0 Hz, 1H), 7.64 – 7.57 (m, 2H), 7.51 (dt, *J* = 10.8, 2.2 Hz, 1H), 7.48 – 7.45 (d, *J* = 8.1 Hz, 2H), 7.42 – 7.38 (m, 4H), 7.30 (dt, *J* = 9.0, 2.5 Hz, 1H), 4.73 – 4.67 (m, 2H), 4.63 (d, *J* = 3.0 Hz, 1H), 4.58 – 4.47 (m, 3H), 4.35 – 4.31 (m, 1H), 3.88 (d, *J* = 11.2 Hz, 1H), 3.82 (t, *J* = 6.2 Hz, 1H), 3.80 – 3.75 (m, 2H), 3.71 – 3.61 (m, 6H), 3.60 – 3.56 (m, 8H), 3.55 – 3.52 (m, 2H), 3.15 – 3.11 (m, 2H), 3.07 (s, 2H), 2.97 (s, 1H), 2.76 – 2.71 (m, 2H), 2.56 – 2.50 (m, 1H), 2.47 (s, 3H), 2.45 – 2.40 (m, 1H), 2.23 – 2.19 (m, 1H), 2.10 – 2.03 (m, 1H), 1.02 (d, *J* = 3.1 Hz, 9H). HRMS (ESI) m/z: calcd for C_53_H_66_FN_7_NaO_10_S^+^ [M+Na]^+^, 1034.4468; found, 1034.4461.

*N*^1^-(4-(8-fluoro-1-oxo-2,3,4,6-tetrahydro-1*H*-azepino[5,4,3-*cd*]indol-5-yl)benzyl)-*N*^7^-((*S*)-1-((2*S*,4*R*)-4-hydroxy-2-((4-(4-methylthiazol-5-yl)benzyl)carbamoyl)pyrrolidin-1-yl)-3,3-dimethyl-1-oxobutan-2-yl)-*N*^1^-methylheptanediamide **180052**, white solid, yield: 45%). ^1^H NMR (500 MHz, CH_3_OD) *δ* 9.97 (d, *J* = 3.5 Hz, 1H), 7.65 – 7.47 (m, 7H), 7.42 – 7.35 (m, 2H), 7.33 – 7.30 (m, 1H), 4.71 – 4.48 (m, 6H), 4.40 – 4.36 (m, 1H), 3.92 – 3.88 (m, 1H), 3.79 – 3.76 (m, 1H), 3.58 – 3.47 (m, 2H), 3.13 – 3.11 (m, 2H), 3.05 (s, 2H), 2.98 (s, 1H), 2.57 (s, 3H), 2.50 – 2.44 (m, 2H), 2.32 – 2.30 (m, 3H), 2.09 – 2.02 (m, 1H), 1.72 – 1.50 (m, 4H), 1.45 – 1.33 (m, 2H), 1.03 – 0.98 (m, 9H). HRMS (ESI) m/z: calcd for C_48_H_56_FN_7_NaO_6_S^+^ [M+Na]^+^, 900.3889; found, 900.3894.

*N*^1^-(4-(8-fluoro-1-oxo-2,3,4,6-tetrahydro-1*H*-azepino[5,4,3-*cd*]indol-5-yl)benzyl)-*N*^8^-((*S*)-1-((2*S*,4*R*)-4-hydroxy-2-((4-(4-methylthiazol-5-yl)benzyl)carbamoyl)pyrrolidin-1-yl)-3,3-dimethyl-1-oxobutan-2-yl)-*N*^1^-methyloctanediamide **180053**, white solid, yield: 48%). ^1^H NMR (500 MHz, CH_3_OD) *δ* 9.87 – 9.85 (m, 1H), 7.65 – 7.46 (m, 7H), 7.37 (dd, *J* = 14.2, 8.0 Hz, 2H), 7.32 – 7.29 (m, 1H), 4.71 – 4.48 (m, 6H), 4.39 – 4.36 (m, 1H), 3.90 (t, *J* = 11.5 Hz, 1H), 3.80 – 3.75 (m, 1H), 3.56 – 3.48 (m, 2H), 3.14 – 3.09 (m, 2H), 3.05 (s, 2H), 2.98 (s, 1H), 2.56 (d, *J* = 2.0 Hz, 3H), 2.50 – 2.43 (m, 2H), 2.33 – 2.19 (m, 3H), 2.09 – 2.04 (m, 1H), 1.69 – 1.56 (m, 4H), 1.45 – 1.26 (m, 4H), 1.03 – 0.99 (m, 9H). HRMS (ESI) m/z: calcd for C_49_H_59_FN_7_O_6_S^+^ [M+H]^+^, 892.4226; found, 892.4218.

*N*^1^-(4-(8-fluoro-1-oxo-2,3,4,6-tetrahydro-1*H*-azepino[5,4,3-*cd*]indol-5-yl)benzyl)-*N*^9^-((*S*)-1-((2*S*,4*R*)-4-hydroxy-2-((4-(4-methylthiazol-5-yl)benzyl)carbamoyl)pyrrolidin-1-yl)-3,3-dimethyl-1-oxobutan-2-yl)-*N*^1^-methylnonanediamide **180054**, white solid, yield: 49%). ^1^H NMR (500 MHz, CH_3_OD) *δ* 9.90 – 9.87 (m, 1H), 7.65 – 7.46 (m, 7H), 7.39 – 7.35 (m, 2H), 7.32 – 7.30 (m,1H), 4.71 – 4.48 (m, 6H), 4.40 – 4.35 (m, 1H), 3.90 (d, *J* = 11.2 Hz, 1H), 3.79 – 3.76 (m, 1H), 3.57 – 3.47 (m, 2H), 3.13 – 3.11 (m, 2H), 3.05 (s, 2H), 2.99 (s, 1H), 2.56 (d, *J* = 6.4 Hz, 3H), 2.50 – 2.43 (m, 2H), 2.32 – 2.19 (m, 3H), 2.08 – 2.03 (m, 1H), 1.69 – 1.57 (m, 4H), 1.38 – 1.29 (m, 6H), 1.01 (d, *J* = 5.5 Hz, 9H). HRMS (ESI) m/z: calcd for C_50_H_61_FN_7_O_6_S^+^ [M+H]^+^, 906.4383; found, 906.4383.

*N*^1^-(4-(8-fluoro-1-oxo-2,3,4,6-tetrahydro-1*H*-azepino[5,4,3-*cd*]indol-5-yl)benzyl)-*N*^10^-((*S*)-1-((2*S*,4*R*)-4-hydroxy-2-((4-(4-methylthiazol-5-yl)benzyl)carbamoyl)pyrrolidin-1-yl)-3,3-dimethyl-1-oxobutan-2-yl)-*N*^1^-methyldecanediamide **180055**, white solid, yield: 72%). ^1^H NMR (400 MHz, *d_6_*-DMSO) δ 11.68 (d, *J* = 10.2 Hz, 1H), 8.98 (s, 1H), 8.56 (t, *J* = 6.1 Hz, 1H), 8.28-8.19 (mz, 1H), 7.85 – 7.81 (m, 1H), 7.62 (dd, *J* = 20.6, 8.2 Hz, 2H), 7.44 – 7.30 (m, 8H), 4.63 (s, 1H), 4.55-4.52 (m, 2H), 4.45-4.40 (m, 2H), 4.34 (s, 1H), 4.24-4.18 (m, 1H), 3.69-3.62 (m, 2H), 3.30 – 3.27 (m, 2H), 3.03 (s, 2H), 2.95 (s, 2H), 2.84 (s, 1H), 2.44 (s, 3H), 2.39 – 2.32 (m, 2H), 2.28-2.22 (m, 1H), 2.13 – 2.00 (m, 2H), 1.94-1.85 (m, 1H), 1.59-1.40 (m, 4H), 1.35 – 1.17 (m, 8H), 0.92 (d, *J* = 6.3 Hz, 9H). ^13^C NMR (126 MHz, *d_6_*-DMSO) δ 172.85, 172.62, 172.45, 170.20, 168.91, 159.79, 157.92, 152.24, 147.62, 140.13, 138.12, 137.73, 137.18, 135.67, 131.94, 130.83, 129.84, 129.11, 128.65, 128.42, 128.28, 127.92, 127.32, 126.29, 123.65, 112.18, 112.11, 110.12, 109.91, 101.20, 100.99, 69.34, 59.17, 56.82, 56.77, 52.67, 50.16, 42.33, 42.12, 38.42, 35.67, 35.33, 34.04, 32.94, 32.61, 29.21, 26.84, 25.92, 25.38, 25.14, 16.16. HRMS (ESI) m/z: calcd for C_51_H_62_FN_7_NaO_6_S^+^ [M+Na]^+^, 942.4359; found 942.4367.

*N*^1^-(4-(8-fluoro-1-oxo-2,3,4,6-tetrahydro-1*H*-azepino[5,4,3-*cd*]indol-5-yl)benzyl)-*N*^11^-((*S*)-1-((2*S*,4*R*)-4-hydroxy-2-((4-(4-methylthiazol-5-yl)benzyl)carbamoyl)pyrrolidin-1-yl)-3,3-dimethyl-1-oxobutan-2-yl)-*N*^1^-methylundecanediamide **852181**, white solid, yield: 30%). ^1^H NMR (400 MHz, *d_6_*-DMSO) δ 11.69 (d, *J* = 9.9 Hz, 1H), 8.99 (s, 1H), 8.55 (t, *J* = 6.0 Hz, 1H), 8.27-8.20 (m, 1H), 7.84-7.80 (m, 1H), 7.65-7.58 (m, 2H), 7.44 – 7.43 (m, 8H), 4.63 (s, 1H), 4.55-4.53 (m, 2H), 4.45-4.40 (m, 2H), 4.34 (s, 1H), 4.22 (dd, *J* = 15.9, 5.4 Hz, 1H), 3.68-3.61 (m, 2H), 3.39-3.34 (m, 2H), 3.03 (s, 2H), 2.95 (s, 2H), 2.84 (s, 1H), 2.44 (s, 3H), 2.39 – 2.32 (m, 2H), 2.27-2.22 (m, 1H), 2.12-2.00 (m, 2H), 1.93-1.86 (m, 1H), 1.58 – 1.38 (m, 4H), 1.33-1.16 (m, 10H), 0.93 (s, 9H). HRMS (ESI) m/z: calcd for C_52_H_64_FN_7_NaO_6_S^+^ [M+Na]^+^, 956.4515; found, 956.4524.

*N*^1^-(4-(8-fluoro-1-oxo-2,3,4,6-tetrahydro-1*H*-azepino[5,4,3-*cd*]indol-5-yl)benzyl)-*N*^12^-((*S*)-1-((2*S*,4*R*)-4-hydroxy-2-((4-(4-methylthiazol-5-yl)benzyl)carbamoyl)pyrrolidin-1-yl)-3,3-dimethyl-1-oxobutan-2-yl)-*N*^1^-methyldodecanediamide **852195**, white solid, yield: 38%). ^1^H NMR (400 MHz, *d_6_*-DMSO) δ 11.68 (d, *J* = 9.4 Hz, 1H), 8.98 (s, 1H), 8.55 (t, *J* = 6.0 Hz, 1H), 8.28-8.19 (m, 1H), 7.83-7.81 (m, 1H), 7.65 (d, *J* = 8.0 Hz, 1H), 7.59 (d, *J* = 8.0 Hz, 1H), 7.42-7.31 (m, 8H), 4.63 (s, 1H), 4.55-4.53 (m, 2H), 4.45-4.40 (m, 2H), 4.35 (s, 1H), 4.24-4.18 (m, 1H), 3.70-3.58 (m, 2H), 3.29-3.26 (m, 2H), 3.03 (s, 2H), 2.95 (s, 2H), 2.85 (s, 1H), 2.44 (s, 3H), 2.39-2.32 (m, 2H), 2.27-2.22 (m, 1H), 2.11-2.00 (m, 2H), 1.94-1.84 (m, 1H), 1.58-1.39 (m, 4H), 1.31-1.16 (m, 12H), 0.93 (s, 9H). HRMS (ESI) m/z: calcd for C_53_H_66_FN_7_NaO_6_S^+^ [M+Na]^+^, 970.4672; found, 970.4668.

*N*^1^-(4-(8-fluoro-1-oxo-2,3,4,6-tetrahydro-1*H*-azepino[5,4,3-*cd*]indol-5-yl)benzyl)-*N*^13^-((*S*)-1-((2*S*,4*R*)-4-hydroxy-2-((4-(4-methylthiazol-5-yl)benzyl)carbamoyl)pyrrolidin-1-yl)-3,3-dimethyl-1-oxobutan-2-yl)-*N*^1^-methyltridecanediamide **852203**, white solid, yield: 45%). ^1^H NMR (400 MHz, *d_6_*-DMSO) δ 11.68 (d, *J* = 9.4 Hz, 1H), 8.98 (s, 1H), 8.55 (t, *J* = 6.0 Hz, 1H), 8.28-8.19 (m, 1H), 7.83-7.81 (m, 1H), 7.65-7.58 (m, 2H), 7.42-7.31 (m, 8H), 4.63 (s, 1H), 4.55-4.52 (m, 2H), 4.45-4.40 (m, 2H), 4.34 (s, 1H), 4.24-4.18 (m, 1H), 3.70-3.61 (m, 2H), 3.26-3.21 (m, 2H), 3.03 (s, 2H), 2.95 (s, 2H), 2.85 (s, 1H), 2.44 (s, 3H), 2.39-2.32 (m, 2H), 2.29-2.19 (m, 1H), 2.13-1.98 (m, 2H), 1.94-1.86 (m, 1H), 1.58-1.39 (m, 4H), 1.32-1.19 (m, 14H), 0.93 (s, 9H). HRMS (ESI) m/z: calcd for C_54_H_68_FN_7_O_6_S^+^ [M+Na]^+^, 984.4828; found, 984.4834.

**Data S1: NMR spectra related to the chemical synthesis and characterization**

^1^H NMR or ^13^C NMR spectrum of PARP1 PROTACs


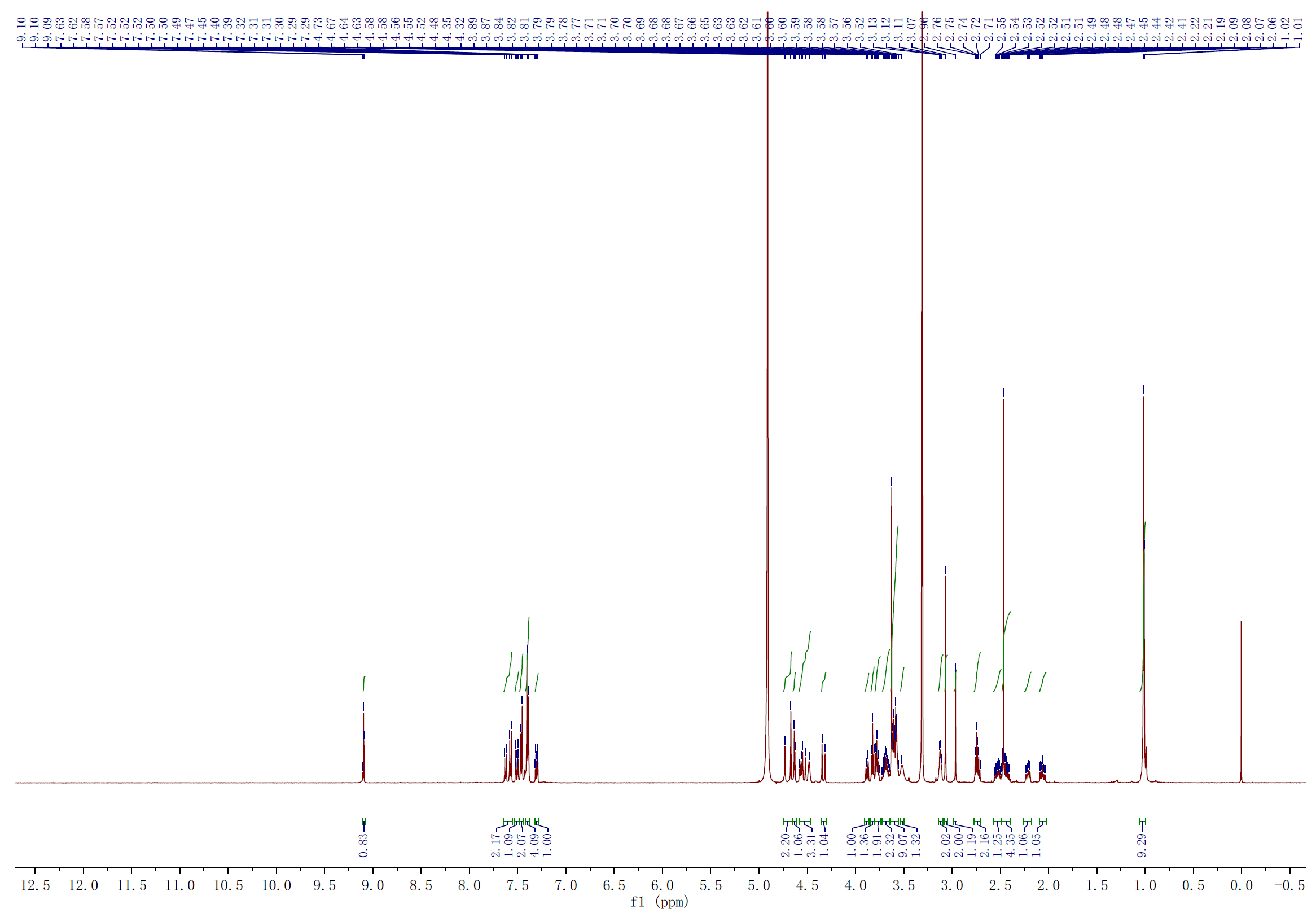


**151098**


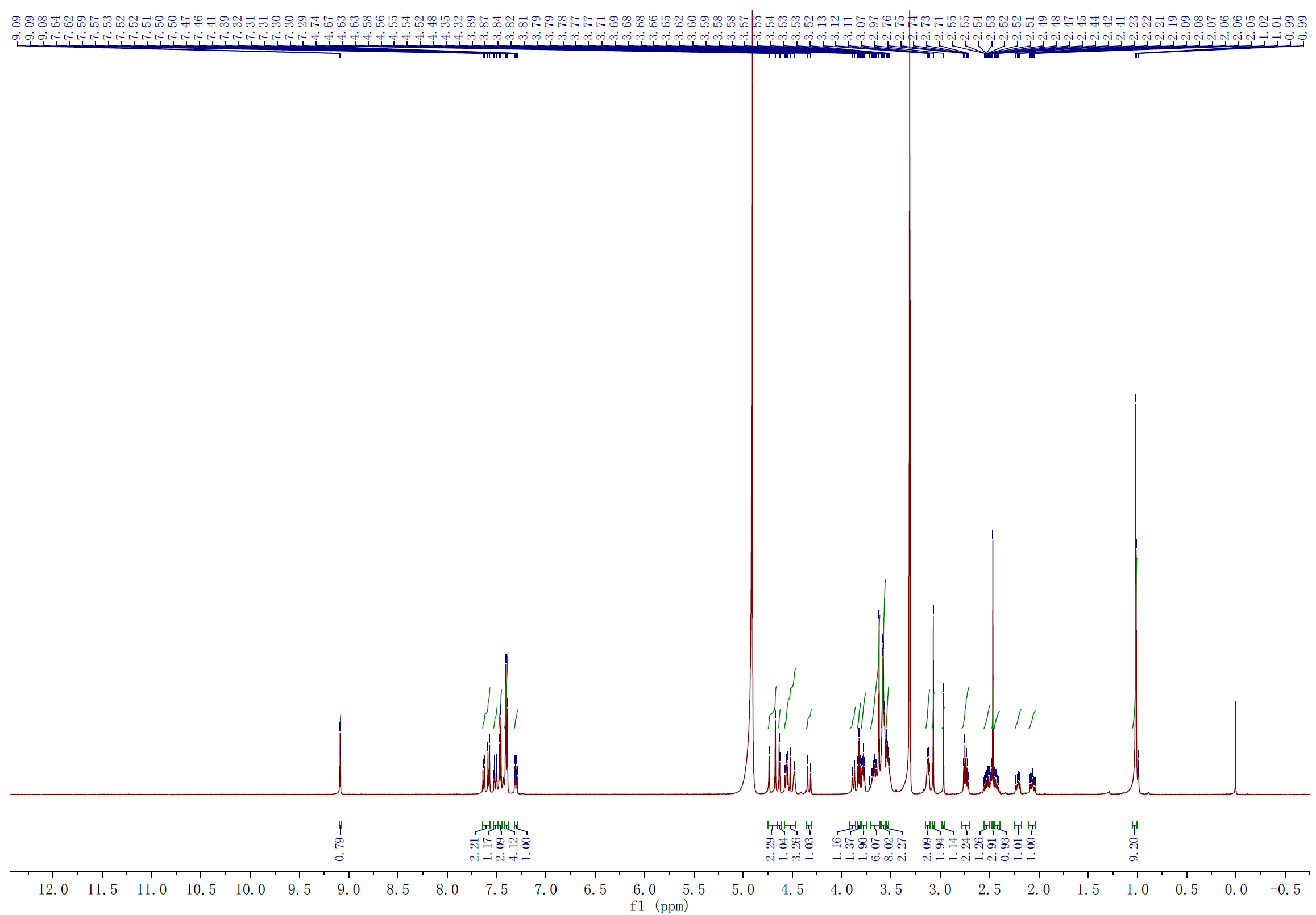


**151099**


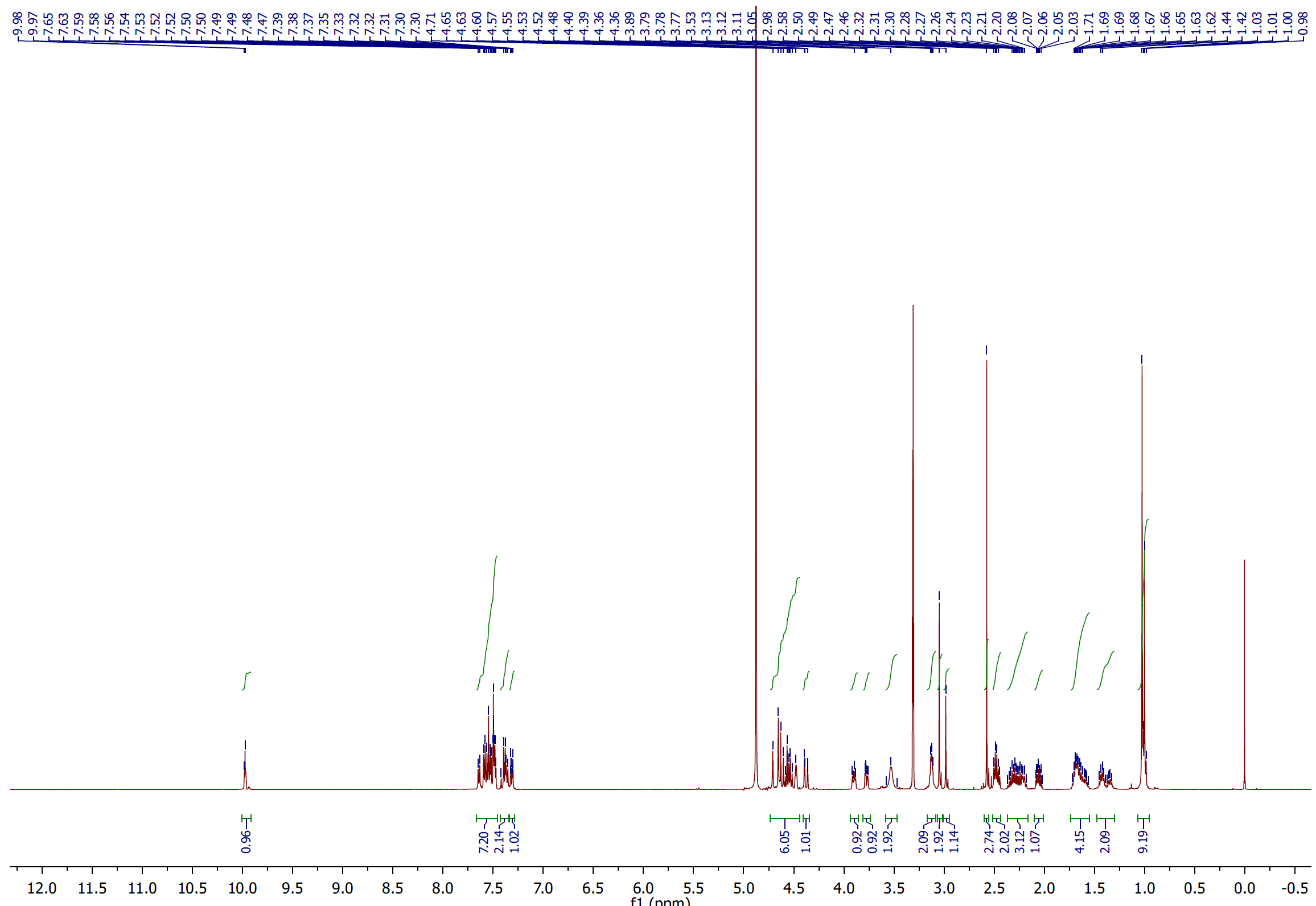


**180052**


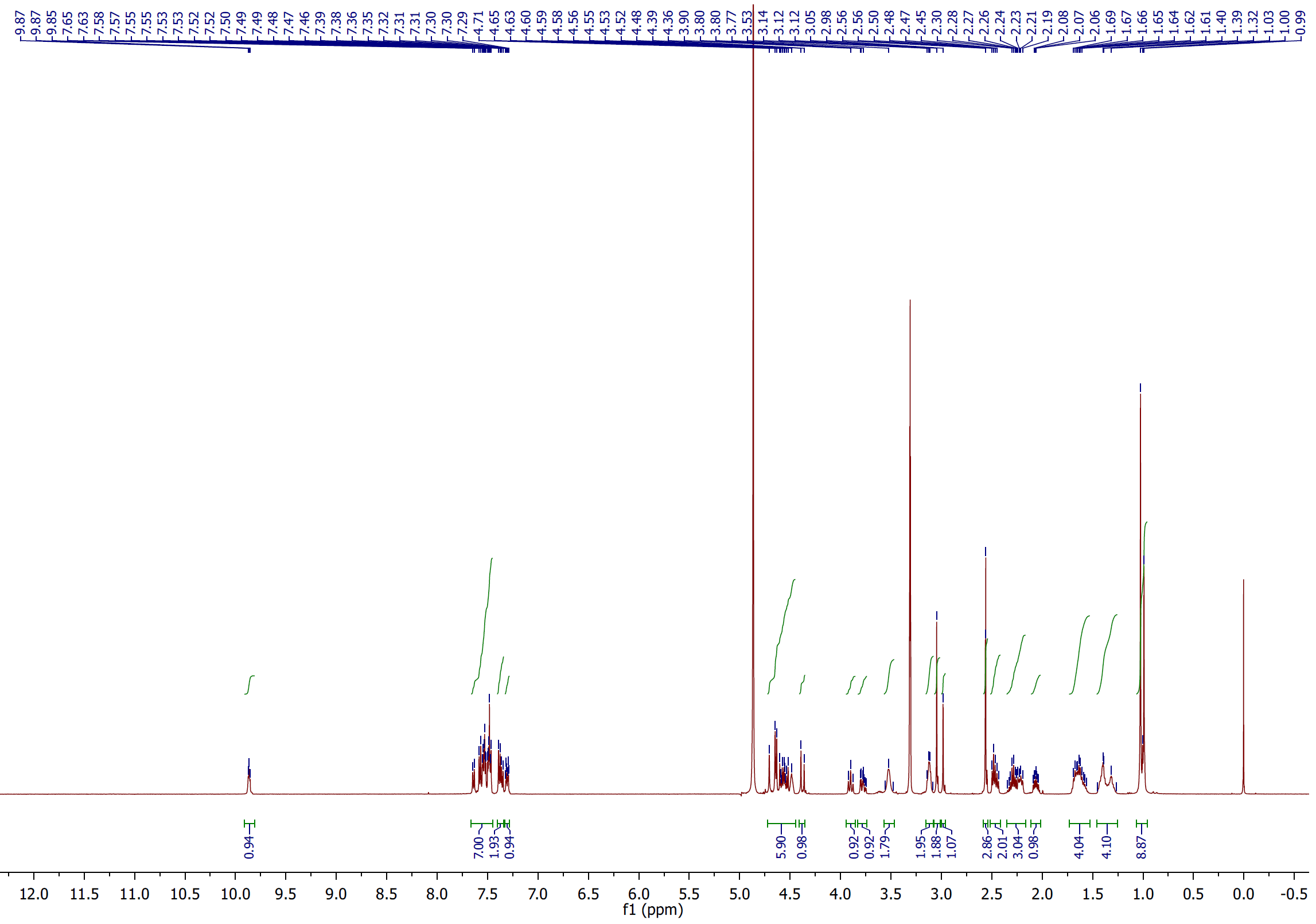


**180053**


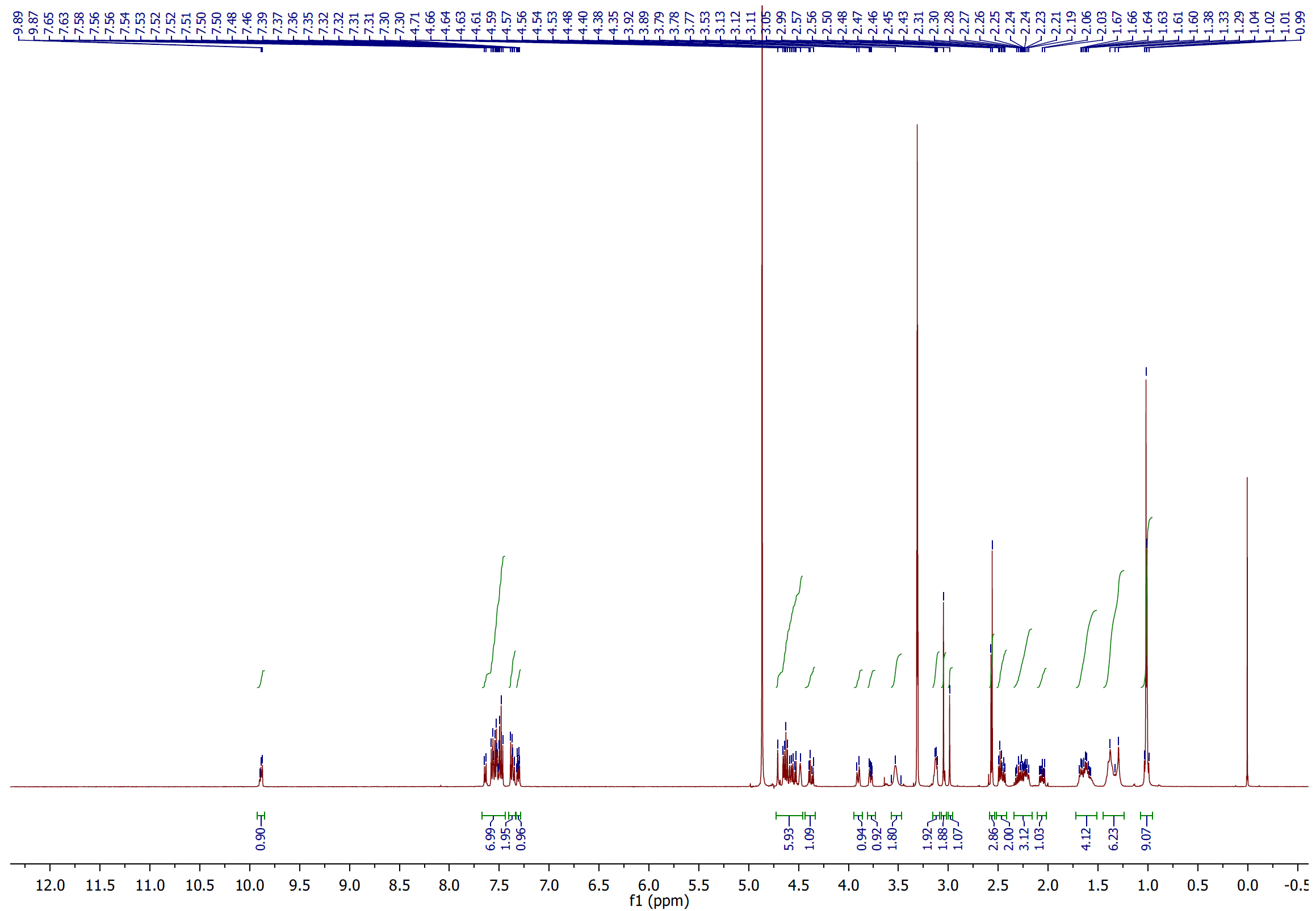


**180054**


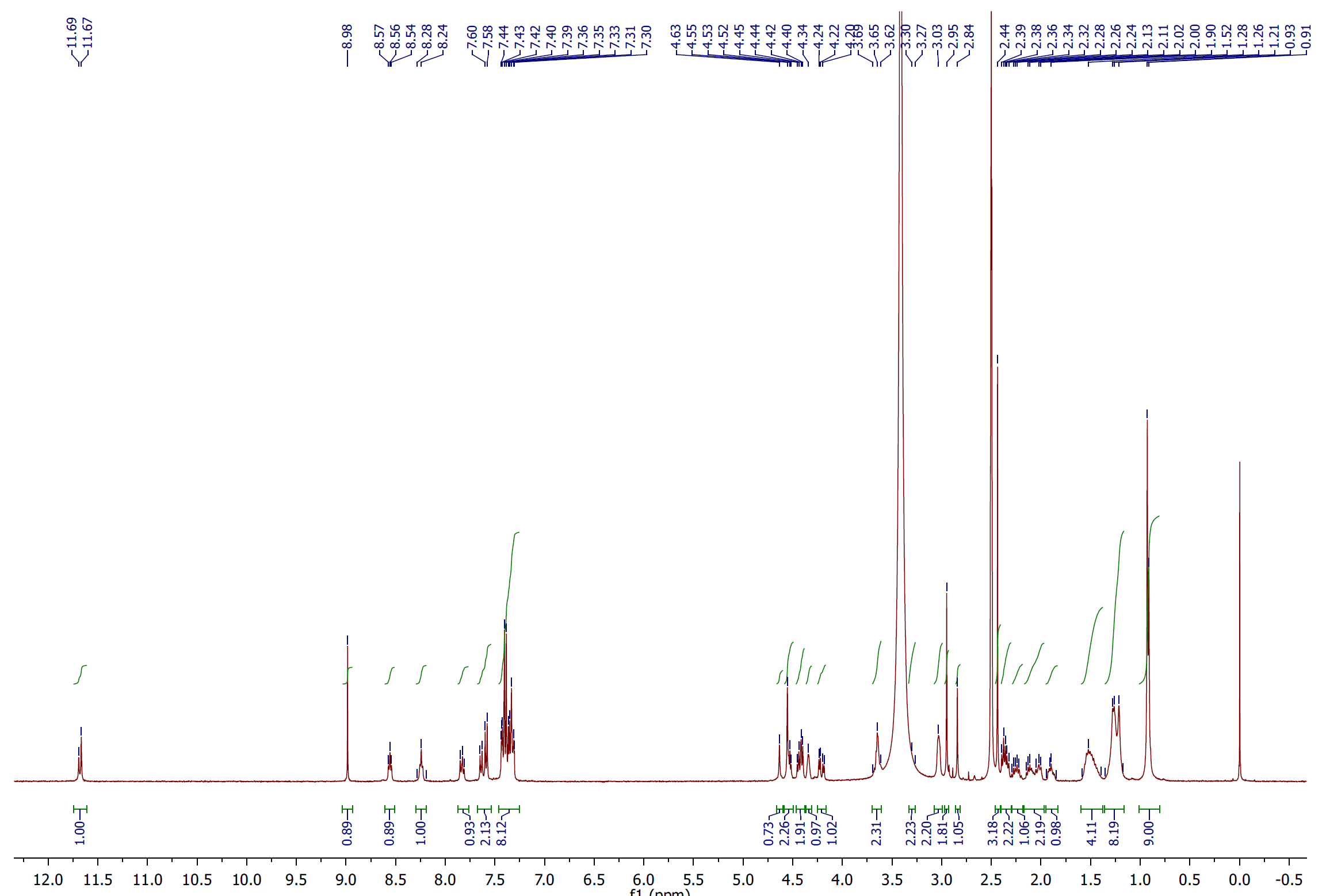


**180055**


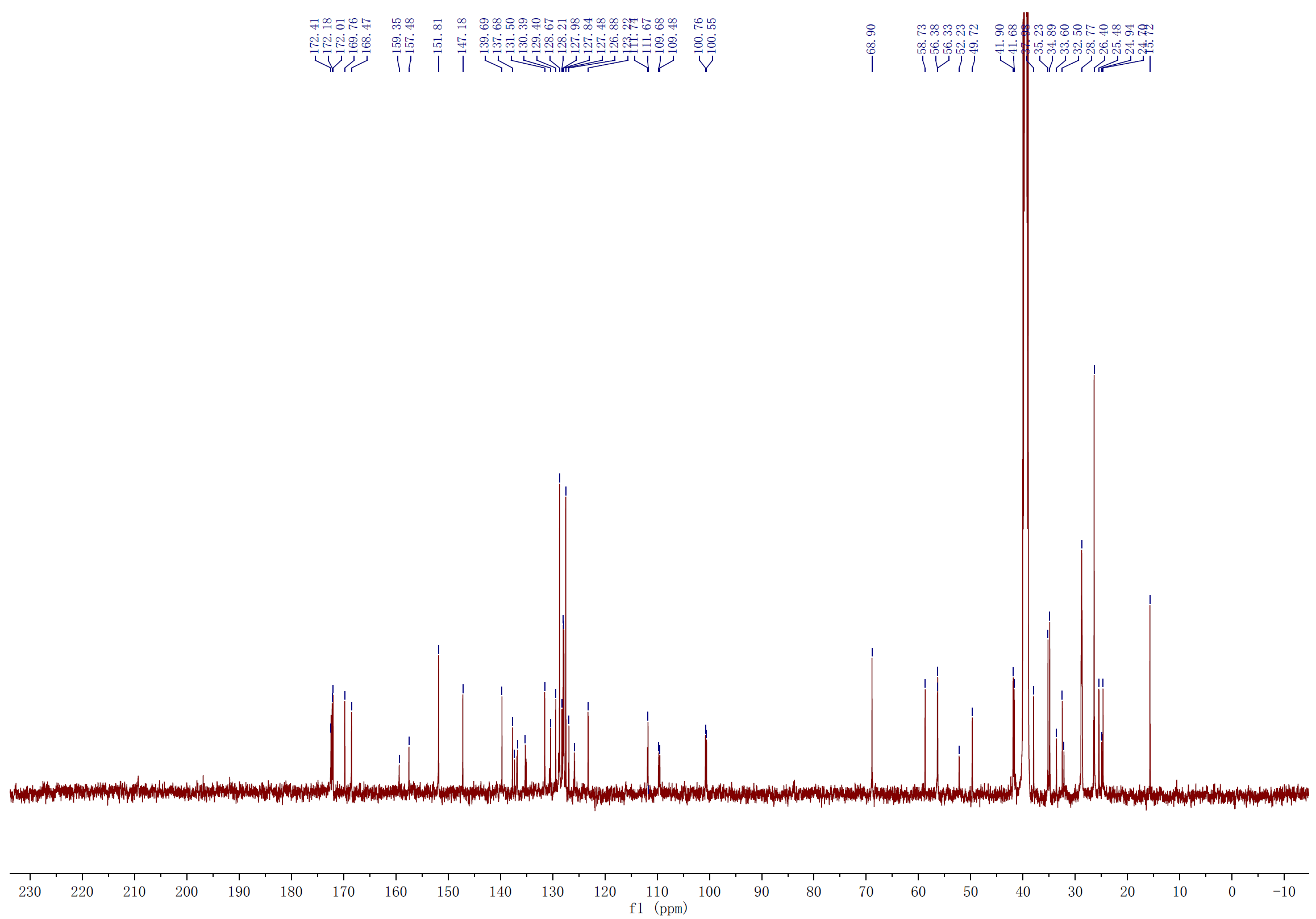


**180055**


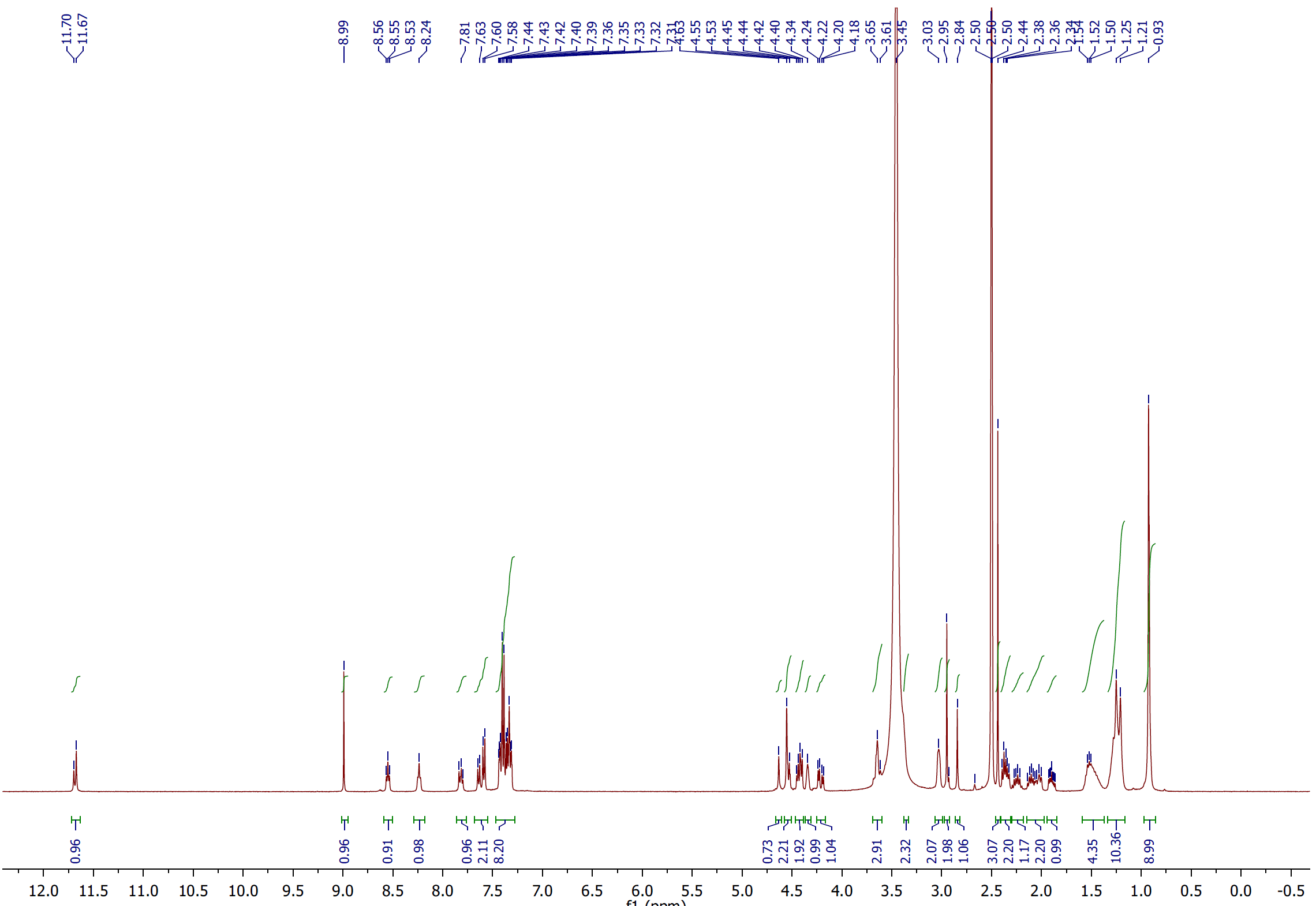


**852181**


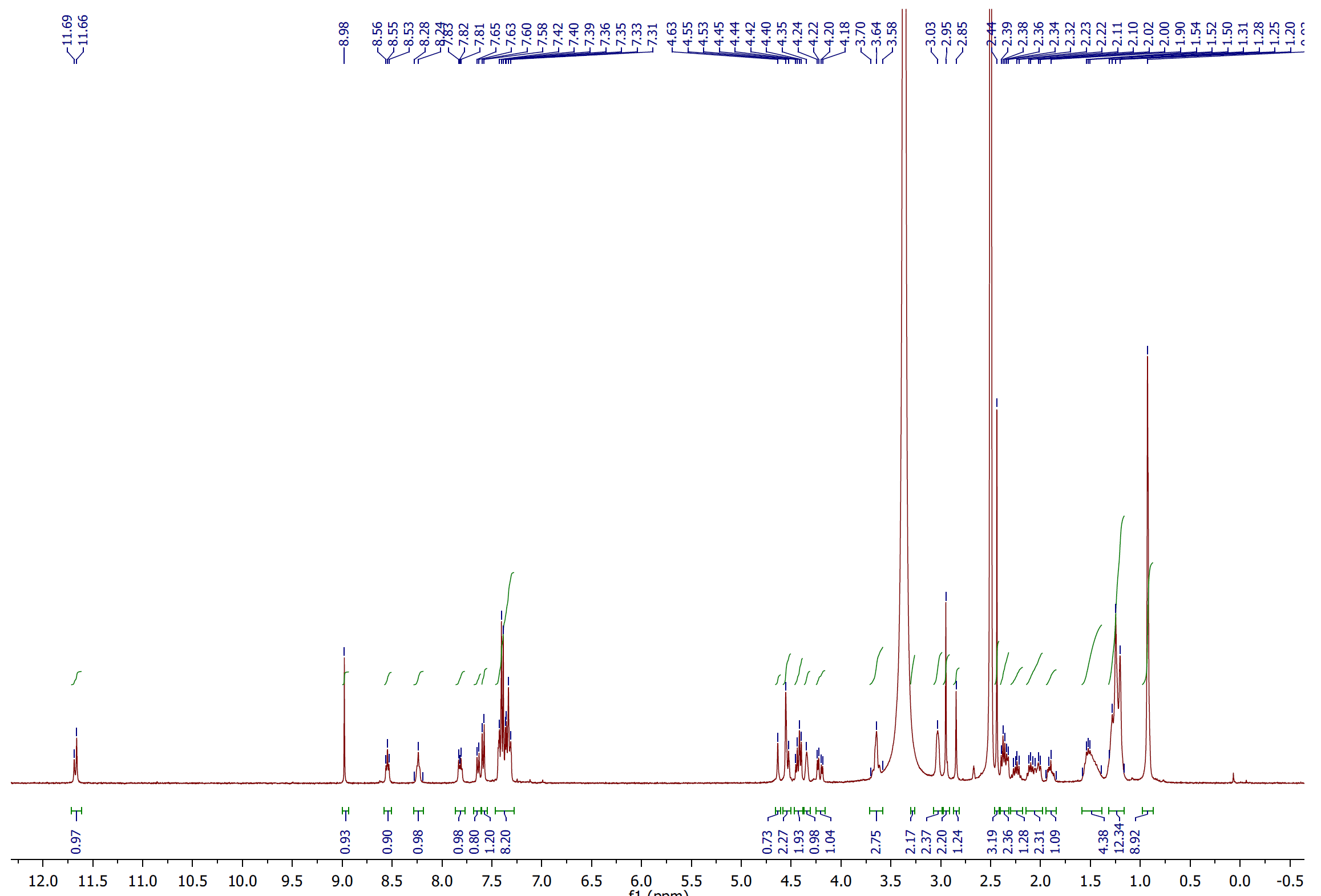


**852195**


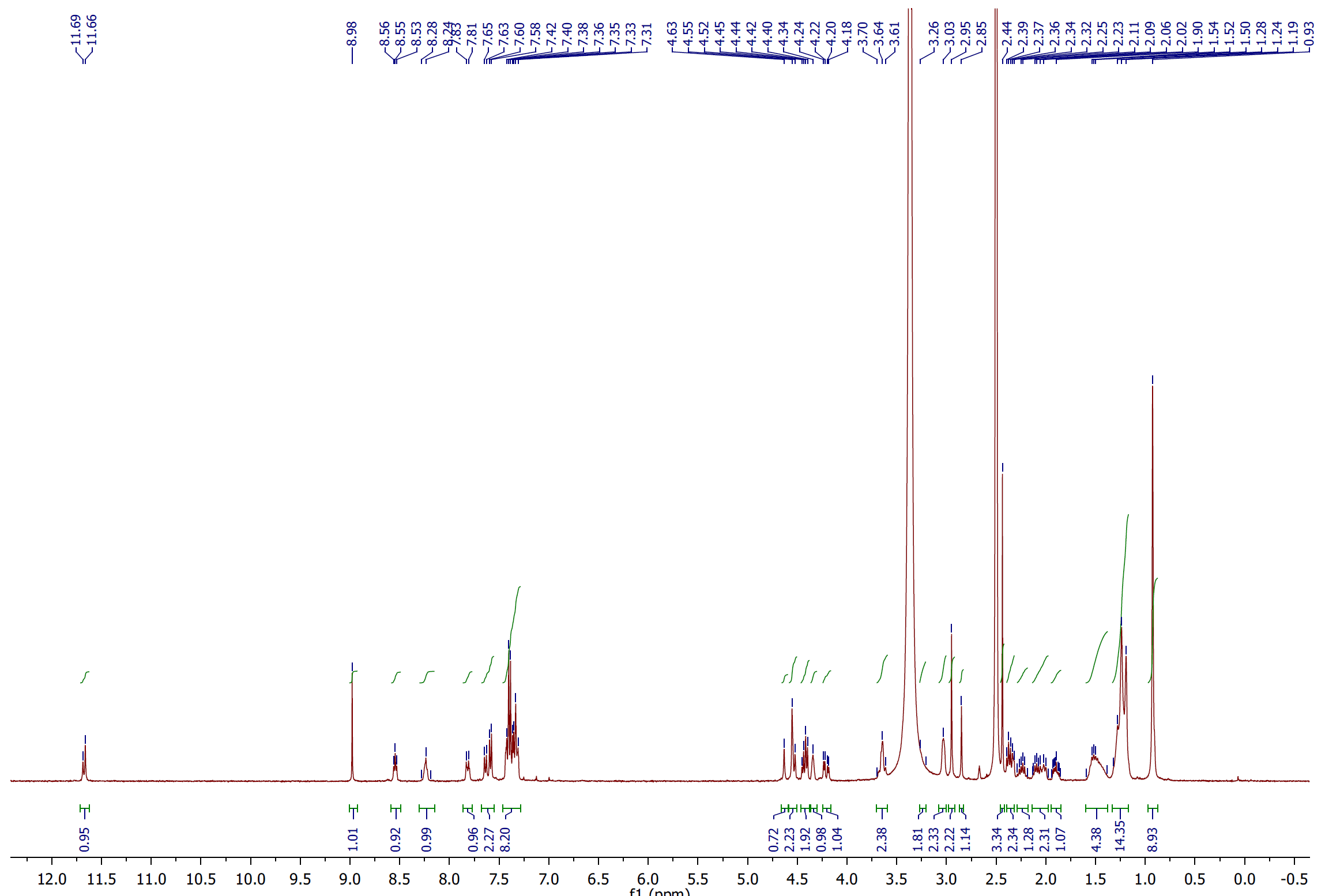


**852203**

**Data S2: Mass spectra related to the chemical synthesis and characterization**


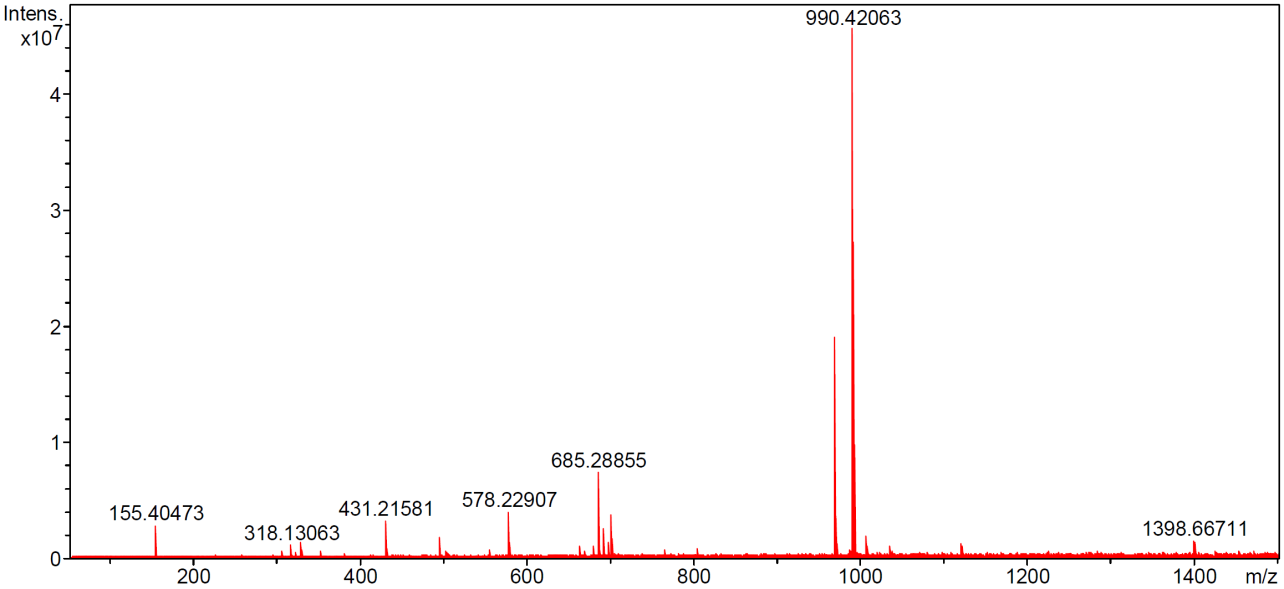


**151098**


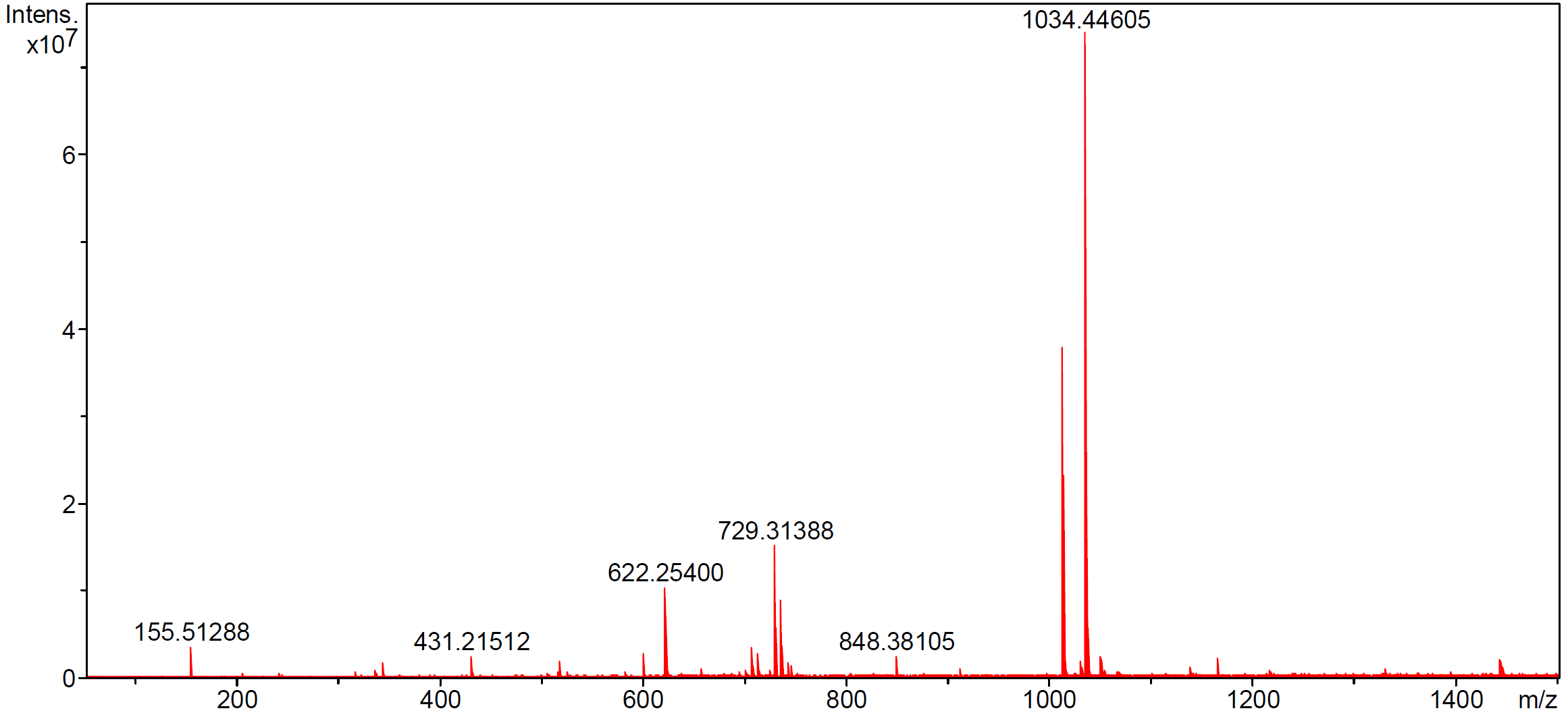


**151099**


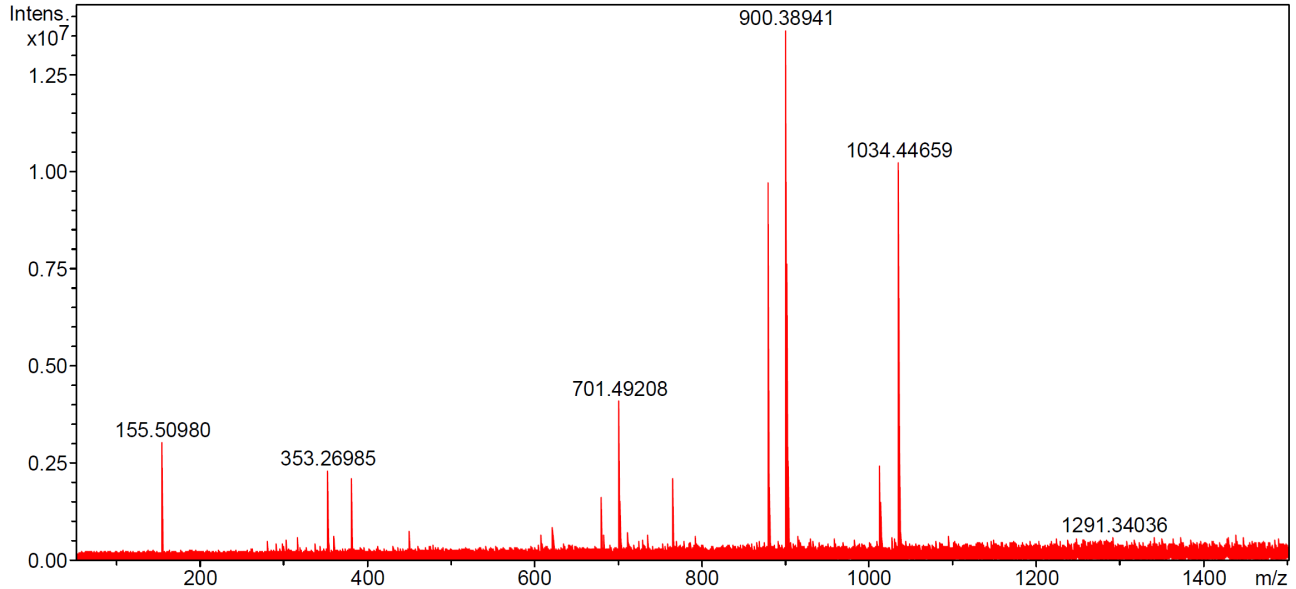


**180052**


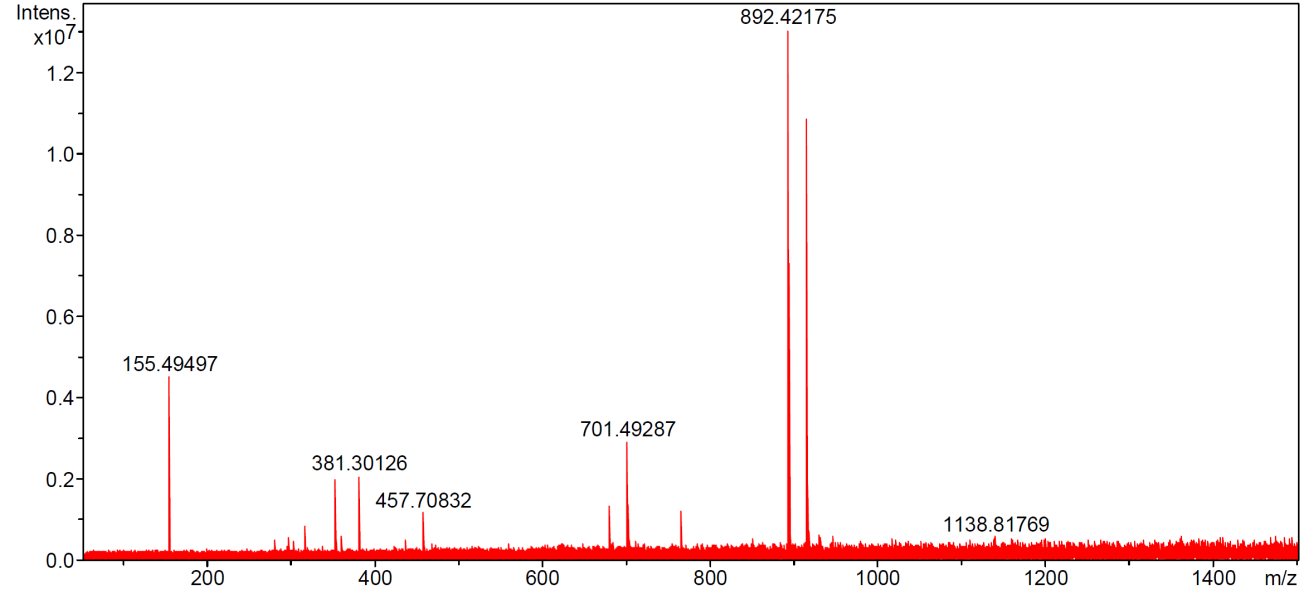


**180053**


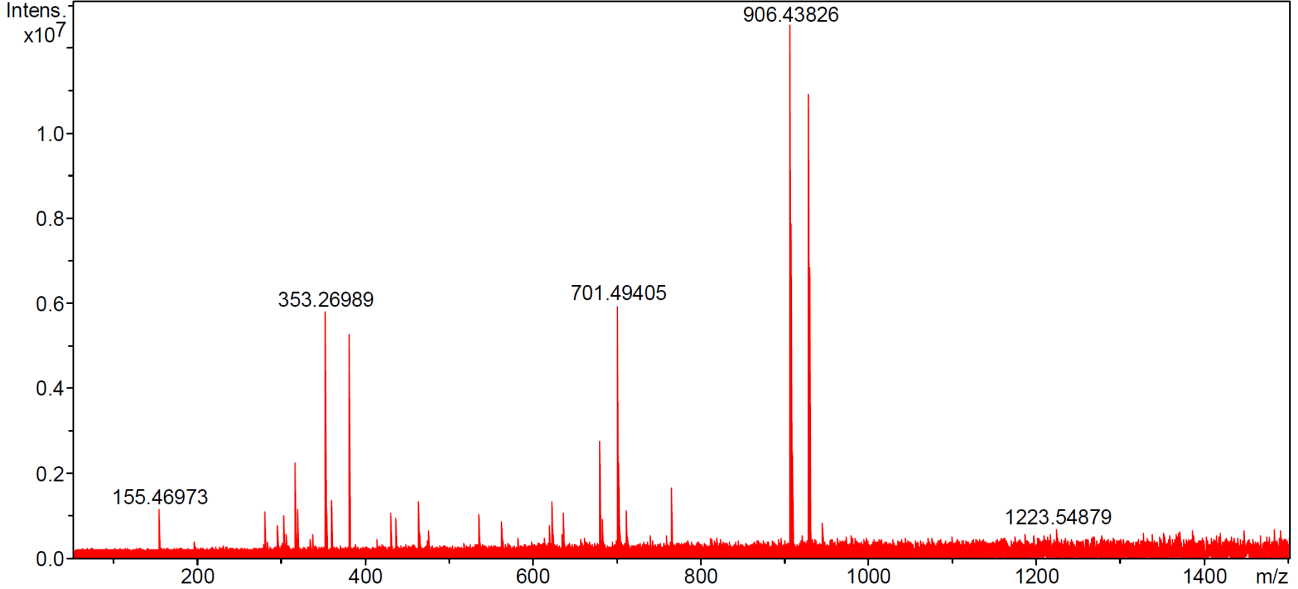


**180054**


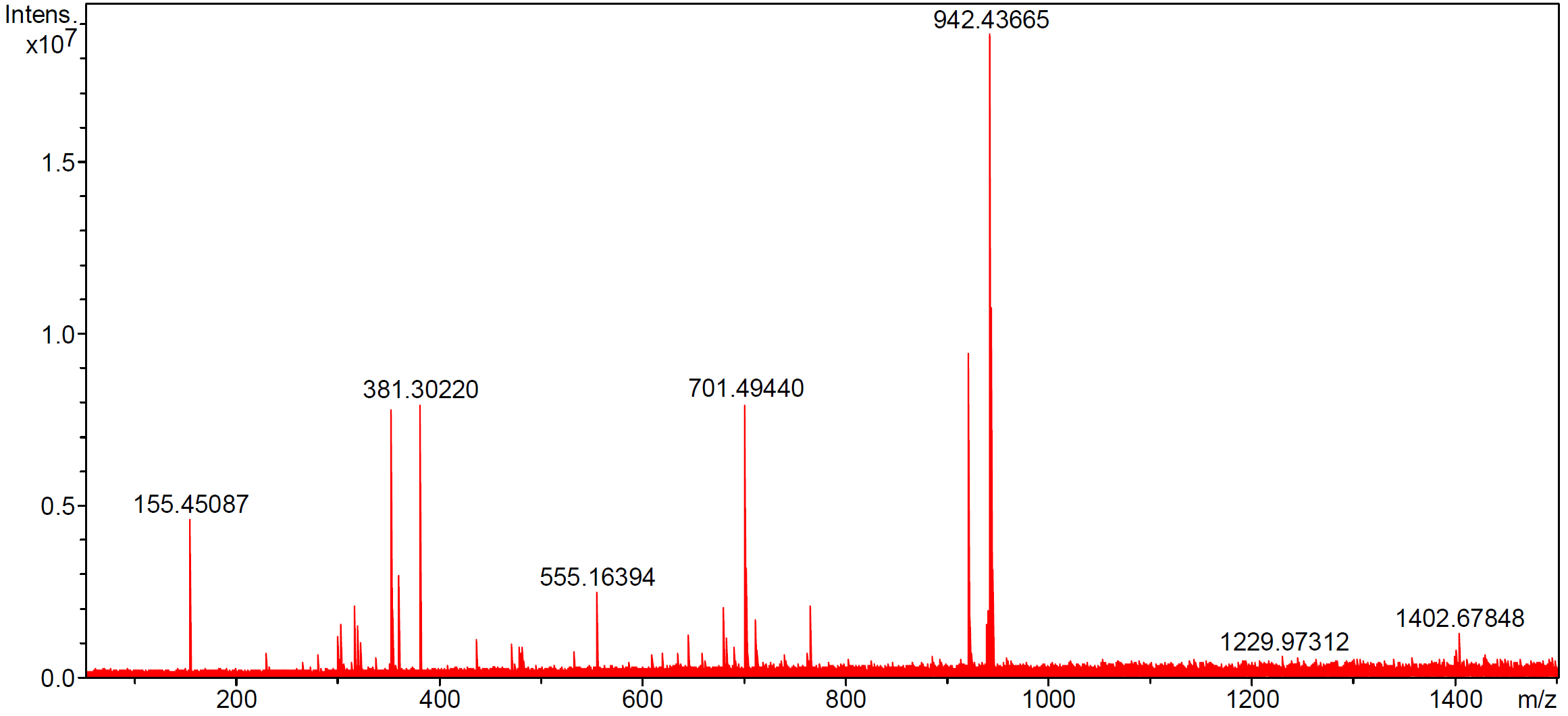


**180055**


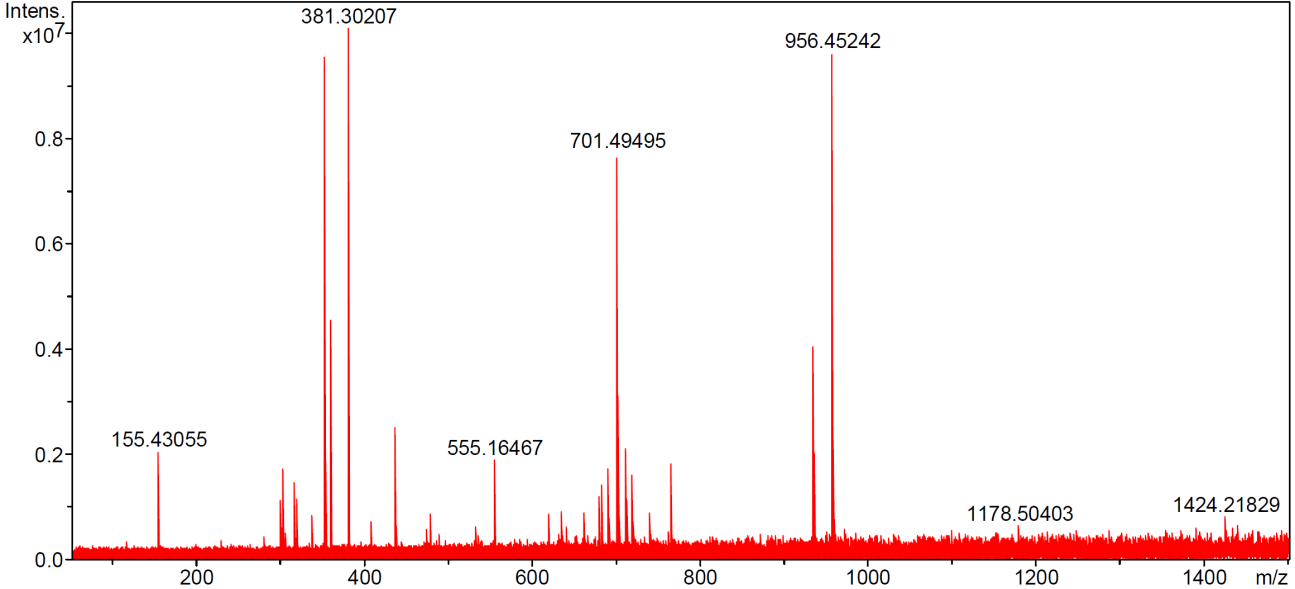


**852181**


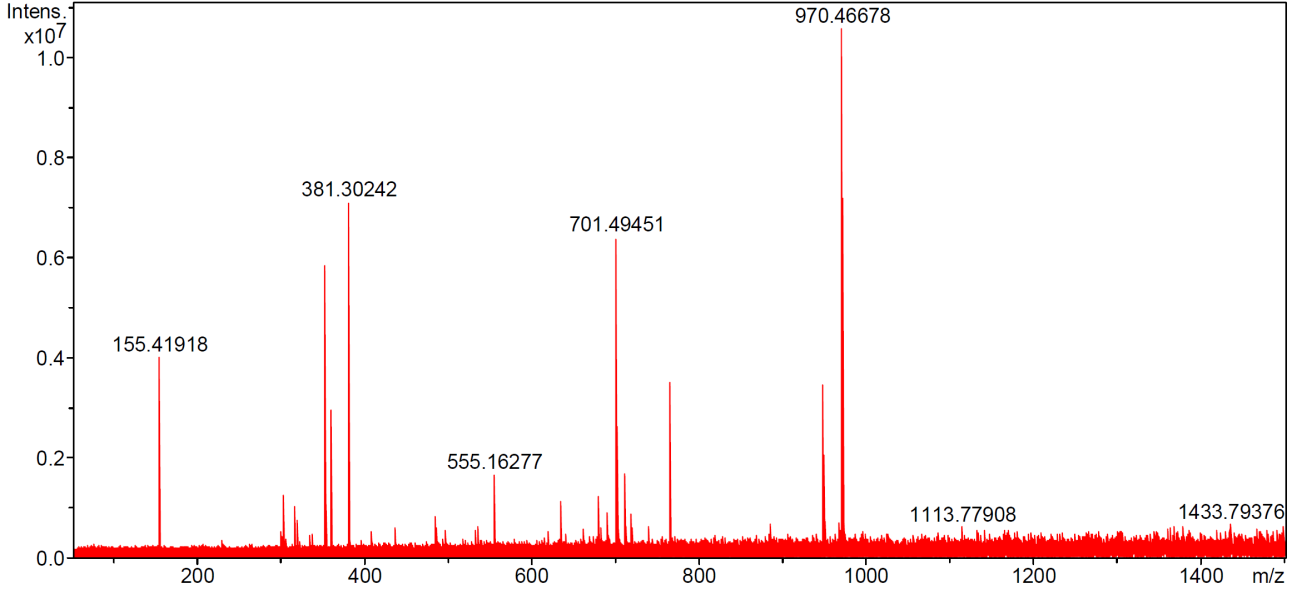


**852195**


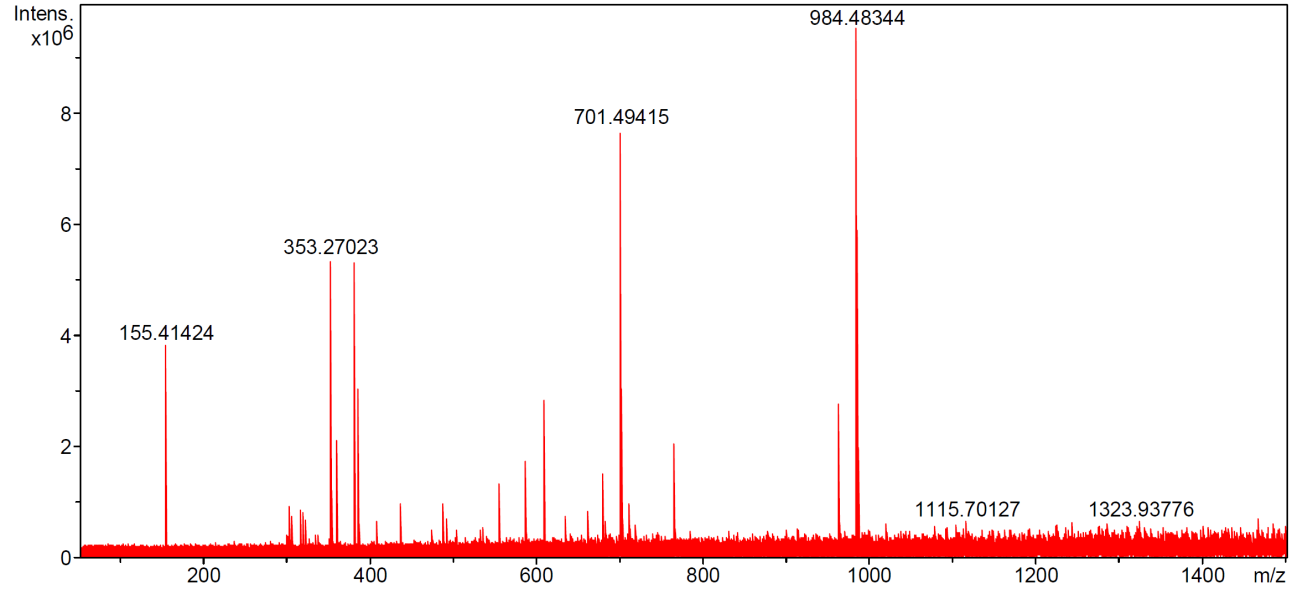


**852203**

**Table S1. Oligonucleotides used in this paper**

|  | **sequence** | **Description** |
| --- | --- | --- |
| VHL-sh1-F | ccggtatcacactgccagtgtatacctcgaggtatacactggcagtgtgatatttttg | For VHL gene knockdown |
| VHL-sh1-R | aattcaaaaatatcacactgccagtgtatacctcgaggtatacactggcagtgtgata | For VHL gene knockdown |
| VHL-sh2-F | ccggtaggattgacattctacagttctcgagaactgtagaatgtcaatcctatttttg | For VHL gene knockdown |
| VHL-sh2-R | aattcaaaaataggattgacattctacagttctcgagaactgtagaatgtcaatccta | For VHL gene knockdown |
| VHL-sh3-F | ccggccatctctcaatgttgacggactcgagtccgtcaacattgagagatggtttttg | For VHL gene knockdown |
| VHL-sh3-R | aattcaaaaaccatctctcaatgttgacggactcgagtccgtcaacattgagagatgg | For VHL gene knockdown |
