# Supplementary material for: Minimizing DNA trapping while maintaining activity inhibition via selective PARP1 degrader
Source: Cell Death Dis. 2024 Dec 18;15(12):898. doi: 10.1038/s41419-024-07277-2 (PMC11655542; doi:10.1038/s41419-024-07277-2)
Supplement: Supplementary file 2 — Western blots [file 41419_2024_7277_MOESM2_ESM.pptx]

## Slide 1
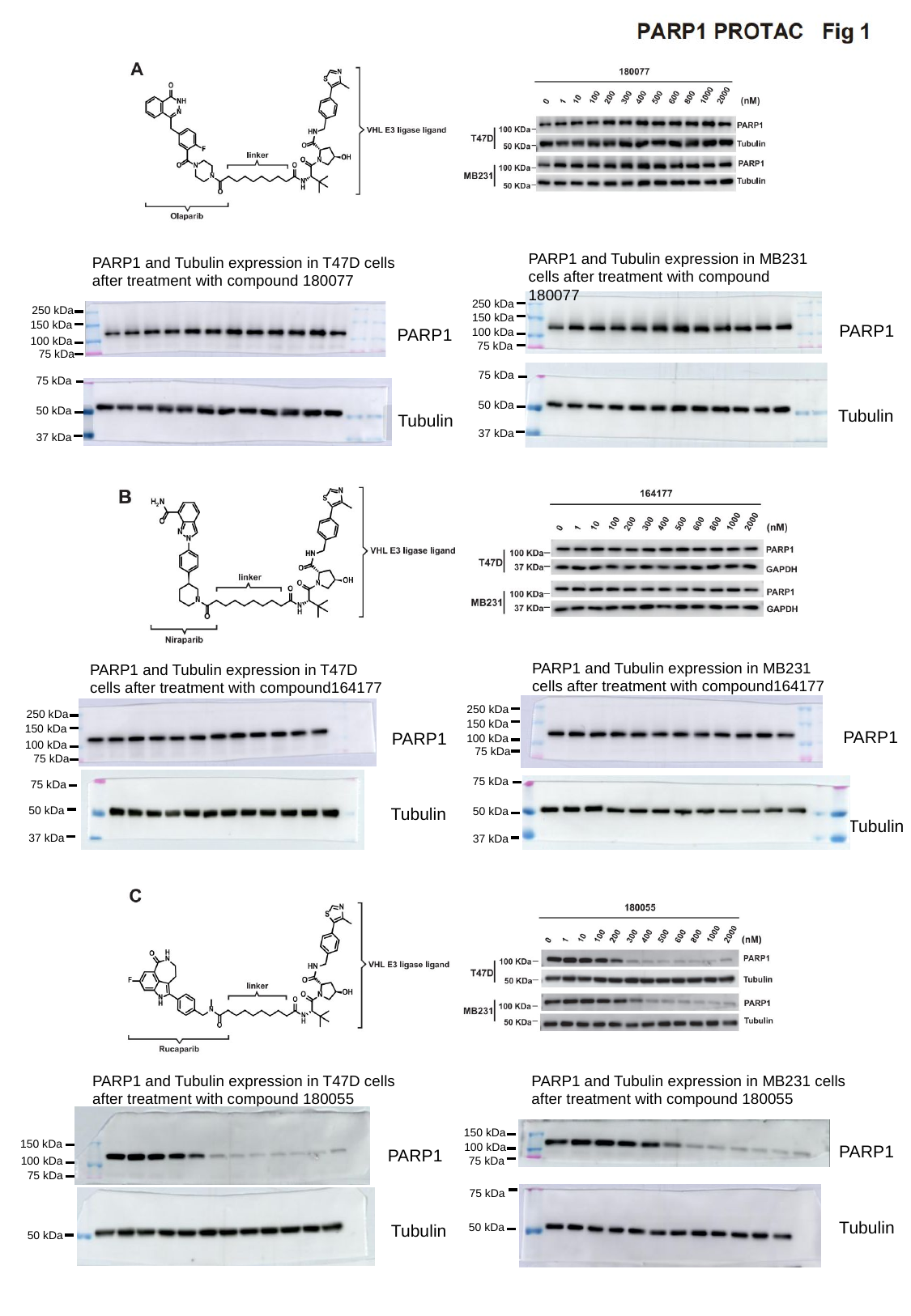

PARP1 and Tubulin expression in MB231 cells after treatment with compound 180077
PARP1 and Tubulin expression in T47D cells after treatment with compound 180077
250 kDa
250 kDa
150 kDa
150 kDa
PARP1
PARP1
100 kDa
100 kDa
75 kDa
75 kDa
75 kDa
75 kDa
50 kDa
50 kDa
Tubulin
Tubulin
37 kDa
37 kDa
PARP1 and Tubulin expression in MB231 cells after treatment with compound164177 164177
PARP1 and Tubulin expression in T47D cells after treatment with compound164177 164177
250 kDa
250 kDa
150 kDa
150 kDa
PARP1
PARP1
100 kDa
100 kDa
75 kDa
75 kDa
75 kDa
75 kDa
50 kDa
Tubulin
50 kDa
Tubulin
37 kDa
37 kDa
PARP1 and Tubulin expression in MB231 cells after treatment with compound 180055
PARP1 and Tubulin expression in T47D cells after treatment with compound 180055
150 kDa
150 kDa
100 kDa
PARP1
PARP1
75 kDa
100 kDa
75 kDa
75 kDa
Tubulin
Tubulin
50 kDa
50 kDa

## Slide 2
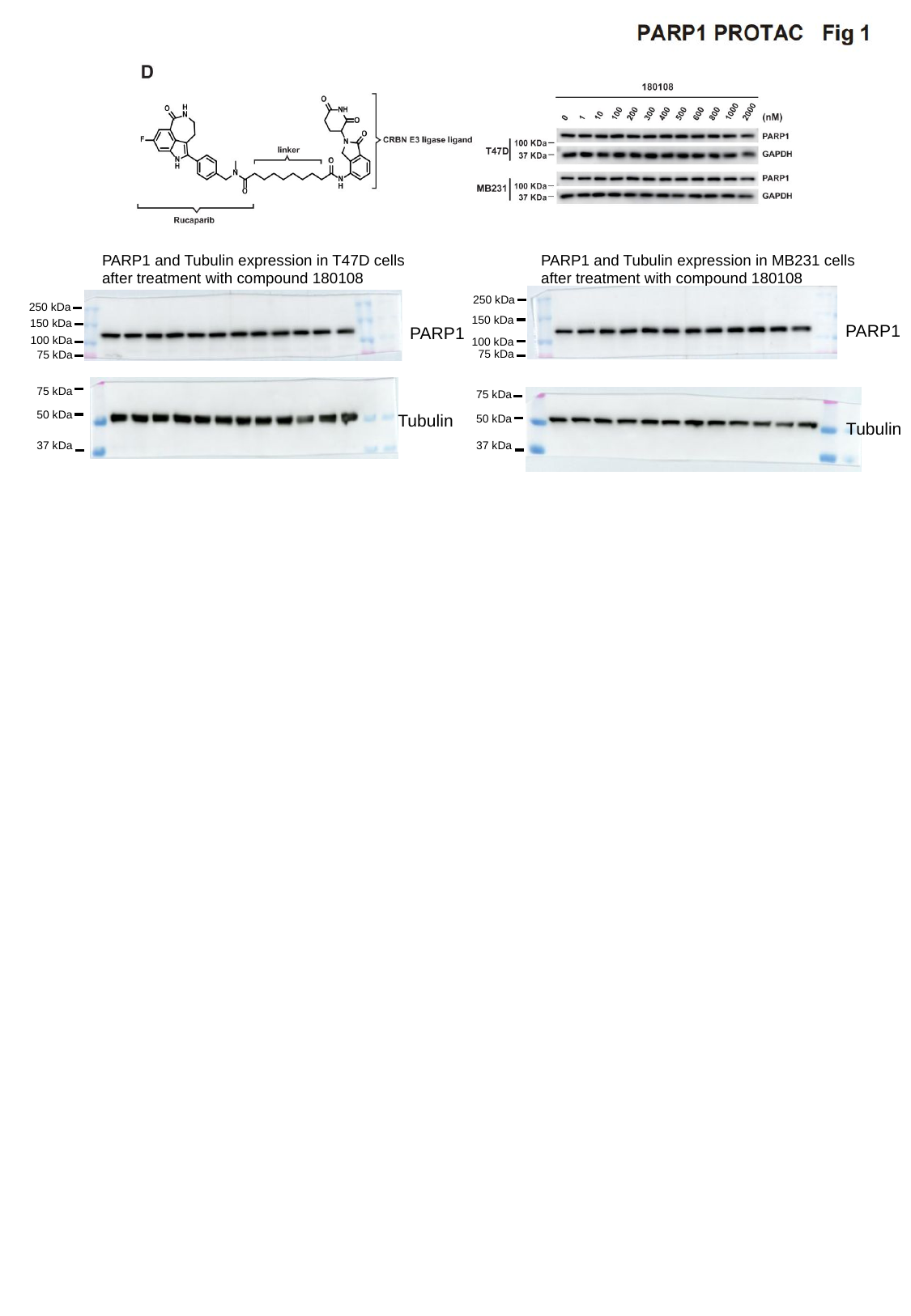

PARP1 and Tubulin expression in MB231 cells after treatment with compound 180108
PARP1 and Tubulin expression in T47D cells after treatment with compound 180108
250 kDa
250 kDa
150 kDa
150 kDa
PARP1
PARP1
100 kDa
100 kDa
75 kDa
75 kDa
75 kDa
75 kDa
50 kDa
Tubulin
50 kDa
Tubulin
37 kDa
37 kDa

## Slide 3
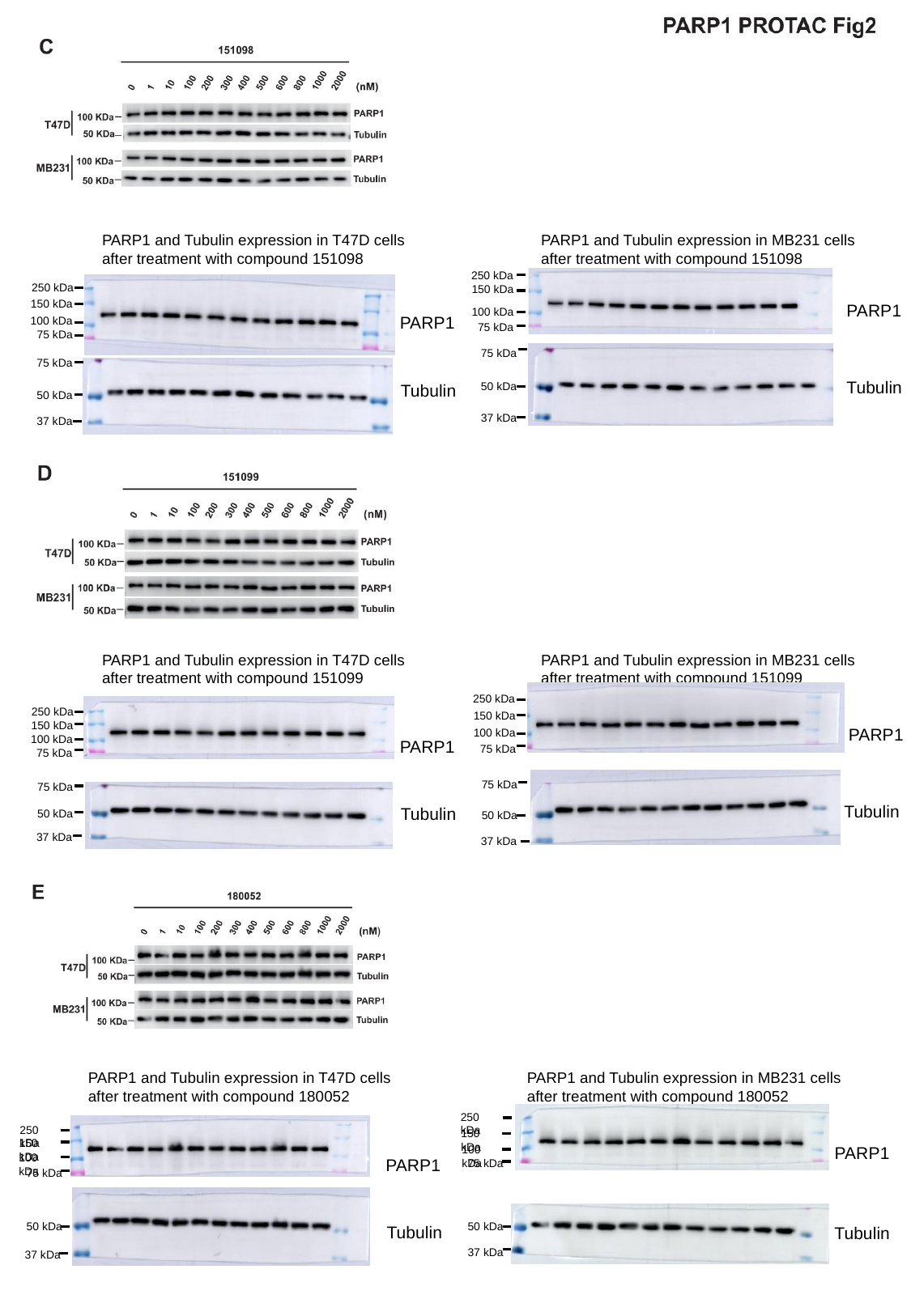

PARP1 and Tubulin expression in MB231 cells after treatment with compound 151098
PARP1 and Tubulin expression in T47D cells after treatment with compound 151098
250 kDa
250 kDa
150 kDa
150 kDa
PARP1
100 kDa
PARP1
100 kDa
75 kDa
75 kDa
75 kDa
75 kDa
Tubulin
50 kDa
Tubulin
50 kDa
37 kDa
37 kDa
PARP1 and Tubulin expression in MB231 cells after treatment with compound 151099
PARP1 and Tubulin expression in T47D cells after treatment with compound 151099
250 kDa
250 kDa
150 kDa
150 kDa
PARP1
100 kDa
100 kDa
PARP1
75 kDa
75 kDa
75 kDa
75 kDa
Tubulin
Tubulin
50 kDa
50 kDa
37 kDa
37 kDa
PARP1 and Tubulin expression in MB231 cells after treatment with compound 180052
PARP1 and Tubulin expression in T47D cells after treatment with compound 180052
250 kDa
250 kDa
150 kDa
150 kDa
PARP1
100 kDa
100 kDa
PARP1
75 kDa
75 kDa
50 kDa
50 kDa
Tubulin
Tubulin
37 kDa
37 kDa

## Slide 4
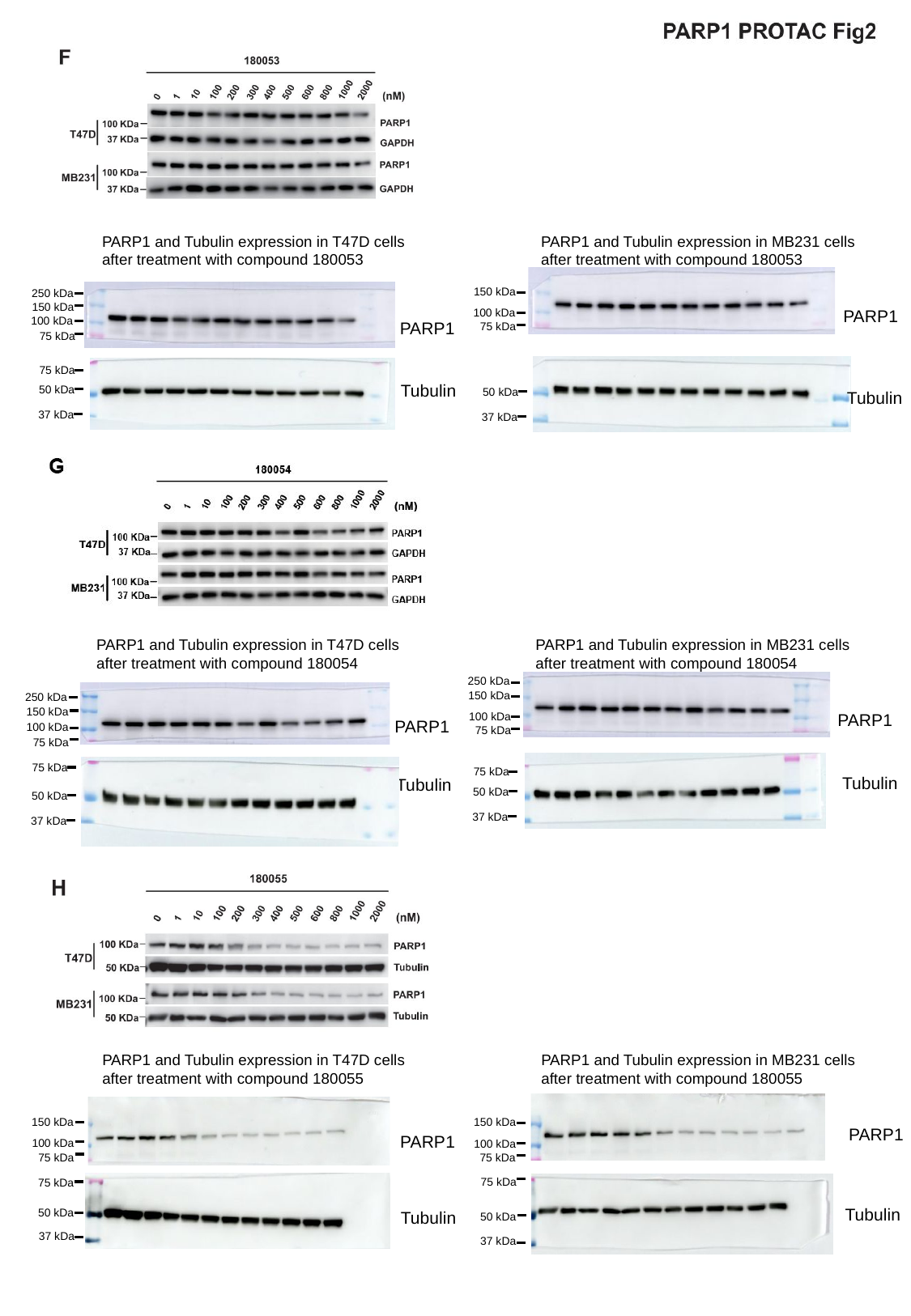

PARP1 and Tubulin expression in MB231 cells after treatment with compound 180053
PARP1 and Tubulin expression in T47D cells after treatment with compound 180053
150 kDa
250 kDa
150 kDa
PARP1
100 kDa
100 kDa
PARP1
75 kDa
75 kDa
75 kDa
Tubulin
50 kDa
50 kDa
Tubulin
37 kDa
37 kDa
PARP1 and Tubulin expression in MB231 cells after treatment with compound 180054
PARP1 and Tubulin expression in T47D cells after treatment with compound 180054
250 kDa
150 kDa
250 kDa
150 kDa
PARP1
100 kDa
PARP1
100 kDa
75 kDa
75 kDa
75 kDa
75 kDa
Tubulin
Tubulin
50 kDa
50 kDa
37 kDa
37 kDa
PARP1 and Tubulin expression in MB231 cells after treatment with compound 180055
PARP1 and Tubulin expression in T47D cells after treatment with compound 180055
150 kDa
150 kDa
PARP1
PARP1
100 kDa
100 kDa
75 kDa
75 kDa
75 kDa
75 kDa
Tubulin
50 kDa
Tubulin
50 kDa
37 kDa
37 kDa

## Slide 5
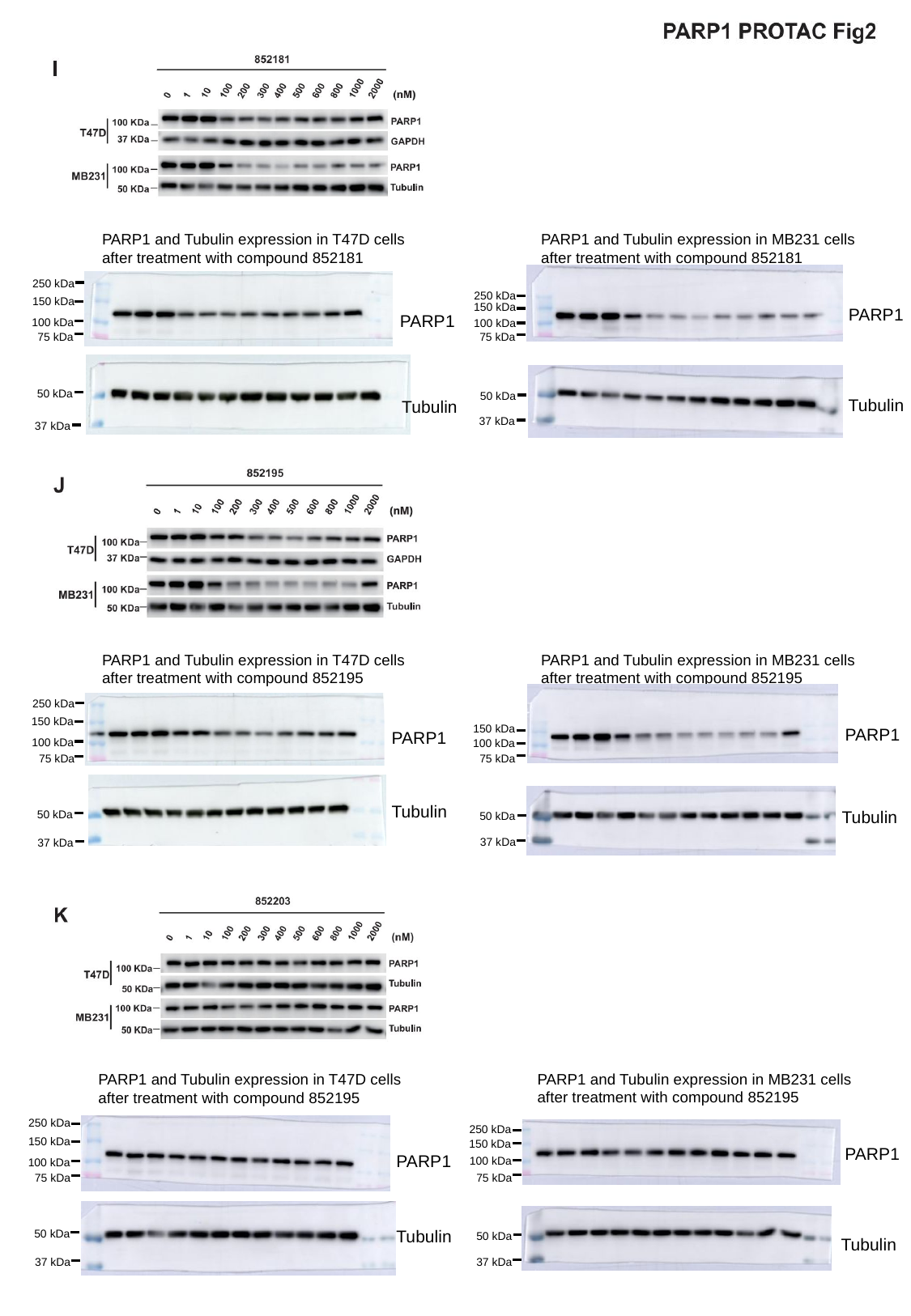

PARP1 and Tubulin expression in MB231 cells after treatment with compound 852181
PARP1 and Tubulin expression in T47D cells after treatment with compound 852181
250 kDa
250 kDa
150 kDa
150 kDa
PARP1
PARP1
100 kDa
100 kDa
75 kDa
75 kDa
50 kDa
50 kDa
Tubulin
Tubulin
37 kDa
37 kDa
PARP1 and Tubulin expression in MB231 cells after treatment with compound 852195
PARP1 and Tubulin expression in T47D cells after treatment with compound 852195
250 kDa
150 kDa
150 kDa
PARP1
PARP1
100 kDa
100 kDa
75 kDa
75 kDa
Tubulin
Tubulin
50 kDa
50 kDa
37 kDa
37 kDa
PARP1 and Tubulin expression in MB231 cells after treatment with compound 852195
PARP1 and Tubulin expression in T47D cells after treatment with compound 852195
250 kDa
250 kDa
150 kDa
150 kDa
PARP1
PARP1
100 kDa
100 kDa
75 kDa
75 kDa
Tubulin
50 kDa
50 kDa
Tubulin
37 kDa
37 kDa

## Slide 6
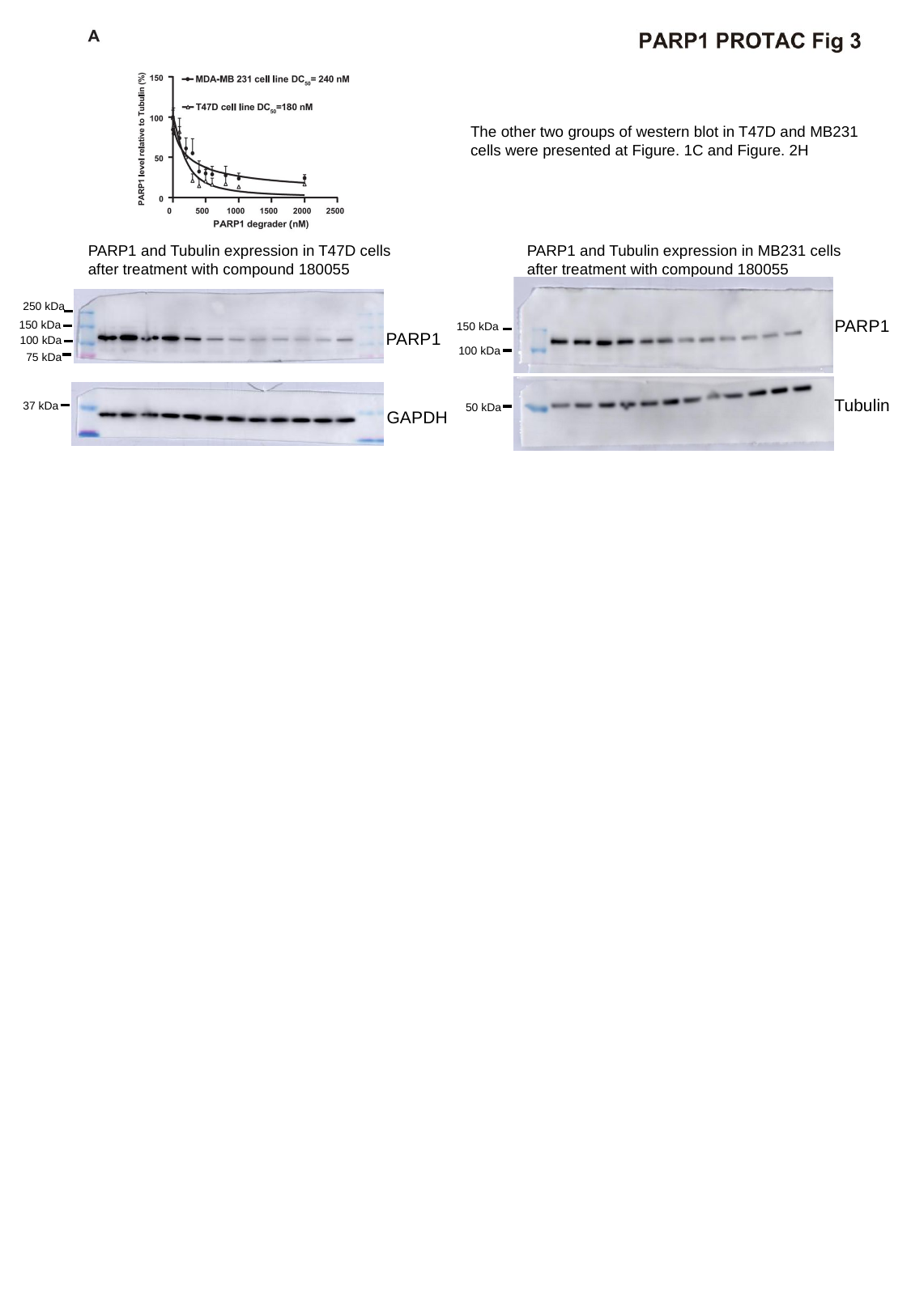

The other two groups of western blot in T47D and MB231 cells were presented at Figure. 1C and Figure. 2H
PARP1 and Tubulin expression in MB231 cells after treatment with compound 180055
PARP1 and Tubulin expression in T47D cells after treatment with compound 180055
250 kDa
PARP1
150 kDa
150 kDa
PARP1
100 kDa
100 kDa
75 kDa
Tubulin
37 kDa
50 kDa
GAPDH

## Slide 7
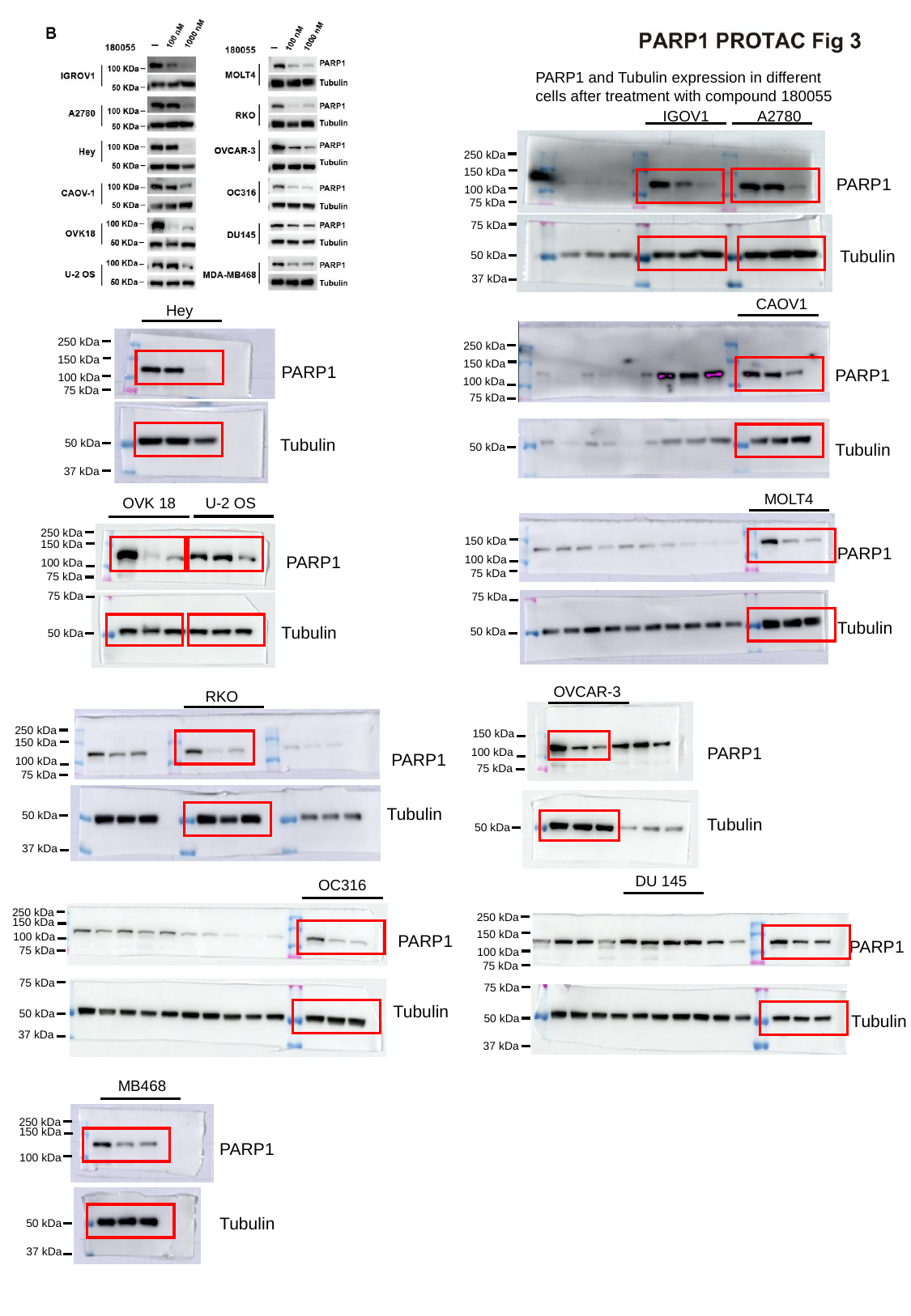

PARP1 and Tubulin expression in different cells after treatment with compound 180055
IGOV1
A2780
250 kDa
150 kDa
PARP1
100 kDa
75 kDa
75 kDa
Tubulin
50 kDa
37 kDa
CAOV1
Hey
250 kDa
250 kDa
150 kDa
150 kDa
PARP1
PARP1
100 kDa
100 kDa
75 kDa
75 kDa
Tubulin
50 kDa
Tubulin
50 kDa
37 kDa
MOLT4
OVK 18
U-2 OS
250 kDa
150 kDa
150 kDa
PARP1
PARP1
100 kDa
100 kDa
75 kDa
75 kDa
75 kDa
75 kDa
Tubulin
Tubulin
50 kDa
50 kDa
OVCAR-3
RKO
250 kDa
150 kDa
150 kDa
PARP1
100 kDa
PARP1
100 kDa
75 kDa
75 kDa
Tubulin
50 kDa
Tubulin
50 kDa
37 kDa
DU 145
OC316
250 kDa
250 kDa
150 kDa
150 kDa
100 kDa
PARP1
PARP1
75 kDa
100 kDa
75 kDa
75 kDa
75 kDa
Tubulin
50 kDa
Tubulin
50 kDa
37 kDa
37 kDa
MB468
250 kDa
150 kDa
PARP1
100 kDa
Tubulin
50 kDa
37 kDa

## Slide 8
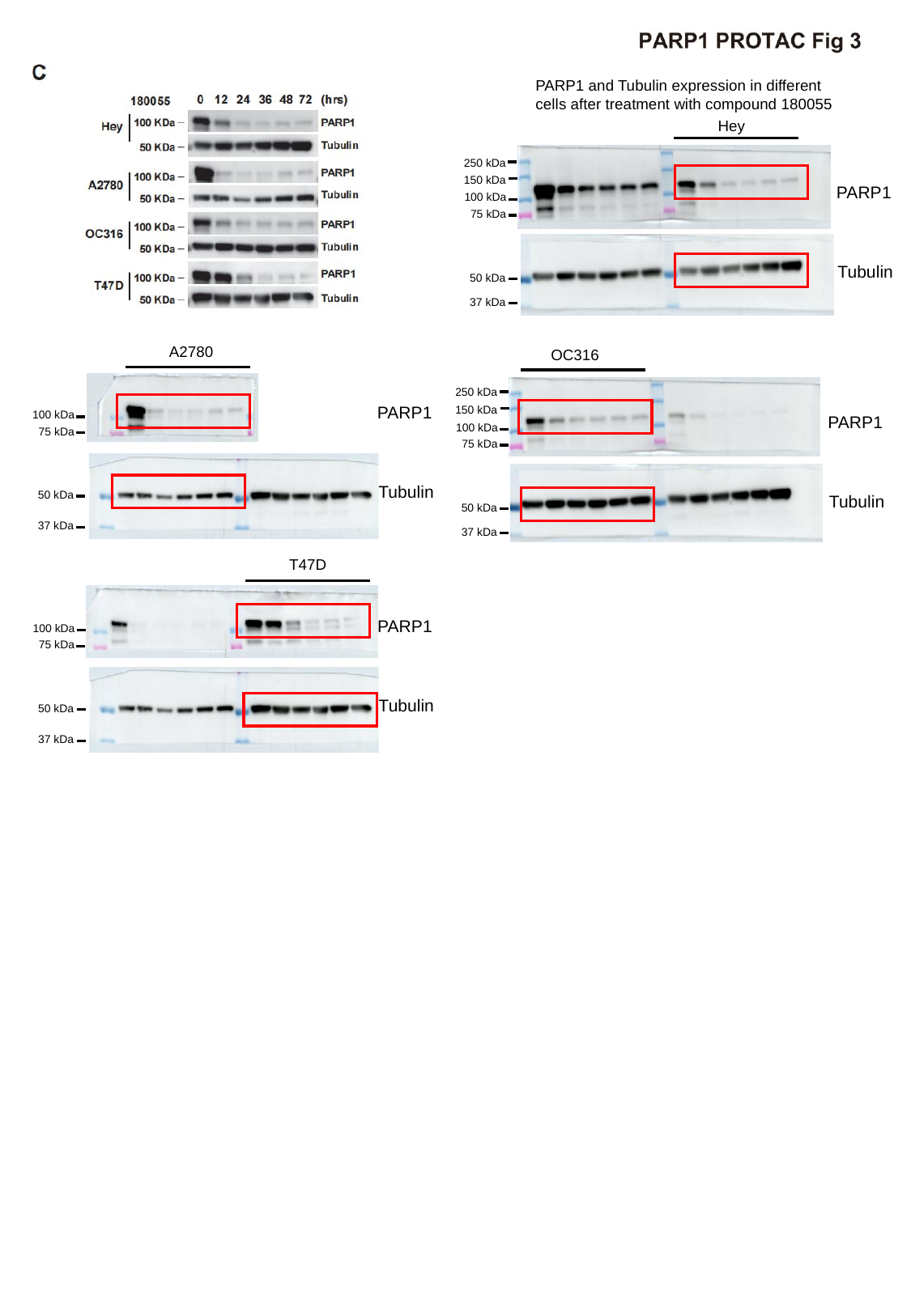

PARP1 and Tubulin expression in different cells after treatment with compound 180055
Hey
250 kDa
150 kDa
PARP1
100 kDa
75 kDa
Tubulin
50 kDa
37 kDa
A2780
OC316
250 kDa
PARP1
150 kDa
100 kDa
PARP1
100 kDa
75 kDa
75 kDa
Tubulin
50 kDa
Tubulin
50 kDa
37 kDa
37 kDa
T47D
PARP1
100 kDa
75 kDa
Tubulin
50 kDa
37 kDa

## Slide 9
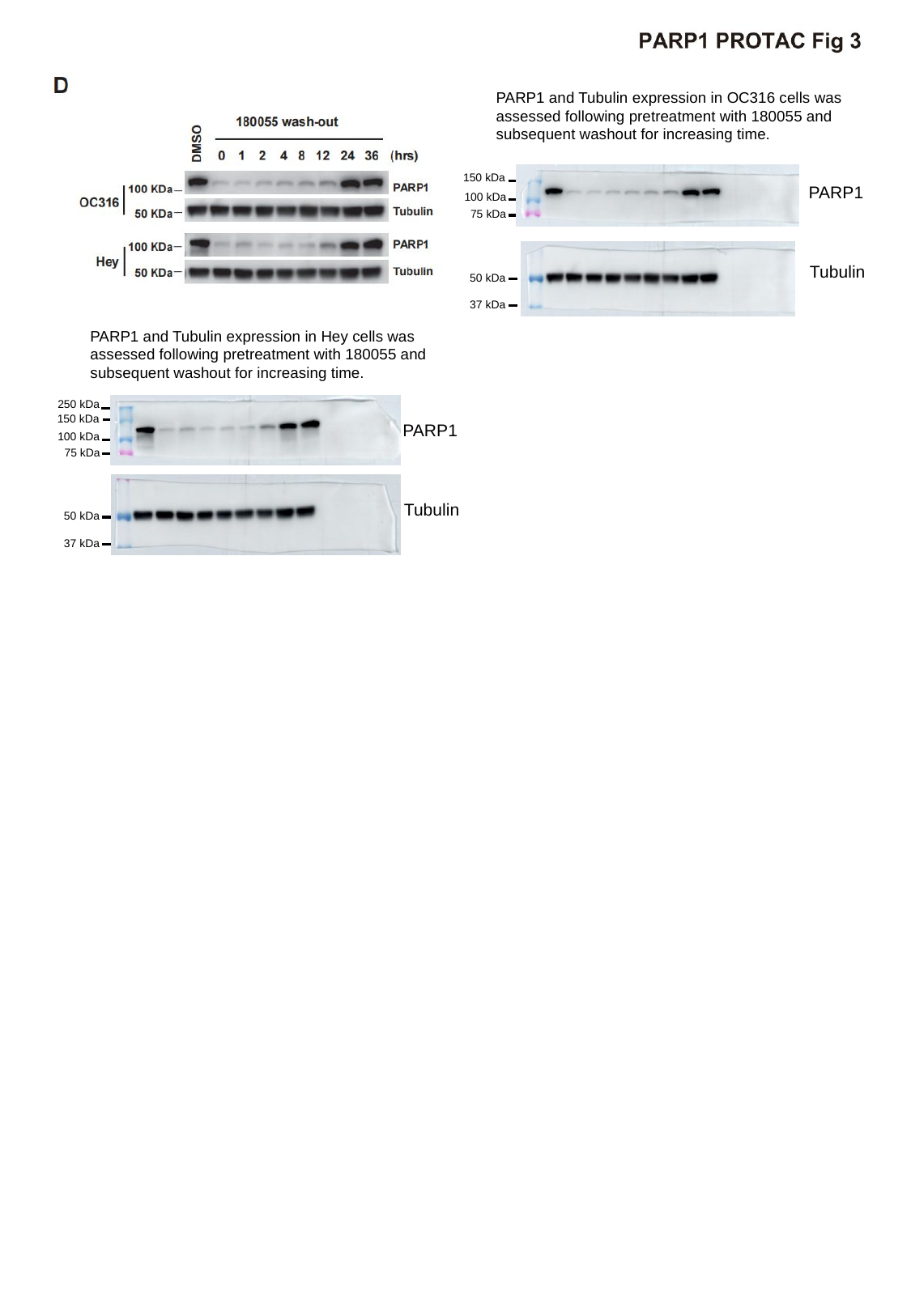

PARP1 and Tubulin expression in OC316 cells was assessed following pretreatment with 180055 and subsequent washout for increasing time.
150 kDa
PARP1
100 kDa
75 kDa
Tubulin
50 kDa
37 kDa
PARP1 and Tubulin expression in Hey cells was assessed following pretreatment with 180055 and subsequent washout for increasing time.
250 kDa
150 kDa
PARP1
100 kDa
75 kDa
Tubulin
50 kDa
37 kDa

## Slide 10
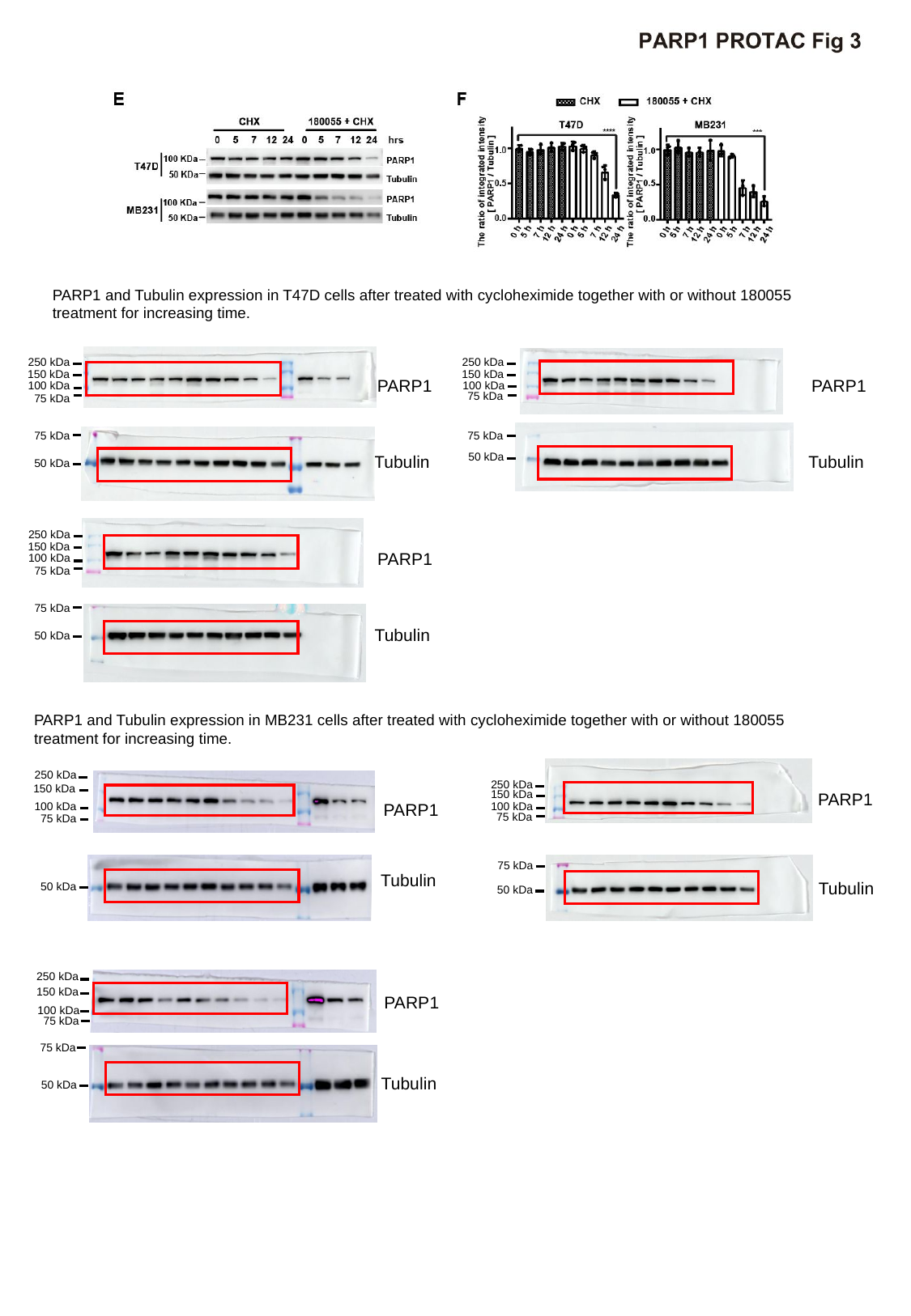

PARP1 and Tubulin expression in T47D cells after treated with cycloheximide together with or without 180055 treatment for increasing time.
250 kDa
250 kDa
150 kDa
150 kDa
PARP1
PARP1
100 kDa
100 kDa
75 kDa
75 kDa
75 kDa
75 kDa
50 kDa
Tubulin
Tubulin
50 kDa
250 kDa
150 kDa
PARP1
100 kDa
75 kDa
75 kDa
Tubulin
50 kDa
PARP1 and Tubulin expression in MB231 cells after treated with cycloheximide together with or without 180055 treatment for increasing time.
250 kDa
250 kDa
150 kDa
150 kDa
PARP1
100 kDa
PARP1
100 kDa
75 kDa
75 kDa
75 kDa
Tubulin
Tubulin
50 kDa
50 kDa
250 kDa
150 kDa
PARP1
100 kDa
75 kDa
75 kDa
Tubulin
50 kDa

## Slide 11
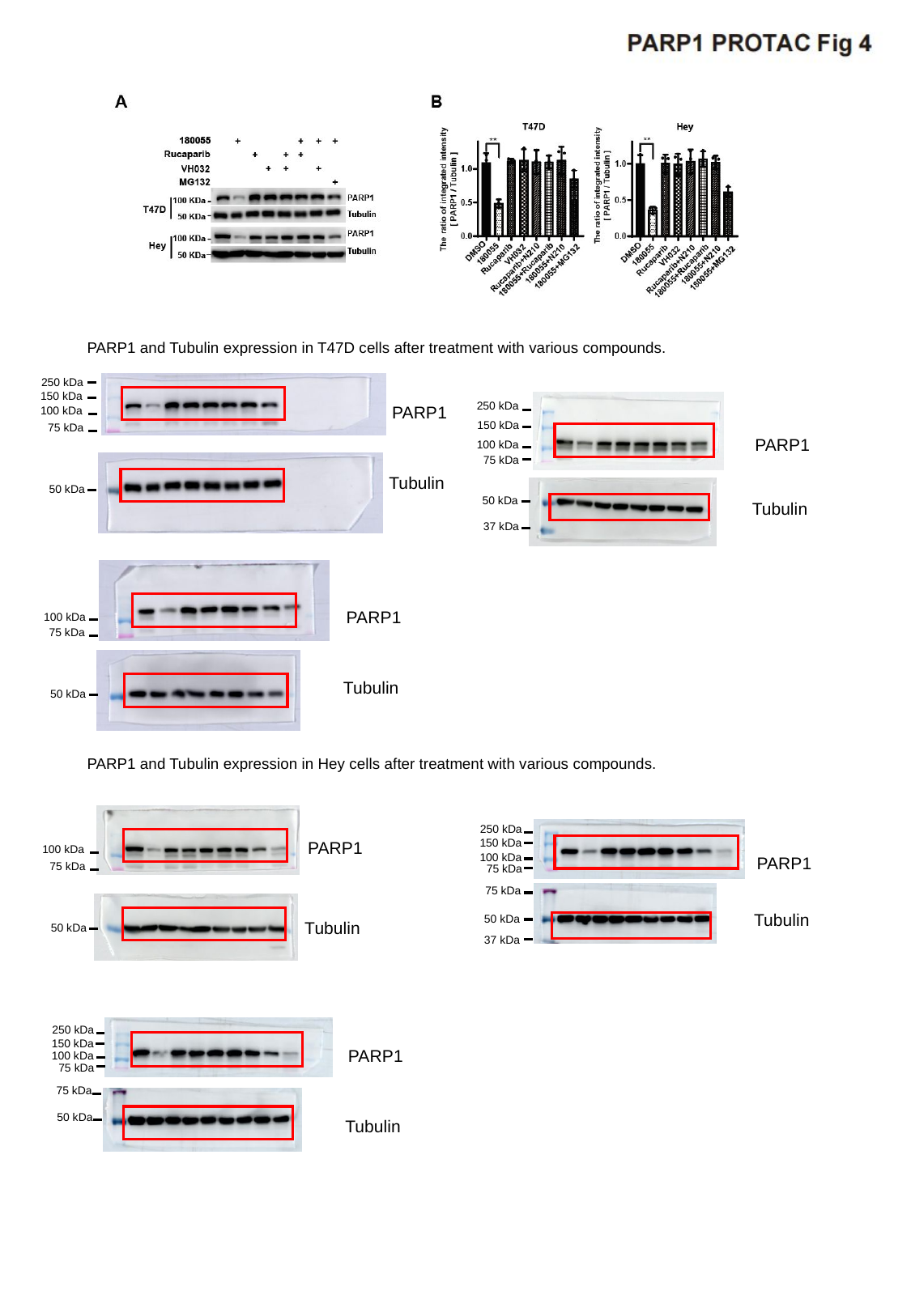

PARP1 and Tubulin expression in T47D cells after treatment with various compounds.
250 kDa
150 kDa
250 kDa
PARP1
100 kDa
150 kDa
75 kDa
PARP1
100 kDa
75 kDa
Tubulin
50 kDa
50 kDa
Tubulin
37 kDa
PARP1
100 kDa
75 kDa
Tubulin
50 kDa
PARP1 and Tubulin expression in Hey cells after treatment with various compounds.
250 kDa
150 kDa
PARP1
100 kDa
100 kDa
PARP1
75 kDa
75 kDa
75 kDa
Tubulin
50 kDa
Tubulin
50 kDa
37 kDa
250 kDa
150 kDa
PARP1
100 kDa
75 kDa
75 kDa
50 kDa
Tubulin

## Slide 12
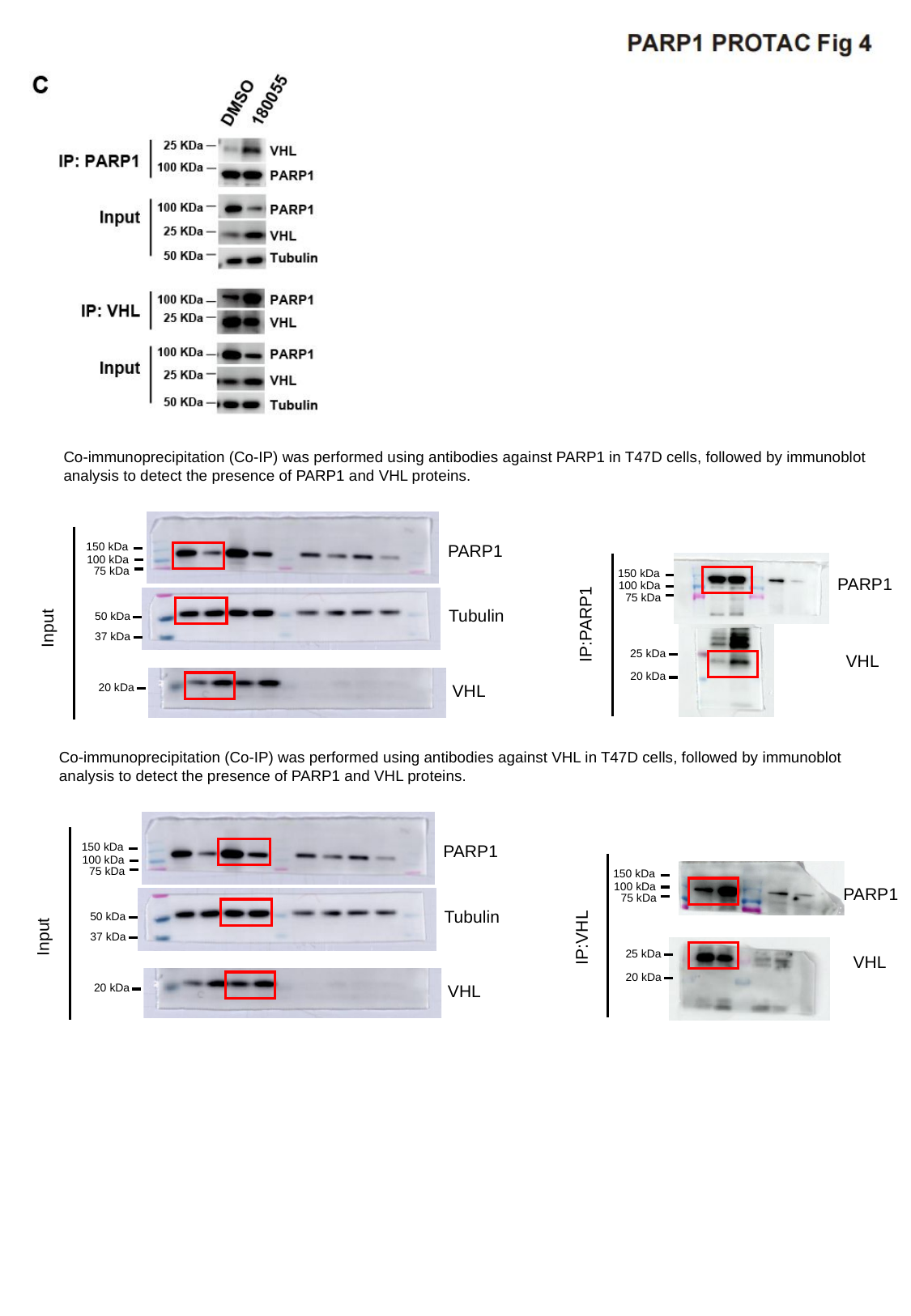

Co-immunoprecipitation (Co-IP) was performed using antibodies against PARP1 in T47D cells, followed by immunoblot analysis to detect the presence of PARP1 and VHL proteins.
150 kDa
PARP1
100 kDa
75 kDa
150 kDa
PARP1
100 kDa
75 kDa
Tubulin
50 kDa
IP:PARP1
Input
37 kDa
25 kDa
VHL
20 kDa
20 kDa
VHL
Co-immunoprecipitation (Co-IP) was performed using antibodies against VHL in T47D cells, followed by immunoblot analysis to detect the presence of PARP1 and VHL proteins.
150 kDa
PARP1
100 kDa
75 kDa
150 kDa
100 kDa
PARP1
75 kDa
Tubulin
50 kDa
IP:VHL
Input
37 kDa
25 kDa
VHL
20 kDa
20 kDa
VHL

## Slide 13
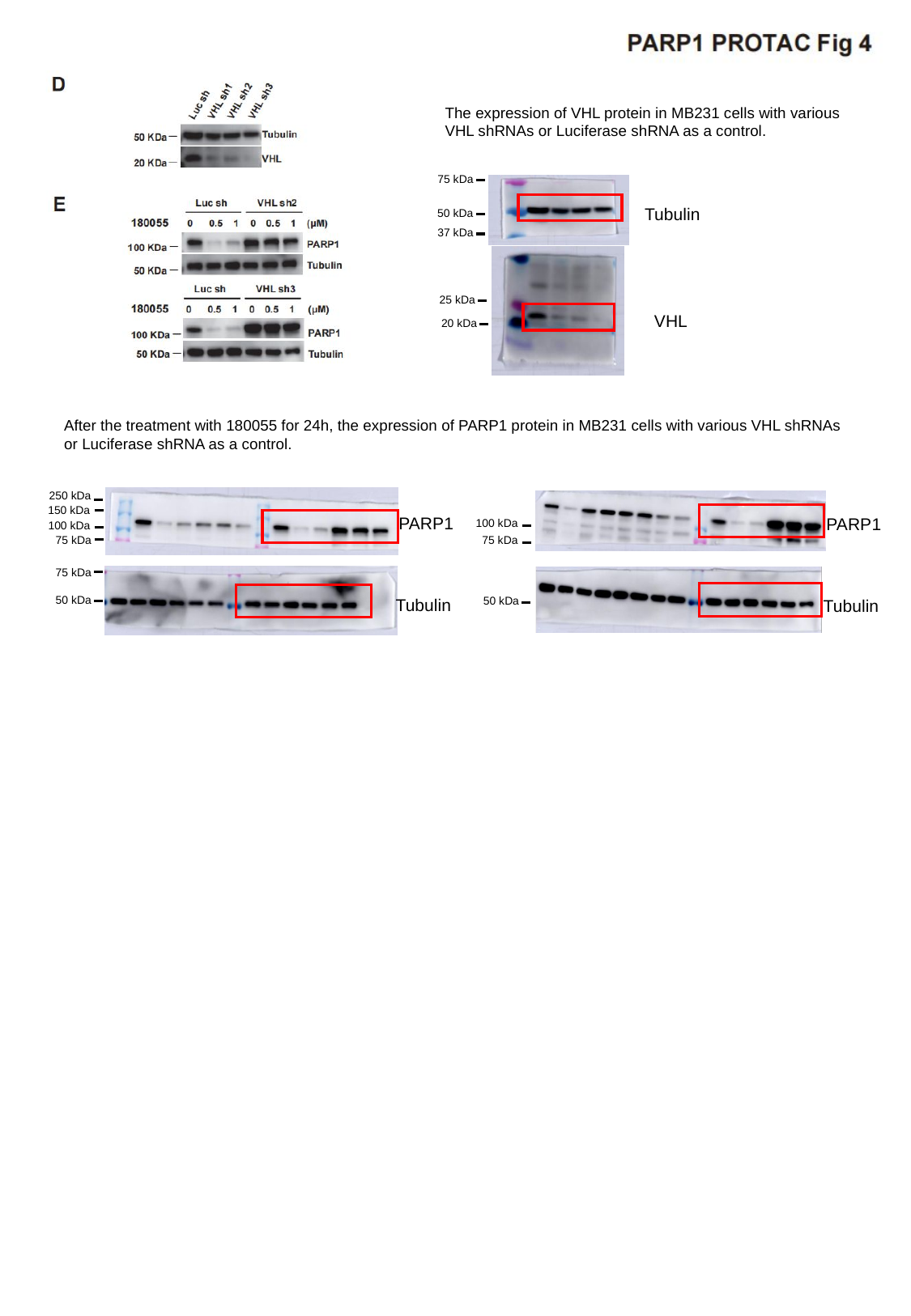

The expression of VHL protein in MB231 cells with various VHL shRNAs or Luciferase shRNA as a control.
75 kDa
Tubulin
50 kDa
37 kDa
25 kDa
VHL
20 kDa
After the treatment with 180055 for 24h, the expression of PARP1 protein in MB231 cells with various VHL shRNAs or Luciferase shRNA as a control.
250 kDa
150 kDa
PARP1
PARP1
100 kDa
100 kDa
75 kDa
75 kDa
75 kDa
50 kDa
Tubulin
50 kDa
Tubulin

## Slide 14
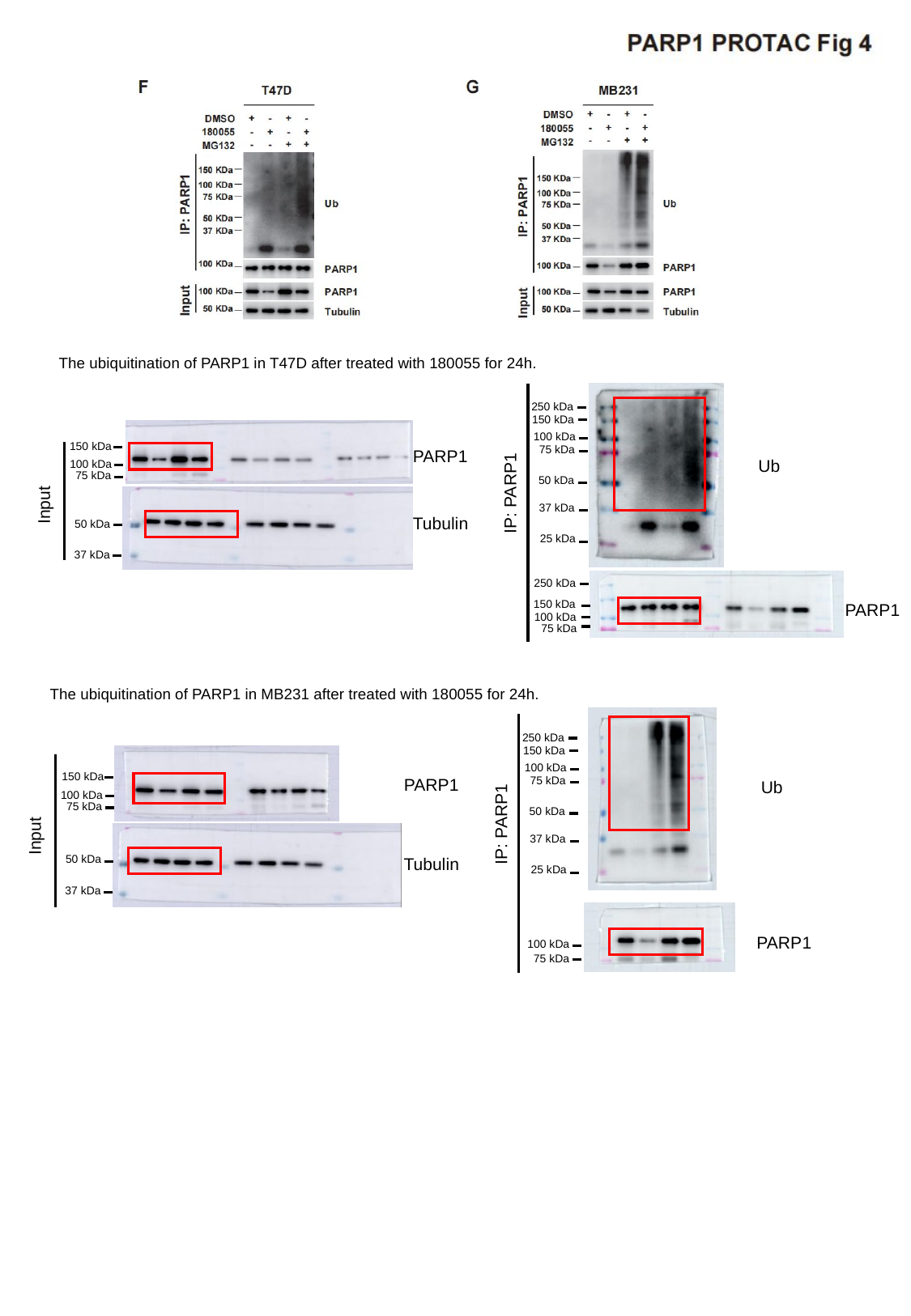

The ubiquitination of PARP1 in T47D after treated with 180055 for 24h.
250 kDa
150 kDa
100 kDa
150 kDa
75 kDa
PARP1
Ub
100 kDa
75 kDa
50 kDa
IP: PARP1
Input
37 kDa
Tubulin
50 kDa
25 kDa
37 kDa
250 kDa
150 kDa
PARP1
100 kDa
75 kDa
The ubiquitination of PARP1 in MB231 after treated with 180055 for 24h.
250 kDa
150 kDa
100 kDa
150 kDa
75 kDa
PARP1
Ub
100 kDa
75 kDa
50 kDa
IP: PARP1
Input
37 kDa
50 kDa
Tubulin
25 kDa
37 kDa
PARP1
100 kDa
75 kDa

## Slide 15
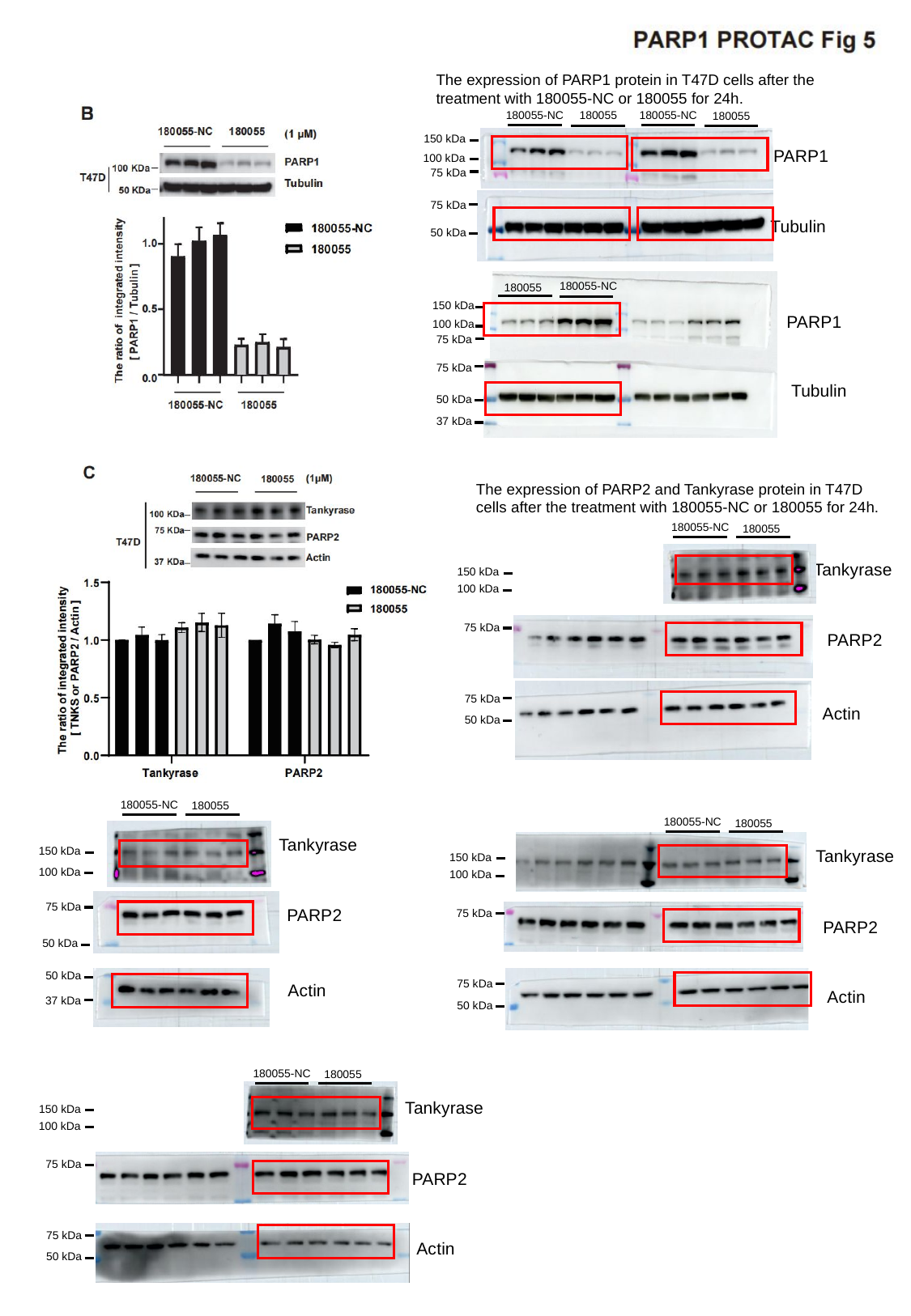

The expression of PARP1 protein in T47D cells after the treatment with 180055-NC or 180055 for 24h.
180055
180055-NC
180055-NC
180055
150 kDa
PARP1
100 kDa
75 kDa
75 kDa
Tubulin
50 kDa
180055-NC
180055
150 kDa
PARP1
100 kDa
75 kDa
75 kDa
Tubulin
50 kDa
37 kDa
The expression of PARP2 and Tankyrase protein in T47D cells after the treatment with 180055-NC or 180055 for 24h.
180055-NC
180055
Tankyrase
150 kDa
100 kDa
75 kDa
PARP2
75 kDa
Actin
50 kDa
180055-NC
180055
180055-NC
180055
Tankyrase
150 kDa
Tankyrase
150 kDa
100 kDa
100 kDa
75 kDa
PARP2
75 kDa
PARP2
50 kDa
50 kDa
75 kDa
Actin
Actin
37 kDa
50 kDa
180055-NC
180055
Tankyrase
150 kDa
100 kDa
75 kDa
PARP2
75 kDa
Actin
50 kDa

## Slide 16
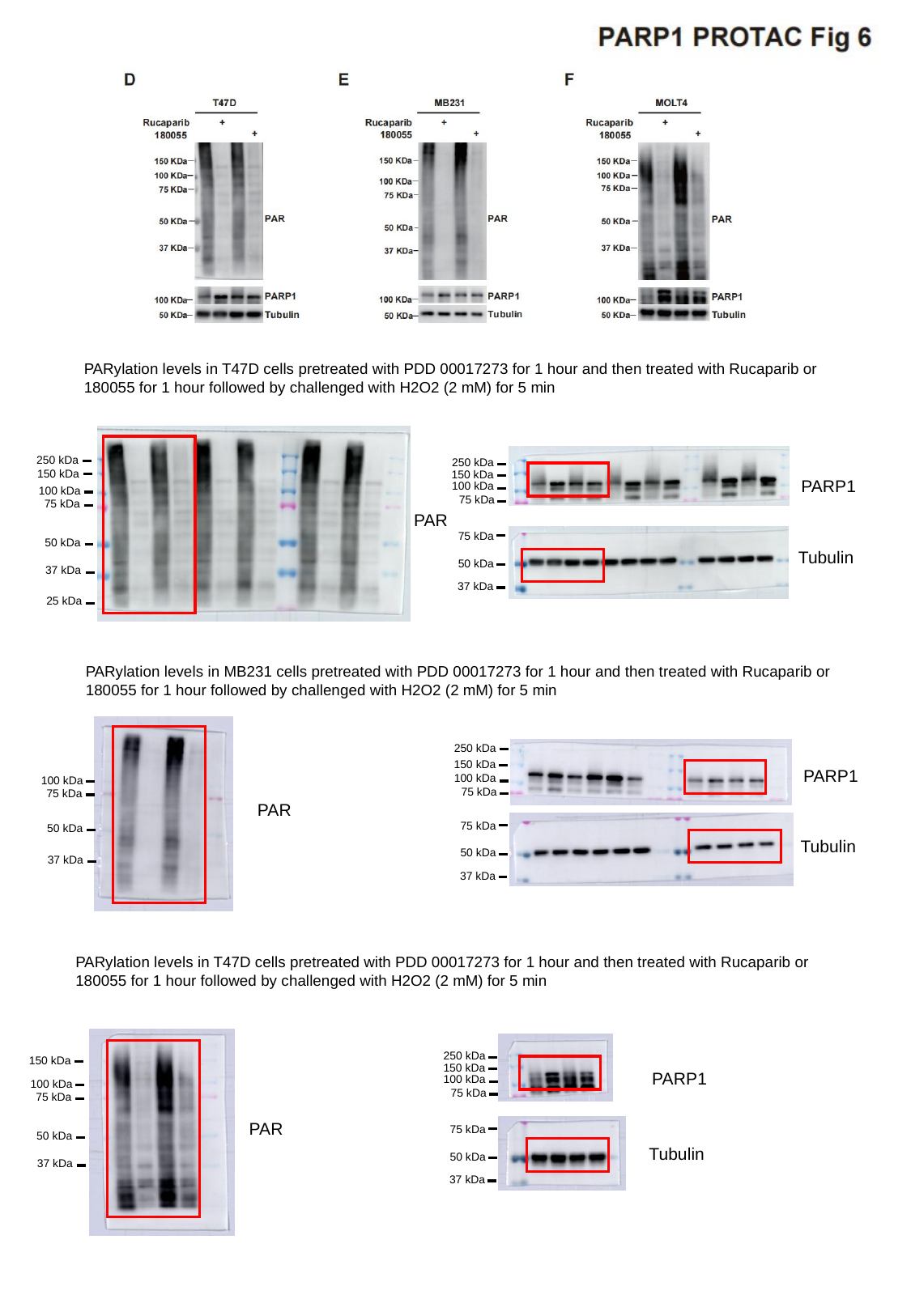

PARylation levels in T47D cells pretreated with PDD 00017273 for 1 hour and then treated with Rucaparib or 180055 for 1 hour followed by challenged with H2O2 (2 mM) for 5 min
250 kDa
250 kDa
150 kDa
150 kDa
PARP1
100 kDa
100 kDa
75 kDa
75 kDa
PAR
75 kDa
50 kDa
Tubulin
50 kDa
37 kDa
37 kDa
25 kDa
PARylation levels in MB231 cells pretreated with PDD 00017273 for 1 hour and then treated with Rucaparib or 180055 for 1 hour followed by challenged with H2O2 (2 mM) for 5 min
250 kDa
150 kDa
PARP1
100 kDa
100 kDa
75 kDa
75 kDa
PAR
75 kDa
50 kDa
Tubulin
50 kDa
37 kDa
37 kDa
PARylation levels in T47D cells pretreated with PDD 00017273 for 1 hour and then treated with Rucaparib or 180055 for 1 hour followed by challenged with H2O2 (2 mM) for 5 min
250 kDa
150 kDa
150 kDa
PARP1
100 kDa
100 kDa
75 kDa
75 kDa
PAR
75 kDa
50 kDa
Tubulin
50 kDa
37 kDa
37 kDa

## Slide 17
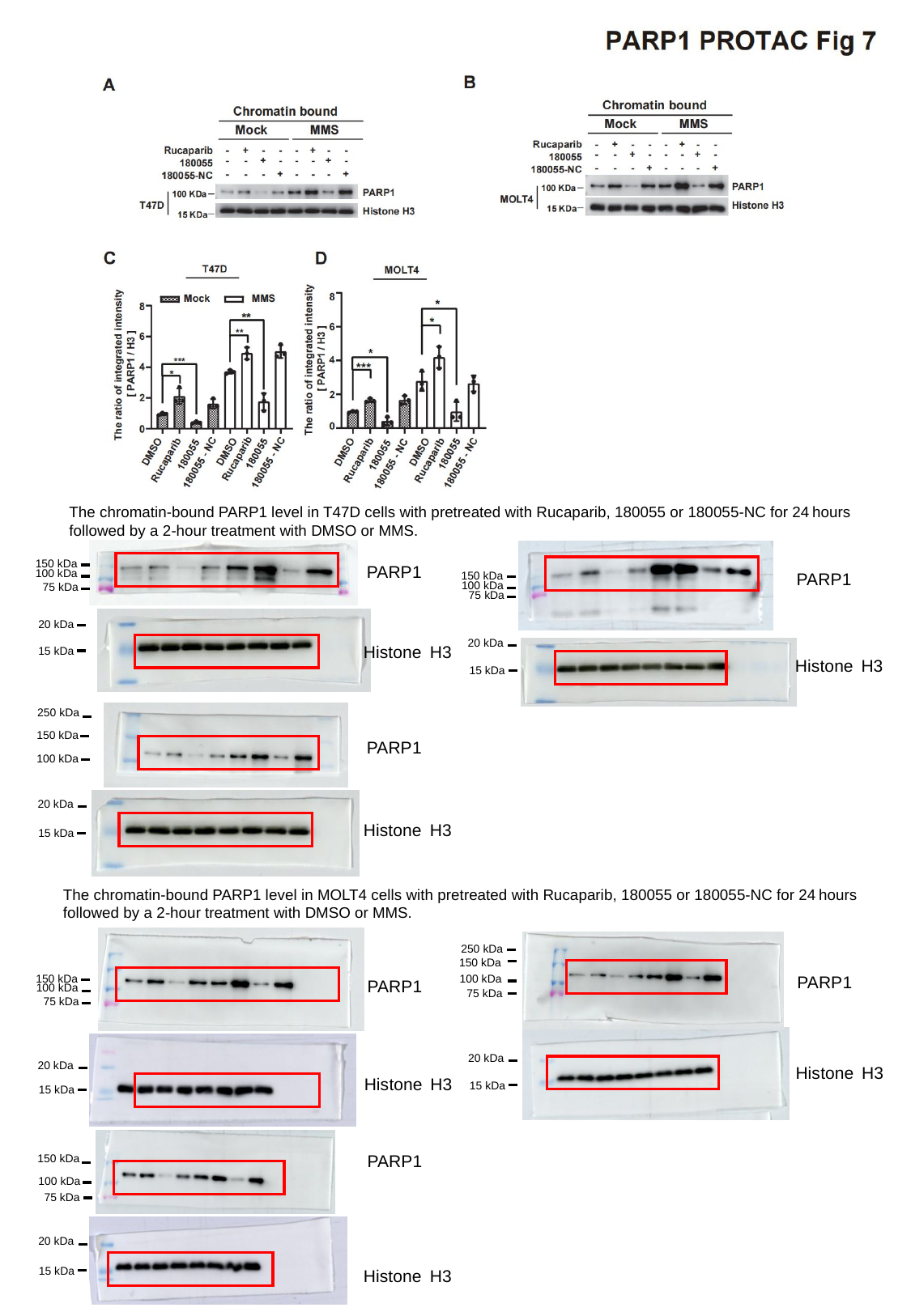

The chromatin-bound PARP1 level in T47D cells with pretreated with Rucaparib, 180055 or 180055-NC for 24 hours followed by a 2-hour treatment with DMSO or MMS.
150 kDa
PARP1
100 kDa
150 kDa
PARP1
100 kDa
75 kDa
75 kDa
20 kDa
Histone H3
20 kDa
Histone H3
15 kDa
15 kDa
250 kDa
150 kDa
PARP1
100 kDa
20 kDa
Histone H3
15 kDa
The chromatin-bound PARP1 level in MOLT4 cells with pretreated with Rucaparib, 180055 or 180055-NC for 24 hours followed by a 2-hour treatment with DMSO or MMS.
250 kDa
150 kDa
150 kDa
100 kDa
PARP1
PARP1
100 kDa
75 kDa
75 kDa
Histone H3
20 kDa
20 kDa
Histone H3
15 kDa
15 kDa
PARP1
150 kDa
100 kDa
75 kDa
20 kDa
Histone H3
15 kDa

## Slide 18
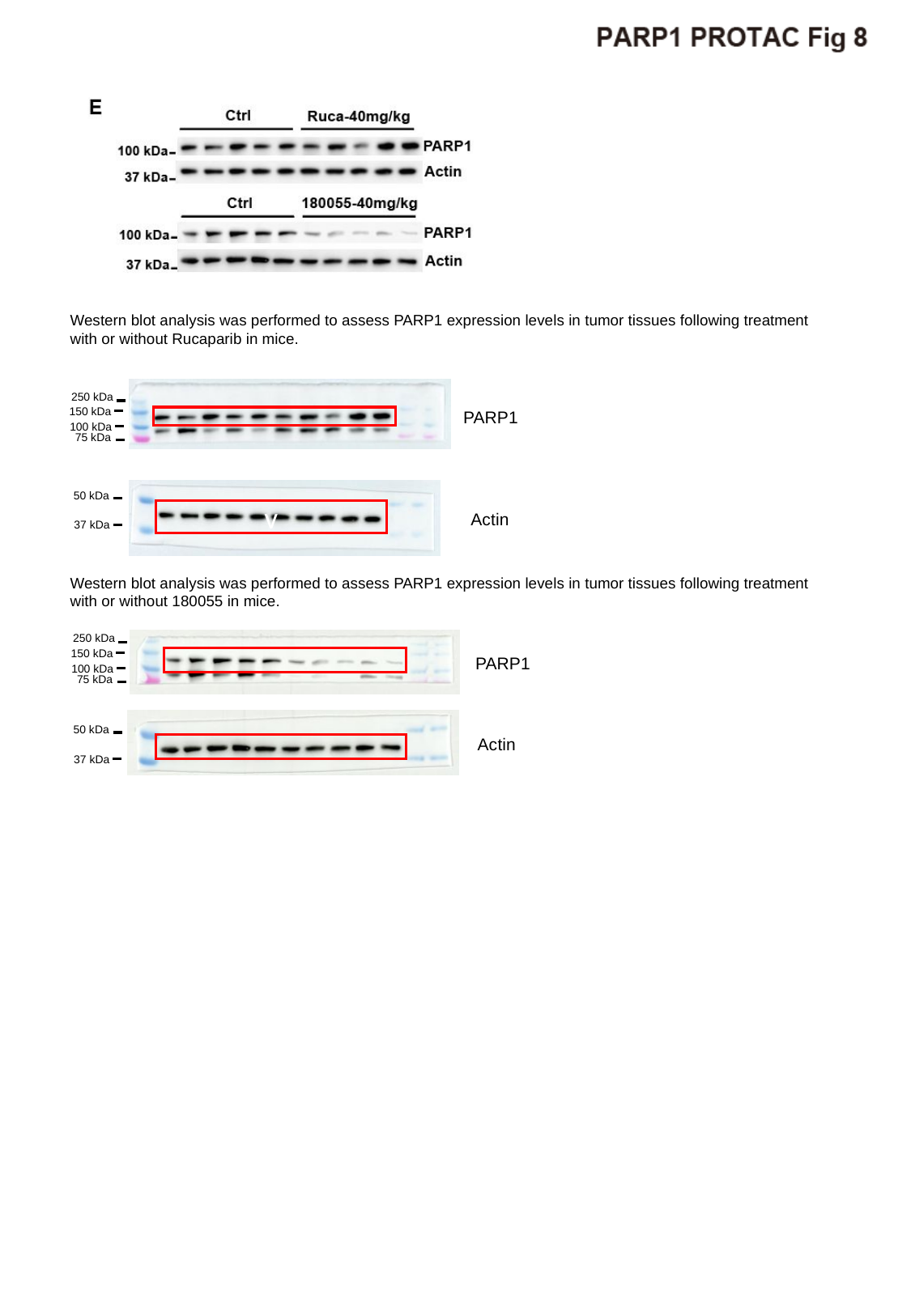

Western blot analysis was performed to assess PARP1 expression levels in tumor tissues following treatment with or without Rucaparib in mice.
250 kDa
150 kDa
PARP1
100 kDa
75 kDa
50 kDa
v
Actin
37 kDa
Western blot analysis was performed to assess PARP1 expression levels in tumor tissues following treatment with or without 180055 in mice.
250 kDa
150 kDa
PARP1
100 kDa
75 kDa
50 kDa
Actin
37 kDa

## Slide 19
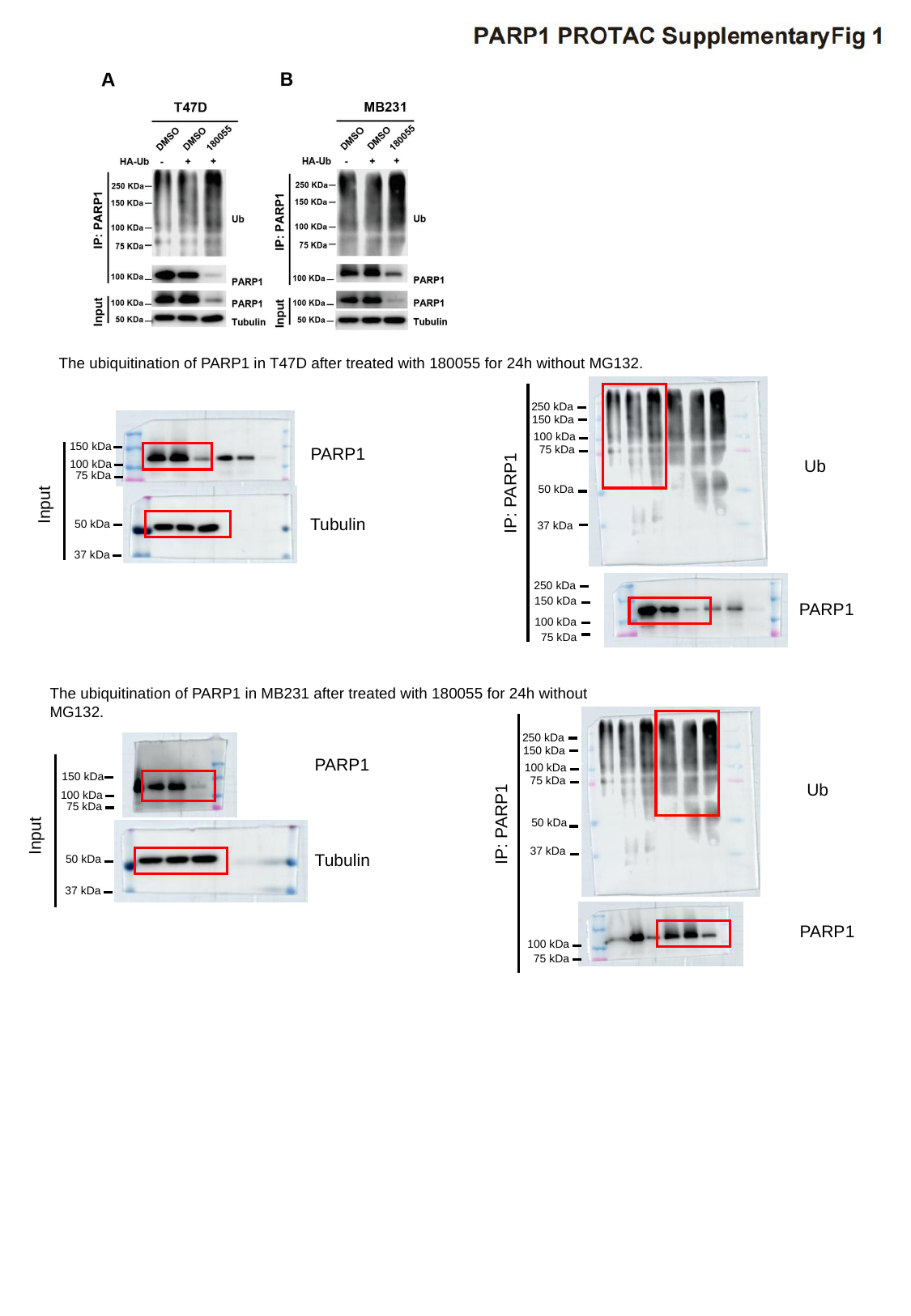

The ubiquitination of PARP1 in T47D after treated with 180055 for 24h without MG132.
250 kDa
150 kDa
100 kDa
150 kDa
75 kDa
PARP1
Ub
100 kDa
75 kDa
IP: PARP1
50 kDa
Input
Tubulin
50 kDa
37 kDa
37 kDa
250 kDa
150 kDa
PARP1
100 kDa
75 kDa
The ubiquitination of PARP1 in MB231 after treated with 180055 for 24h without MG132.
250 kDa
150 kDa
PARP1
100 kDa
150 kDa
75 kDa
Ub
100 kDa
75 kDa
IP: PARP1
50 kDa
Input
37 kDa
Tubulin
50 kDa
37 kDa
PARP1
100 kDa
75 kDa

## Slide 20
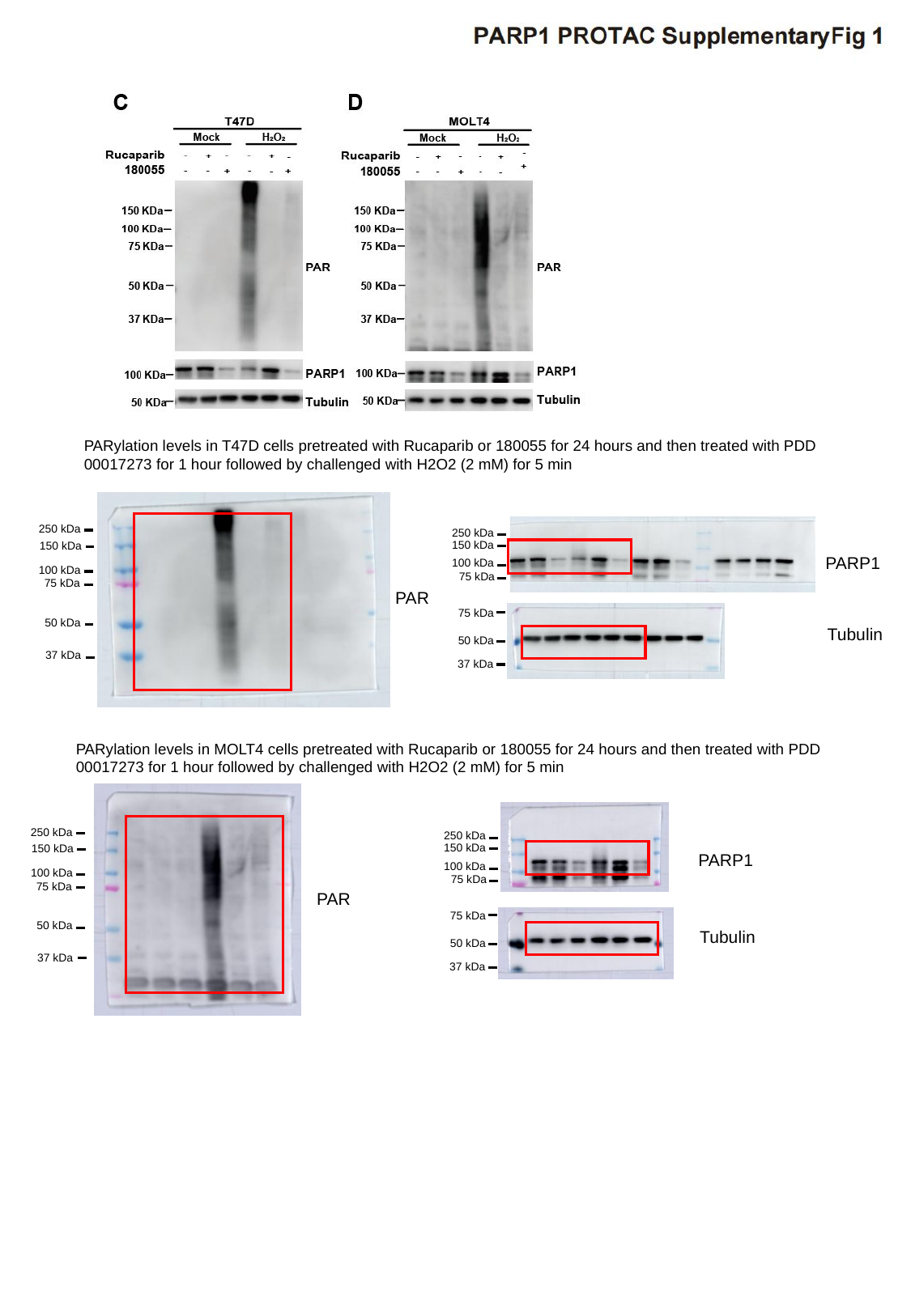

PARylation levels in T47D cells pretreated with Rucaparib or 180055 for 24 hours and then treated with PDD 00017273 for 1 hour followed by challenged with H2O2 (2 mM) for 5 min
250 kDa
250 kDa
150 kDa
150 kDa
PARP1
100 kDa
100 kDa
75 kDa
75 kDa
PAR
75 kDa
50 kDa
Tubulin
50 kDa
37 kDa
37 kDa
PARylation levels in MOLT4 cells pretreated with Rucaparib or 180055 for 24 hours and then treated with PDD 00017273 for 1 hour followed by challenged with H2O2 (2 mM) for 5 min
250 kDa
250 kDa
150 kDa
150 kDa
PARP1
100 kDa
100 kDa
75 kDa
75 kDa
PAR
75 kDa
50 kDa
Tubulin
50 kDa
37 kDa
37 kDa
